# Single-Cell Characterization of the *Frizzled 5* (*Fz5*) Mutant Mouse and Human Persistent Fetal Vasculature (PFV)

**DOI:** 10.1167/iovs.64.3.8

**Published:** 2023-03-03

**Authors:** Yuanyuan Chen, Cheng Wu, Shanzhen Peng, Dianlei Guo, Hong Ouyang, Yanhong Wei, Rong Ju, Xiaoyan Ding, Zhi Xie, Chunqiao Liu

**Affiliations:** 1 State Key Laboratory of Ophthalmology, Zhongshan Ophthalmic Center, Sun Yat-Sen University, Guangzhou, China; 2 Guangdong Provincial Key Laboratory of Food, Nutrition and Health, Department of Toxicology, School of Public Health, Sun Yat-Sen University, Guangzhou, China

**Keywords:** persistent fetal vasculature, single-cell rnaseq, hyalocytes, melanocytes, vitreous

## Abstract

**Purpose:**

Persistent fetal vasculature (PFV) is a pathological condition accounting for 4.8% of children's blindness in the United States. However, the PFV cell composition and pathogenetic mechanisms are poorly understood. This study aims to characterize PFV cell composition and associated molecular features and attempts to lay a foundation for further understanding the disease.

**Methods:**

Immunohistochemistry was conducted to characterize cell types at the tissue level. Single-cell RNA sequencing (sc-RNAseq) was performed on the vitreous cells derived from normal and Fz5 mutant mice at two early postnatal ages and human PFV samples. Bioinformatic tools were used to cluster cells and analyze their molecular features and functions.

**Results:**

The findings of this study are as follows: (1) a total of 10 defined and one undefined cell types were characterized in both the hyaloid vessel system and PFV by sc-RNAseq and immunohistochemistry; (2) neural crest-derived melanocytes, astrocytes, and fibroblasts were specifically retained in the mutant PFV; (3) *Fz5* mutants were found to possess more vitreous cells at early postnatal age 3 but returned to similar levels as the wild type at postnatal age 6; (4) altered phagocytic and proliferation environments and cell-cell interactions were detected in the mutant vitreous; (5) the human PFV samples shared fibroblast, endothelial and macrophage cell types with the mouse, but having distinct immune cells including T cells, NK cells and Neutrophils; and last, (6) some neural crest features were also shared between certain mouse and human vitreous cell types.

**Conclusions:**

We characterized PFV cell composition and associated molecular features in the *Fz5* mutant mice and two human PFV samples. The excessively migrated vitreous cells, intrinsic molecular properties of these cells, phagocytic environment, and cell-cell interactions may together contribute to PFV pathogenesis. Human PFV shares certain cell types and molecular features with the mouse.

##  

The hyaloid vessel system (HV) is a transient vasculature system developed within the embryonic eye cup supporting the embryonic lens and retina. HV forms between embryonic day 10.5 (E10.5) to E13.5 in mice^1^ and four to six weeks of gestation (WG) in humans.^2^ It gradually regresses when intraretinal vessels start to form, beginning at postnatal day 3 (P3) and 12 WG for mice and humans, respectively.^2^^,^^3^ The HV ultimately degenerates by P21 in mice and 36 WG in humans,^4^^–^^9^ rendering clearance to the optic medium for the light pass. The retina's nutrients are thereby supplied by newly formed permanent retinal vessels.

HV regression is programmed with complex genetic factors. Many mutant mice with disrupted genes functioning in various aspects of retinal development have similar PFV manifestations.^1^ For example, knockout mice of *Neogenin* in the neural crest cells,^10^ *Ephrin-A5* in the frontal eye segment,^11^ and *Fz5* in the neural retina^12^^,^^13^ all lead to retrolental pigmental mass, a PFV manifestation. Defective astrocytes in *Nuc1* mutant rats and *Large* mutated mice also show PFV phenotypes.^14^^,^^15^ Deletion of *Opn4* in ganglion cells or *Vegfr2* in retinal neurons^3^^,^^16^ prevent hyaloid components from involution. Other prominent genetic mutations causing syndromic PFV are Wnt signaling components *NPD* and *FZD4* in the blood vessels and the transcription factor *ATOH7* in ganglion cells of both murine and human retina.^17^^–^^20^ In addition, environmental factors such as oxygen and light exposures also affect HV regression.^3^^,^^21^

Regardless of its complex genetic contributions, PFV etiologies appear to converge on two crucial points: altered vitreous production or distribution of VEGF and the phagocytic activity of the macrophages. Genetic manipulations of retinal neurons cause increased vitreous VEGF, which impedes HV remodeling and leads to PFV formation.^3^^,^^16^ On the other hand, ablation of vitreous macrophages or macrophage-endothelial interactions prevents HV from macrophage-induced apoptosis.^22^^,^^23^ Aberrant vitreous migration of astrocytes ensheathing the HV also blocks macrophage-endothelial cell interactions, thus the HV regression.^14^^,^^24^ Nevertheless, the timely programmed HV regression appears rather nonautonomous than inherent, consistent with the observations that PFV formation in many mutant mice (as aforementioned) is non-cell autonomous.

Another scenario of PFV formation is the early excess vitreous cell migration. The HV precursors are mainly extraocular mesenchymal cells of mesodermal and neural crest origins.^25^^–^^27^ These cells enter the prospective vitreous cavity through the optic fissure, a tissue gap in the developing ventral retina, and the annular opening between the optic cup and lens primordium.^2^^,^^28^ Accordingly, abnormal vitreous neural crest migration has been reported in *Neogenin* and *Ephrin-A5* mice through the faulty optic fissure and annular opening, forming pigmentary retrolental mass surrounding the hyaloid vessels.^10^^,^^11^ The regression of the vitreous components in these mice is beyond hyaloid vessels and thus unconventional.

Wnt signaling through Frizzled receptors and coreceptors plays essential roles in multiple biological processes in the retina, including vascular development,^20^^,^^29^^–^^31^ retinal neurogenesis,^30^ and differentiation of retinal pigmental epithelium.^32^ Previously, we and others have shown that mutation of one of *Frizzleds*, *Fz5*, in mice causes microphthalmia and defective optic fissure with nonautonomous PFV manifestation,^12^^,^^13^^,^^33^^,^^34^ similar to the *Neogenin* neural-crest knockout mice.^10^ However, *Fz5* is not expressed in the neural crest or vitreous cells (Supplementary Figs. S1A–C), and either constitutively or specifically knocking out *Fz5* in retina leads to vitreous PFV formation, which is thus nonautonomous.^12^^,^^13^ It remains largely unclear about the cell composition of the vitreous retrolental mass of the *Fz5* mutant mice. Moreover, the origin of pigment cells in the mutant vitreous is yet to be determined. We hypothesized that the vitreous cell composition and relevant molecular features bear insights into PFV pathogenesis. Thus, in this study, we first characterized the *Fz5* PFV cells with known molecular markers, then performed single-cell RNA sequencing (sc-RNAseq) to document cell types and clusters. We additionally sequenced two human patient vitreous samples with PFV manifestations and compared cellular similarities between mouse and human PFV. The results laid a foundation for further understanding the PFV disease.

## Methods

### Mouse Breeding

All procedures involving mice were approved by the Animal Care and Use Committee, Zhongshan Ophthalmic Center, and adhered to ARVO Statement for the Use of Animals in Ophthalmic and Vision Research. *Fz5* conditional and germline knockout mice were generated as described previously.^12^ *Fz5* null allele with the coding exon replaced by a *lacZ* reporter (*Fz5^lacZ/+^*) was crossed onto *Six3-Cre* transgenic background to obtain *Fz5^lacZ/+^*; *Six3-Cre* mice. *Fz5^lacZ/+^*; *Six3-Cre* mice were then crossed with homozygous *Fz5* conditional knockout mice with a knocked-in alkaline phosphatase (AP) reporter (*Fz5^CKO-AP/CKO-AP^*) to delete *Fz5* in *Six3-Cre* expressing tissues. The resulted genotype *Six3-Cre*;*Fz5^AP/^ ^lacZ^* was designed as *Fz5^−^^/^^−^*_._ Mice carrying two wildtype alleles of *Fz5* were used as controls (*Fz5^+/+^*).

### Human Samples

The human PFV vitreous materials were collected at Zhongshan Ophthalmic Center, Guangzhou. The specimens were obtained from patients clinically diagnosed with PFV pathology and undergoing surgical interventions. The two specimens are age 11 months and 20 days and one year and nine months old, respectively. Informed consent was obtained from the subjects after the nature and possible consequences of the study were explained. All human study protocols were reviewed and approved by the Institutional Review Board of the Zhongshan Ophthalmic Center. Considering the average life spans for mice and humans are 2 and 80 years, respectively, the two human specimens would be roughly equal to the P9 and P16 mice.

### Cell Dissociation and Sample Preparation for Single-Cell RNA Sequencing

Four PFV and HV tissue samples for each age of P3 and P6 were dissected from *Fz5−/−* and *Fz5+/+* mice, respectively. The samples were transferred to 100 mL 0.25% trypsin-EDTA and digested at 37°C for 15 minutes with pipetting intermittently. After quenching trypsin activity with DMEM containing 10% serum, the dissociated cells were collected by centrifugation at 100 rcf for five minutes and resuspended in Ca2+/Mg2+ free PBS to make a final concentration at ∼1200 cells/mL. Human PFV tissue samples were surgically collected from patients into ice-cold PBS, spun in a centrifuge at 200 *g* for two minutes, and processed with the same protocol as for mouse PFV described above.

Cell viability was estimated with trypan blue staining, and live cells were counted by use of a Countess II Cell Counter (Thermo Fisher Scientific, Waltham, MA, USA). Samples having >90% live cells were subjected to library construction. Cellular suspensions were loaded on a Chromium Single Cell Instrument (10x Genomics, Pleasanton, CA, USA) for genome-scale metabolic model generation and barcoding. Barcoded libraries were pooled and sequenced on Illumina Hi-Seq 2500 platform (Illumina, San Diego, CA, USA), generating 150-bp paired-end reads.

### Data Processing and Analysis

For each sample of the mouse vitreous, FASTQ files were processed by using Cell Ranger (v.3.0.2, https://support.10xgenomics.com/) count pipeline coupled with mouse reference (mm10) to generate feature-barcode matrices. Further quality control was achieved by the Seurat package (v3.2.0; https://github.com/satijalab/seurat) to select cells expressing 200 to 6000 genomic genes and less than 15% mitochondria genes. Meanwhile, genes expressed in less than five cells were eliminated. Principal component analysis was performed (2000 features) to reduce the dimensionality of the dataset. The first 30 principal components were used as inputs for graph-based clustering, and resolution value 1.2 was chosen to discriminate clusters. T-distributed stochastic neighbor embedding or UMAP (uniform manifold approximation and projection) was used to visualize the clusters. Marker genes of each cluster were obtained by implementing the FindAllMarkers function by comparing gene expression of that cluster to all others combined (Wilcoxon test) with selection conditions that genes were expressed in no less than 25% of cells of the cluster. The relative log-fold change to other clusters is set above 0.25 (log [FC] ≥ 0.25). The cell types were annotated based on a bioinformatic clustering database (http://biocc.hrbmu.edu.cn/CellMarker/; https://panglaodb.se/) and known marker genes from experimental data published in the literature.

Initial visualization by t-distributed stochastic neighbor embedding gave rise to 42 cell clusters constituting 14 cell types. Cells with lens, retina, and peripheral retina (ciliary marginal zone) identities were excluded as dissection contaminations. The remaining cells were subjected to a second round of clustering analysis, giving 32 clusters.

Gene ontology (GO) enrichment analysis was performed using the cluster Profiler package.^35^ Pearson's correlation coefficients were calculated by comparing each cluster's mean RNA expression levels. Cellular interactions were inferred from ligand-receptor communications identified by CellPhoneDB v.2.0 (a public human repository),^36^ and mouse genes were mapped to their human homologs using BioMart.^37^

For human PFV tissues, the BD Rhapsody system (BD Bioscience, Franklin Lakes, NJ, USA) was used to capture the transcriptomic information of the single cells. Single-cell capture was achieved by a random distribution of a single-cell suspension across >200,000 microwells through a limited dilution approach. Beads with oligonucleotide barcodes were added to saturation to ensure a bead paired with a cell in a microwell. The cells were lysed in the microwell to hybridize mRNA molecules to barcoded oligos on the beads. Beads were collected into a single tube for reverse transcription and ExoI digestion. Upon cDNA synthesis, each cDNA molecule was tagged at the 5′-end (that is, the 3′-end of an mRNA transcript) with a unique molecular identifier (UMI) and cell barcode indicating its cell of origin. Whole transcriptome libraries were prepared using the BD Rhapsody single-cell whole-transcriptome amplification workflow, including random priming and extension (RPE), RPE amplification PCR, and whole-transcriptome amplification index PCR. The libraries were quantified using a high-sensitivity DNA chip (Agilent Technology, Tokyo, Japan on a Bioanalyzer 2200 and the Qubit High Sensitivity DNA assay (Thermo Fisher Scientific). Sequencing was performed by Illumina sequencer on a 150 bp paired-end run.

ScRNA-seq data analysis was performed using the NovelBrain Cloud Analysis Platform (Novel Bio Co., Ltd., Bangkok, Thailand). Fastp was applied to filter the adaptor sequence and remove the low-quality reads with the default parameter setting. UMI tools were applied for single cell transcriptome analysis to identify the cell barcode whitelist. The UMI-based clean data were mapped to the human genome (Ensemble version 100) using STAR mapping with customized parameters from the UMI tools standard pipeline to obtain the UMI counts. We used the Seurat package for analysis with the same parameters as above.

### Data Availability

Raw and processed data files from mouse and human vitreous for this study are available at GSE203116. The human vitreous raw (FASTQ) data will be provided for scientific research on request complying with the law due to human patient privacy concerns.

### Histology, Immunohistochemistry, and In Situ Hybridization

For postnatal mice, eyeballs were enucleated from the eye sockets and fixed in 4% paraformaldehyde for 15 minutes at room temperature (RT), followed by corneal removal and fixation for another 45 minutes. For embryos, embryonic heads were fixed in 4% paraformaldehyde overnight at 4°C. Graded dehydration was performed by placing tissues through 10%, 20%, and 30% sucrose in PBS under RT. Cryosections of 15 µm were prepared using a Leica cryostat CM 1900 (Leica, Wetzlar, Germany). For immunohistochemistry, tissue sections were incubated with primary antibodies overnight at 4°C, and secondary antibodies were incubated at room temperature for two hours. The primary antibodies used for this study were rabbit anti-Col1a1 (1:500, A1352; ABclonal Technology, Woburn, MA, USA), rat anti-CD31 (1:500, 557355; BD Bioscience), rabbit anti- AP2β (1:200, 2509S; Cell Signaling Technology, Danvers, MA, USA), mouse anti-alkaline phosphatase (1:100, 14-9870-82; Invitrogen, Carlsbad, CA, USA), rabbit anti-Pax3 (1:100, bs-1097R; Bioss Antibodies Inc., Woburn, MA, USA), goat anti-Sox10 (1:20, AF2864; R&D Systems, Minneapolis, MN, USA), and mouse anti-Tyrp1 (1:100, ab3312; Abcam, Cambridge, MA, USA). GS-IB4 (Alexa-488 conjugated; Thermo Fisher Scientific) (I21411; Invitrogen) was used to stain blood vessels. Secondary antibodies were donkey anti-rabbit Alexa488 IgG (1:500, SA5-10038; Thermo Fisher Scientific), donkey anti-rabbit Alexa568 IgG (1:500, A10042, Life Technologies, Carlsbad, CA, USA), donkey anti-goat Alexa568 IgG (1:500, A11057; Life Technologies), donkey anti-mouse Alexa488 IgG (1:500, A21202; Life Technologies), and donkey anti-mouse Alexa568 IgG (1:500, A10037, Life Technologies). Images were acquired using an upright microscope (ApoTome.2; Zeiss, Oberkochen, Germany).

HV isolation was as described by Lobov et al.^22^ Briefly, fixed eyeballs were transferred to Eppendorf tubes containing 10% gelatin (abs9357; Absin, Shanghai, China), inverted gently to allow complete immersion. The eyeballs were then transferred onto a glass slide with the optic-disc side contacting the slide. Samples were left dry for five to 10 minutes, and the scleral, choroidal, and retinal layers were peeled off with forceps, separating from the remaining HV tissue. Next, the gelatin-embedded HV samples were heated at 60°C, the melted gelatin was absorbed with Kimwipes (Fisher Scientific), and immunohistochemistry was performed on dried samples.

For in situ hybridization, full-length Fz5 cDNA was PCRed from a pRK5 expression vector, with RNA polymerase binding sites T3 and T7 flanked for antisense and sense RNA probes, respectively. Tissue preparation and in situ hybridization followed the protocol described by Ru et al. ^38^

### Transmission Electron Microscopy

Transmission electron microscopy followed the protocol described by Guo et al.^39^ Ultrathin slices of 50 nm were stained with uranyl acetate and viewed under a transmission electron microscope (H-7500; Hitachi, Tokyo, Japan).

## Results

### PFV of the *Fz5* Mutants Express Neural Crest and Astroglia Markers

**Figure 1.**
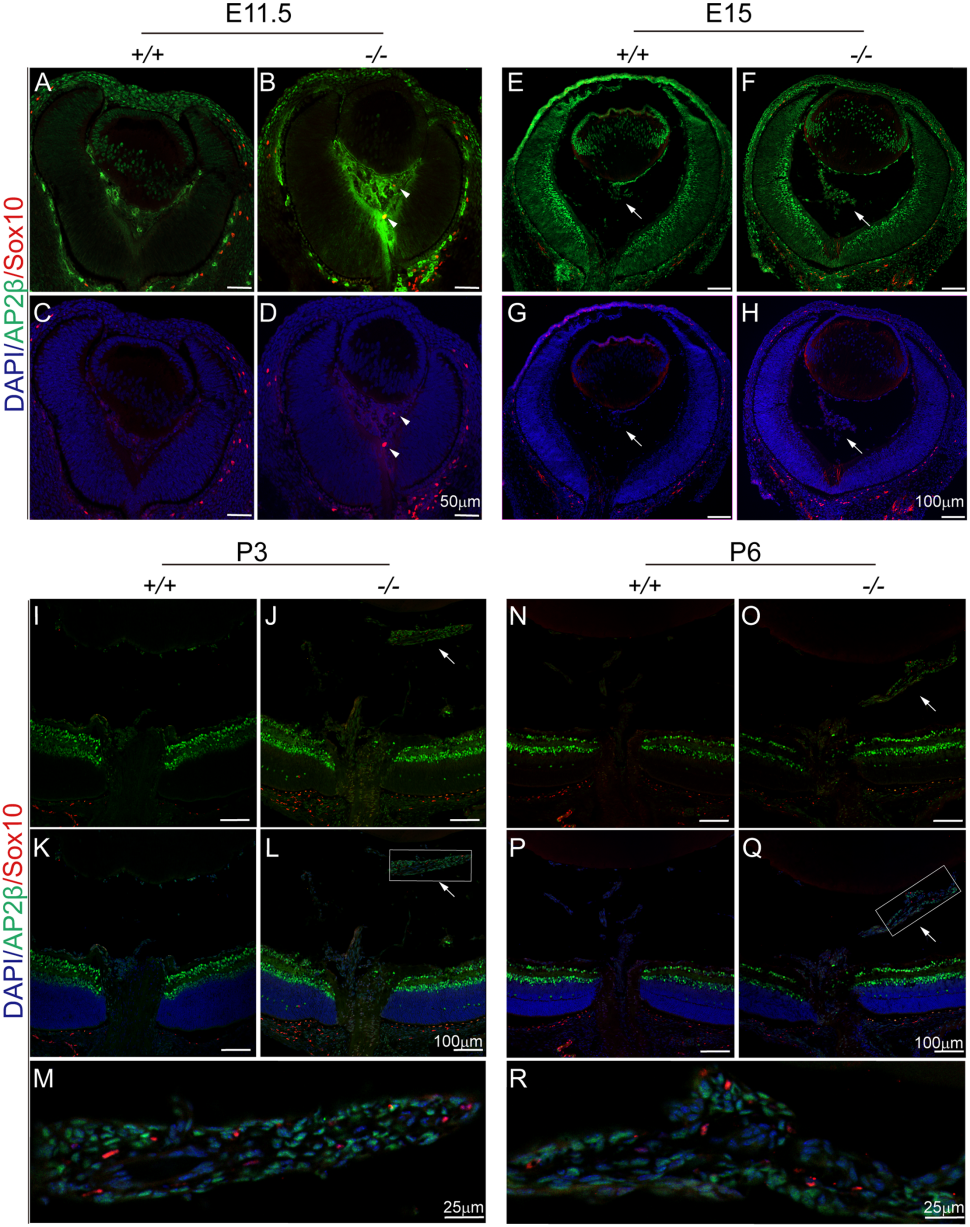
Expression of neural crest markers Sox10 and AP2β in the vitreous. Throughout the figure panels, Sox10 is in *red*, and AP2β is in *green*. “+/+”, wild-type mice (*Fz5*^+/+^); “−/−”, mutant mice (*Fz5*^−/−^). (**A–D**) Sox10 and AP2β expression at E11.5. *Arrows* point to the Sox10 positive cells. (**E–H**) E15 eye sections. *Arrows* point to the forming hyaloid vitreous. Noting more cells in the mutant vitreous at either E11.5 or E15. (**I–M**) P3 retina. *Arrows* point to the retrolental mass. (**M**) is a magnified boxed area in (**L**). (**N–R**) P6 retina. *Arrows* point to the retrolental mass. (**R**) is a magnified boxed area in (**Q**), which is horizontally orientated.

The PFV phenotype in the *Fz5* mutant mice has been reported previously.^12^^,^^13^ The existence of pigmentary retrolental mass prompted us to examine whether it is derived from the neural crest cells. AP2b, commonly used for tracing neural crest lineage, was expressed in the mutant vitreous mesenchymal cells at E11.5 (Figs. 1A, 1B). Meanwhile, staining of Sox10, another more restricted and melanocyte-associated neural crest marker, was mainly localized to the prospective choroid layer (Figs. 1C, 1D) and sometimes found in the mutant vitreous only. At E15, although equally expressed in the inner retina of both wild-type and mutant mice, AP2b was mainly detected in the mutant retrolental mass and a few wild-type vitreous cells (Figs. 1E, 1F). Outside the choroid layer (Figs. 1G, 1H), Sox10 expression appeared very weakly in the vitreous mass (Fig. 1H). At P3 and P6, despite the comparable inner retina staining, AP2b primarily stained the mutant but not the wild-type vitreous (Figs. 1I–Q). Sox10 was also observed in the retrolental vitreous mass of the mutants at these ages (Figs. 1J, 1L, 1O, 1Q, 1M, 1R) but only partially colocalized with AP2b.

**Figure 2.**
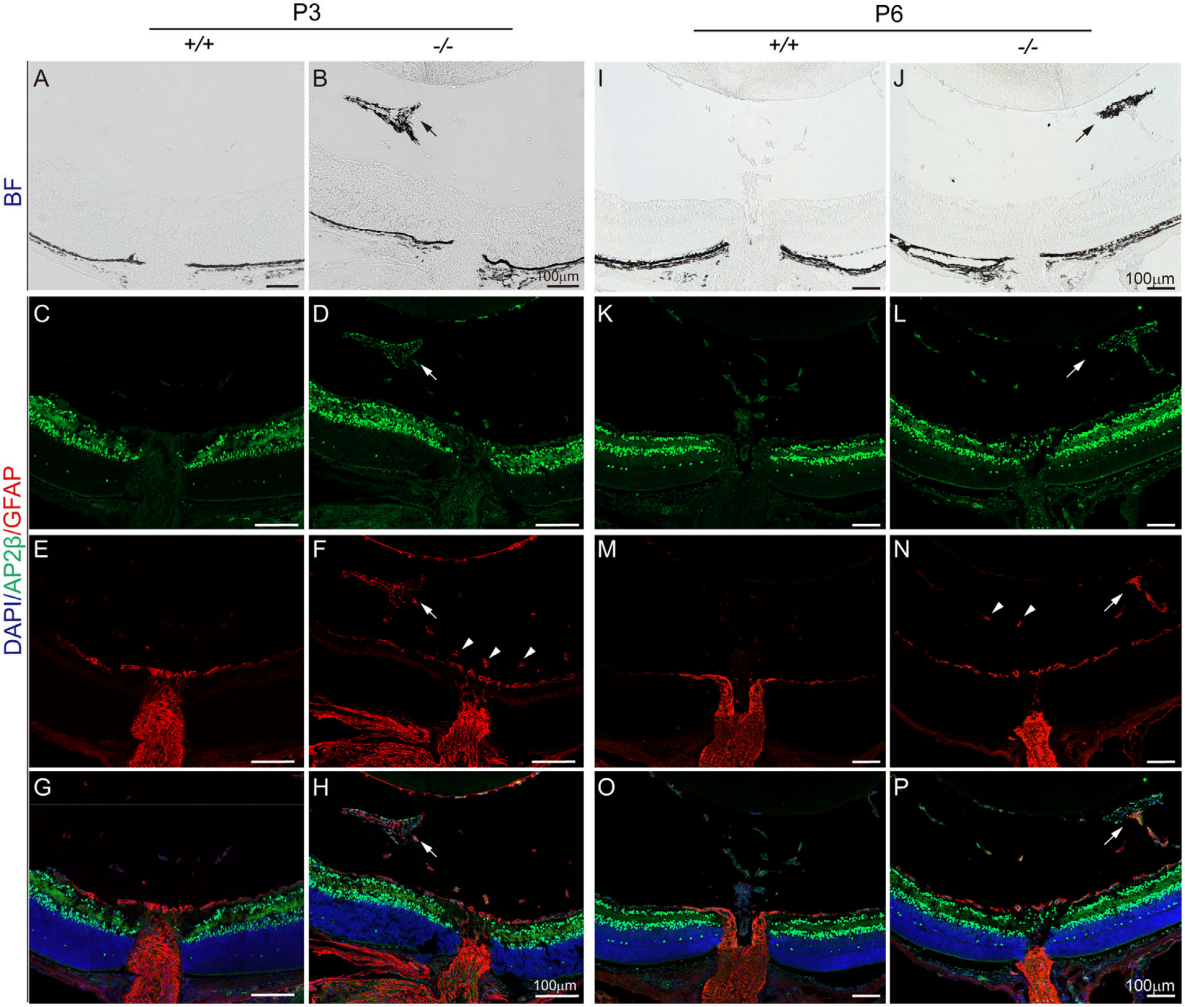
Astroglia were present in the *Fz5* mutant vitreous as indicated by GFAP. Throughout panels, GFAP is in *red*, and AP2β is in *green*. “+/+”, wild-type mice (*Fz5*^+/+^); “−/−”, mutant mice (*Fz5*^−/−^). (**A–H**) P3 retina. (**A, B**) Bright-field microscopy. *Arrows* in (**B**), (**D**), and (**F**) point to the pigmentary retrolental mass. *Arrowheads* in (**F**) point to the vitreous hyaloid vessel fragments positive for GFAP in the mutants. (**I–P**) Same panel arrangement as in (**A–H**).

We next examined whether astrocytes might also constitute *Fz5* mutant retrolental mass, as seen in several other mutants.^14^^,^^15^ Using GFAP as an astrocyte marker, we examined P3 and P6 vitreous and found GFAP-positive cells in the mutant pigmentary mass labeled by AP2b at either of the two examined ages (Figs. 2A–H, 2I–P). Furthermore, GFAP-positive astroglia intermingled with or separated from the pigment cells (Figs. 2F, 2H, 2N, 2P). Thus the *Fz5* mutant PFV contained both neural crest-derived cells and astroglia.

### The PFV Pigment Cells are Melanocytes

**Figure 3.**
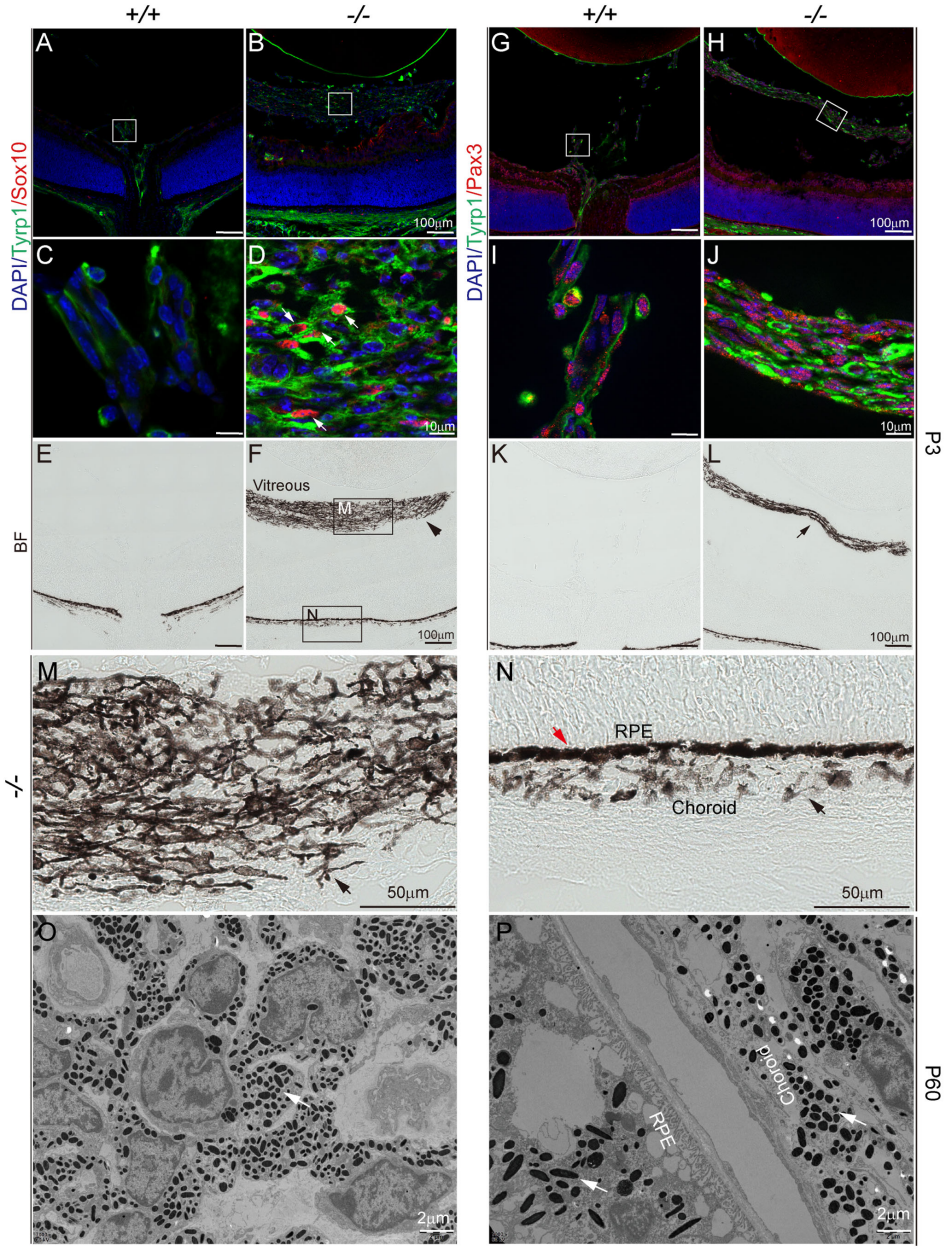
The pigment cells of the *Fz5* mutant vitreous are melanocytes. (**A–N**) P3 retinal sections. (**A–D**), Sox10 is in *red*, and Tyrp1 is in *green*. (**A–D**) Co-labeling of neural crest marker Sox10 and melanin biosynthetic enzyme Tryp1. Boxed areas (**A**) and (**B**) are magnified in (**C**) and (**D**), respectively. (**C**) Wild-type vitreous showed background staining in hyaloid vessels. (**D**) Intense Tyrp1 cytoplasmic staining colocalized with Sox10-positive cells of the vitreous mass. (**E, F**) Bright-field microscopy of the same sections of (**A, B**). *Boxed areas* are pigmentary regimental mass (**M**) and choroid melanocytes and RPE (**N**), respectively, which are magnified in (**M**) and (**N**). (**G–J**) Pax3 is in *red*, and Tyrp1 is in *green*. *Boxed areas* in (**G**) and (**H**) are magnified in (**I**) and (**J**) respectively. (**I**) Pax3 expression is detected in endothelial cells. Note that the Tryp1 staining is considered background. (**J**) Strong Tyrp1 cytoplasmic staining appeared inversely correlated with the Pax3 intensity (*arrows*). (**K, L**) Bright-field microscopy of the same sections (**G, H**). The *arrow* in (**L**) points to the regimental mass. (**M**) High magnification of vitreous pigmentary cells. (**N**) High magnification of RPE and choroid melanocytes. The *red arrow* points to RPE, and the black arrow point to choroid melanocytes. (**O, P**) Transmission electron microscopy of P60 mutant vitreous (**O**) and RPE and choroid (**P**).

Pigment cells in the eye are neural crest-derived choroid melanocytes and RPE cells.^40^ The origin of the vitreous pigment cells of the *Fz5* PFV is unclear. We therefore examined neural crest markers along with Tyrp1, a protein for melanin production, to identify neural crest–derived pigment cells. Although not found in the wild-type vitreous, Sox10-positive cells were present in the mutant vitreous expressing Tryp1 (Figs. 3A–D). Meanwhile, Pax3 was expressed nearly in all wild-type and mutant vitreous cells, and many of these mutant cells weakly expressed Tryp1 (Figs. 3G–J). Despite being a generic neural crest marker, Pax3 was reported to be expressed in blood vessel smooth muscle cells,^41^^,^^42^ and neural crest cells can differentiate into retinal pericytes as well.^42^^,^^43^ Thus it is inferred that Pax3-positive cells having no pigments are from blood vessels. Note that the wild-type blood vessel–associated Tryp1 was considered background staining from the mouse antibody used.

Bright-field microscopy showed that pigment cells were present in the mutant but not wild=type vitreous (Figs. 3E, 3F, 3K, 3L). Morphologically, the vitreous pigment cells had tubular processes (Fig. 3M), resembling the choroid melanocytes more than the RPE (Fig. 3N). Additionally, an examination of adult pigmentary tissues of the mutant vitreous using transmission electron microscopy revealed round melanin granules more similar to the choroid than the RPE (Figs. 3O, 3P). Thus, the results suggested that the mutant pigment cells were melanocytes in early postnatal and adult vitreous.

### Characterization of Normal and PFV Vitreous Cell Types by scRNA-seq

**Figure 4.**
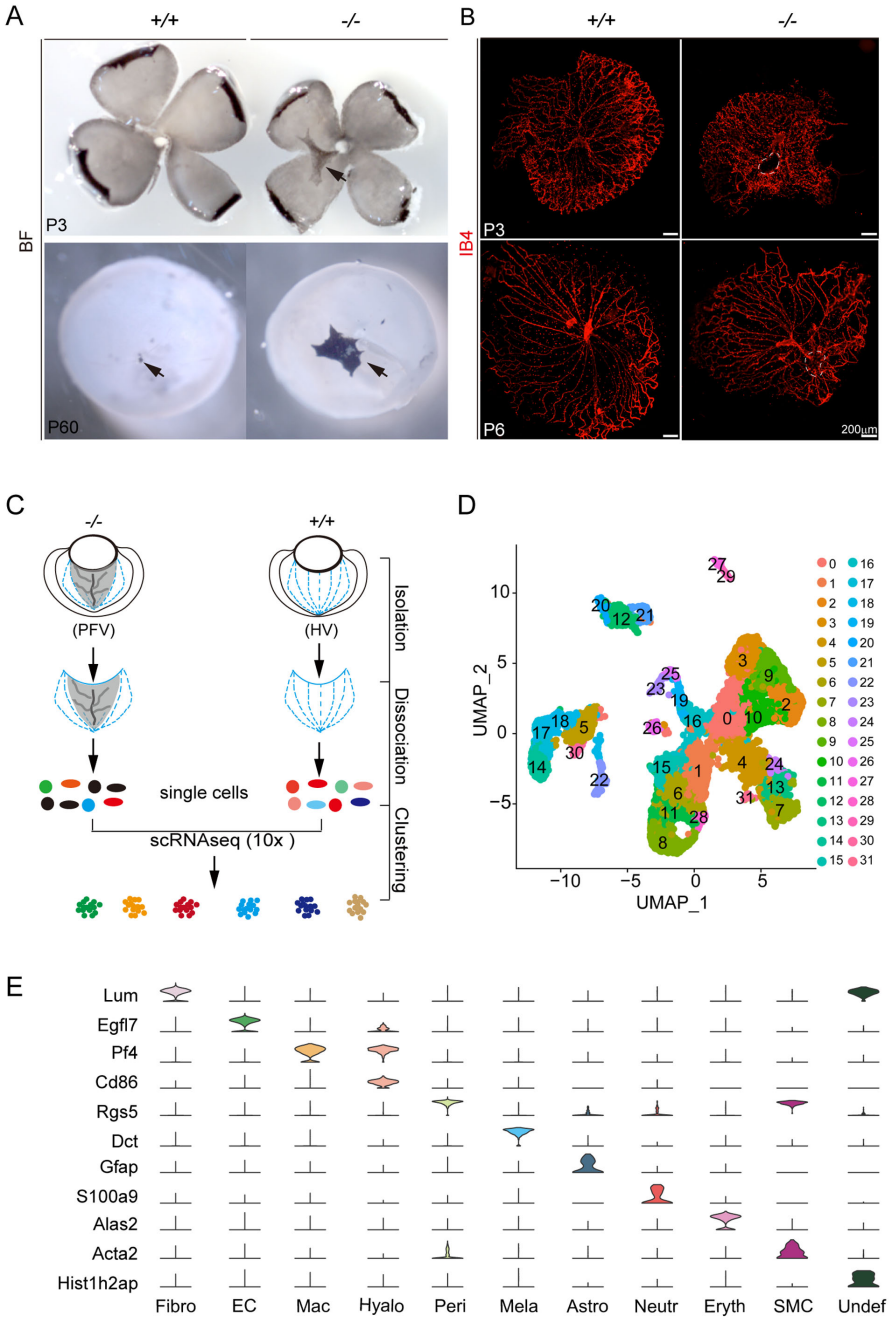
Sc-RNAseq of the mouse vitreous hyaloid vasculature at P3 and P6. (**A**) Flat-mount retinas showing the pigmented PFV tissues at P3 and P60. (**B**) IB4-stained hyaloid vessels of P3 and P6 vitreous. Circumscribed areas of the optic disc are *Fz5* mutant pigmented tissues. (**C**) Sc-RNAseq workflow. (**D**) Sc-RNAseq identified clusters projected on two-dimension UMAP. (**E**) Violin grams of marker expression of the identified cell types. Fibro, fibroblasts; EC, endothelial cells; Mac, macrophages; Hyalo, hyalocytes; Peri pericytes; Mela, melanocytes; Astro, astrocytes; Neutr, neutrophils; Eryth, erythrocytes; SMC, smooth muscle cells; Undef, undefined.

To further understand the PFV cell composition, we chose P3 and P6 vitreous to perform sc-RNAseq to compare cell types and numbers of cells between the wild types and the mutants. The mouse HV stops growing at P3^16^^,^^44^ and regresses concurrently at P4.^16^ Therefore it is expected that most, if not all, HV or PFV cell types would be identified in P3 vitreous, and many of them would regress significantly at P6.^3^ Under bright-field microscopy, *Fz5* mutants (*Fz5^−^^/^^−^*) exhibited pigmented PFV connecting with the optic disk in both early postnatal age (P3) and adulthood (P60) (Fig. 4A). IB4 staining showed a reduction of HV density from P3 to P6 in both wild-type (*Fz5^+/+^*) and the mutant (*Fz5^−^^/^^−^*) vitreous (Fig. 1B). The smaller size of the mutant HV (Fig. 4B) was due to the small eyeballs/microphthalmia of the *Fz5* mutants, which has been described in previous studies.^12^^,^^34^

A total of 32 cell clusters were identified by sc-RNAseq (Methods, Supplementary Fig. S1D, S1E, Figs. 4C, 4D) and defined by a set of top genes expressed in each cluster (Supplemental data sheet 1, Fig. E), constituting 10 cell types: the endothelial cells (EC), macrophages (Mac), hyalocytes (Hyalo), pericytes (Peri), smooth muscle cells, erythroid-like cells (Eryth), neutrophils (Neutr), in particular, astrocytes, and melanocytes (Mela), fibroblasts (Fibro), and an undefined cell type/cluster (Undef. C26) (Fig. 4E). Thus, the finding of the existence of astrocytes and melanocytes were consistent with the above marker analysis and validated the sc-RNAseq approach (Figs. 1 and 2), and the fibroblasts are found new in the mouse vitreous.

### Melanocyte Heterogeneity and Similarities to the Choroid and the RPE Cells

**Figure 5.**
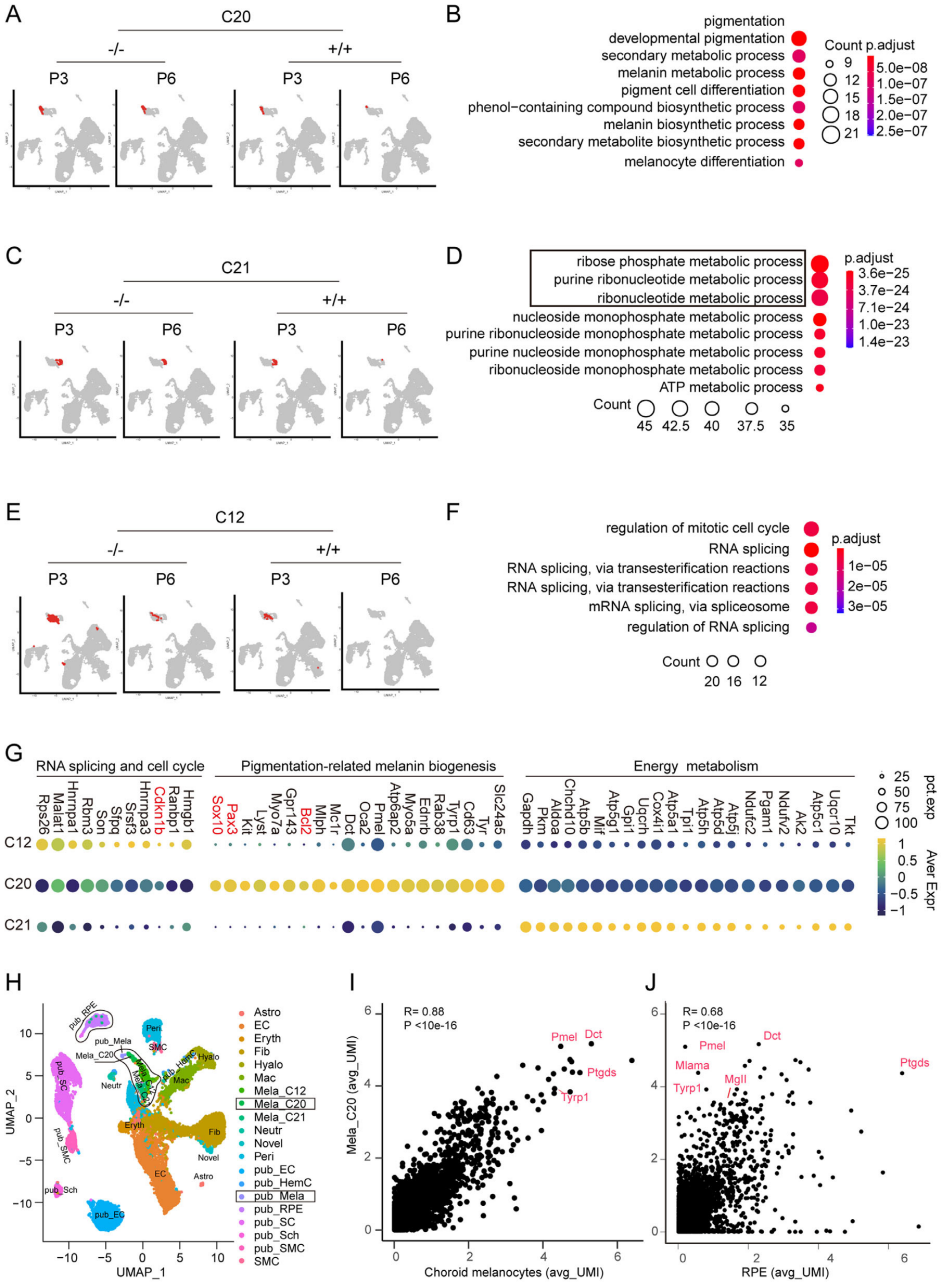
Melanocyte heterogeneity and similarities to the choroid and the RPE cells. (**A**) Melanocyte cluster C20 distribution on UMAPs. (**B**) Gene ontology (GO) terms of C20 selected from top items with *P* value <2.0e-07. (**C**) Melanocyte cluster C21 distribution on UMAPs. (**D**) GO terms of C21 selected from top items with *p*-value <1.0e-23. (**E**) Melanocyte cluster C12 distribution on UMAPs. (**F**) GO terms of C12 selected from top items with *P* value <2.0e-05. (**G**) Dot plot of subsets of differentially expressed genes associated with cluster GO terms. Gene names in red are cell cycle (*Cdkn1b*), neural crest (*Sox10* and *Pax3*), and apoptosis (*Bcl2*) genes. (**H**) RNA-seq data merged with that of mouse RPE/ retinal choroid cells (Public database: GSE135167[PMID: 32196081) and projection of the integrated data on a two-dimensional UMAP. (**I, J**) Correlation coefficient analysis of the gene expression profiles of C20 with the choroid and RPE pigment cells, respectively.

We further looked into the molecular profile of the melanocytes in terms of functional GO terms and related gene modules. Among the three identified melanocyte clusters, C20 and C21 were the retaining clusters almost exclusively for the P6 mutants (Figs. 5A, 5C). Compared with the other two clusters, C20 showed GO items more relevant to melanin production and strongly expressed neural crest markers Sox10 and Pax3 (Figs. 5B, 5G). C21 showed an active nucleotide biosynthesis (ribose phosphate and purine ribonucleotide metabolic process), which is involved in a range of cell activities, including DNA synthesis of the cell cycle (Figs. 5D, 5G). Notably, C20 highly expressed neural crest transcription factors, Sox10 and Pax3, and anti-apoptotic factor, Bcl2 (Fig. 5G). C12 was also unique to the mutant vitreous at P3, having a small size number of cells at P6 (<40 cells) (Fig. 5E) and expressing cell cycle regulation gene Cdkn1b (Figs. 5F, 5G), an inhibitor of cell cycle progression. Thus the melanocyte clusters' molecular features—cell cycle promoting and apoptosis inhibiting—might contribute to their unresolved residual cells in the mutant vitreous.

As previously noted, the vitreous melanocytes were morphologically similar to the choroid (Fig. 4). To systemically visualize the similarities and differences among these pigment cells, we merged our RNA-seq data with that of publicly available mouse RPE/ retinal choroid cells (GSE135167, PMID: 32196081) and projected the integrated data on a two-dimensional UMAP (Fig. 5H). C20 was geographically adjacent to the choroid pigment cells on the UMAP, whereas the C12 and C21 were farther away (Fig. 5H, pub-Mela [GSE135167-melanocytes]). All three clusters were farther from the pub-RPE than the pub-mela cluster (Fig. 5H), and Pearson correlation analysis of the gene expression profiles showed similar results (Supplementary Fig. S2), with C20 having the highest R-value of 0.88 (Figs. 5I, J). The data again confirmed that the vitreous pigment cells identified in the mutants are neural crest-derived melanocytes from systemic gene expression levels.

### The Vitreous Cell Composition Varied Between the Wild Types and Mutants

**Figure 6.**
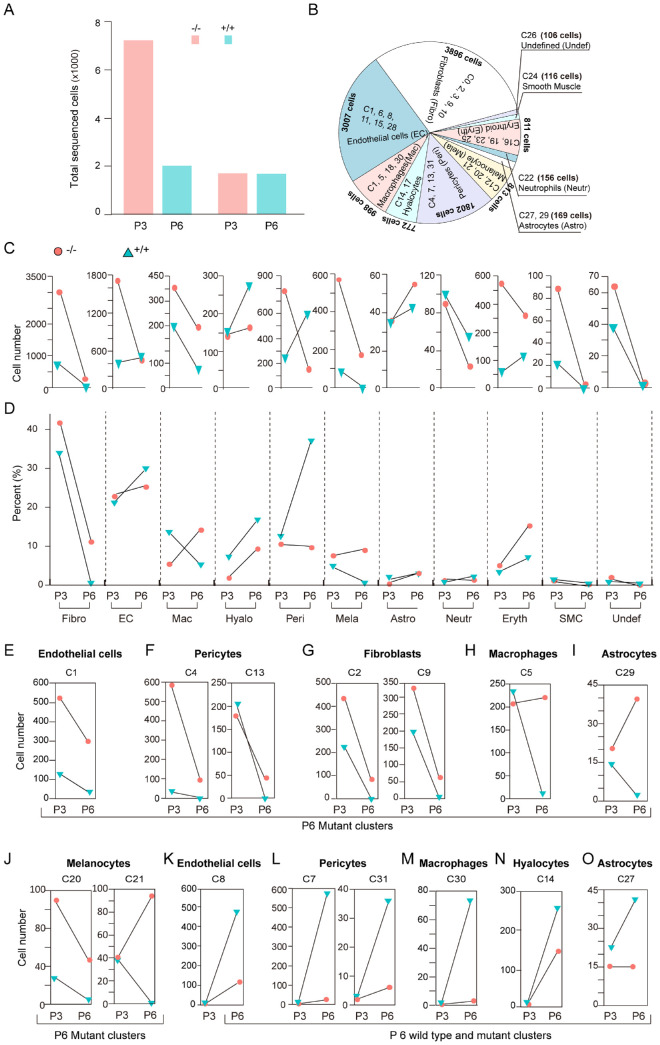
Cell composition in the wild-type and mutant vitreous. (**A**) A bar graph of total sequenced cells in the wild-type and mutant vitreous at P3 and P6 after data filtration. (**B**) Pie chart of cell fractions of the identified cell types. (**C**) Cell number of each cell type respective to genotype and age. (**D**) Cell percent of each identified cell type respective to genotype and age: The cell number of a cell type was divided by the total cells of its respective genotype and age. (**E–O**) Cell number of each cell cluster respective to genotype and age. (**E–J**) Remaining clusters in the P6 mutant but not wild-type vitreous. (**K–O**) Emerging clusters at P6 wild-type and mutant vitreous.

To grasp a full view of the HV and PFV cell types, we looked into the cell composition of the *Fz5* mutant vitreous in parallel with the wild-type controls. The sequenced cell number of the mutant P3 vitreous was considerably higher than the wild type, which declined rapidly to the similar range of the wild type at P6 (Fig. 6A). The EC, Fibro, and Peri dominated the pie chart of all types of cells identified (Fig. 6B). Surprisingly, both mutant and wild-type vitreous had the same 10 cell types at P3, among which seven cell types had larger numbers in the mutants and the remaining three showed similar sizes in both wild types and the mutants (Fig. 6C). Regardless of the big difference in cell numbers at P3, the proportions of individual cell types with respect to their genotypes are similar, with the exceptions of the Mac and Hyalo, which were apparently lower in the mutants (Fig. 6D). At P6, an additional three cell types (Peri, Mela, and Eryth) also showed apparent percentage difference (Fig. 6D). The data together suggested that the mutant vitreous was not born with novel cell types but had high cell numbers and different dynamics of cell composition per age.

Because most cells were involuted by P6, the remaining mutant cells should probably contribute to the PFV phenotypes at later ages. Detailed examination of the P6 clusters showed that endothelial cluster 1(C1) (Fig. 6E), pericyte C4 and C13 (Fig. 6F), fibroblast C2 and C9 (Fig. 6G), macrophage C5 (Fig. 6H), astrocyte C29 (Fig. 6I), and melanocyte C20 and C21 (Fig. 6J) remained in the mutant vitreous and were almost absent in the wild type (Figs. 6D–I). On the other hand, the endothelial C8, pericytes C7 and C31, macrophage C30, and hyalocyte C14 appeared to be newly developed clusters in the P6 vitreous since they were nearly absent at P3 (Figs. 6K–N). Moreover, these clusters showed higher numbers in the wild-type vitreous (Figs. 6K–N). The size of astrocyte C27 also increased and was present more in the P6 wild-type vitreous than in the mutant (Fig. 6O). The remaining clusters of the cells as mentioned above, the smooth muscle cells, neutrophils, and the undefined cells, all showed higher numbers in the mutant vitreous at P3 but either mostly degenerated or maintained in similar numbers in both wild-type and the mutant vitreous at P6 (Supplementary Figs. S3A–H). The erythrocyte clusters, although showing differences between the wild types and the mutants (Supplementary Fig. S3I), were subjectively excluded from the study because they were probably enclosed within the blood vessels and had no interactions with other cell types. Thus the mutant vitreous, starting with the same types of cells as the wild type but having larger numbers, regressed grossly normal in the postnatal development, while retaining several specific cell populations.

### Molecular Profiles of the Remaining Clusters of the Mutant Vitreous

**Figure 7.**
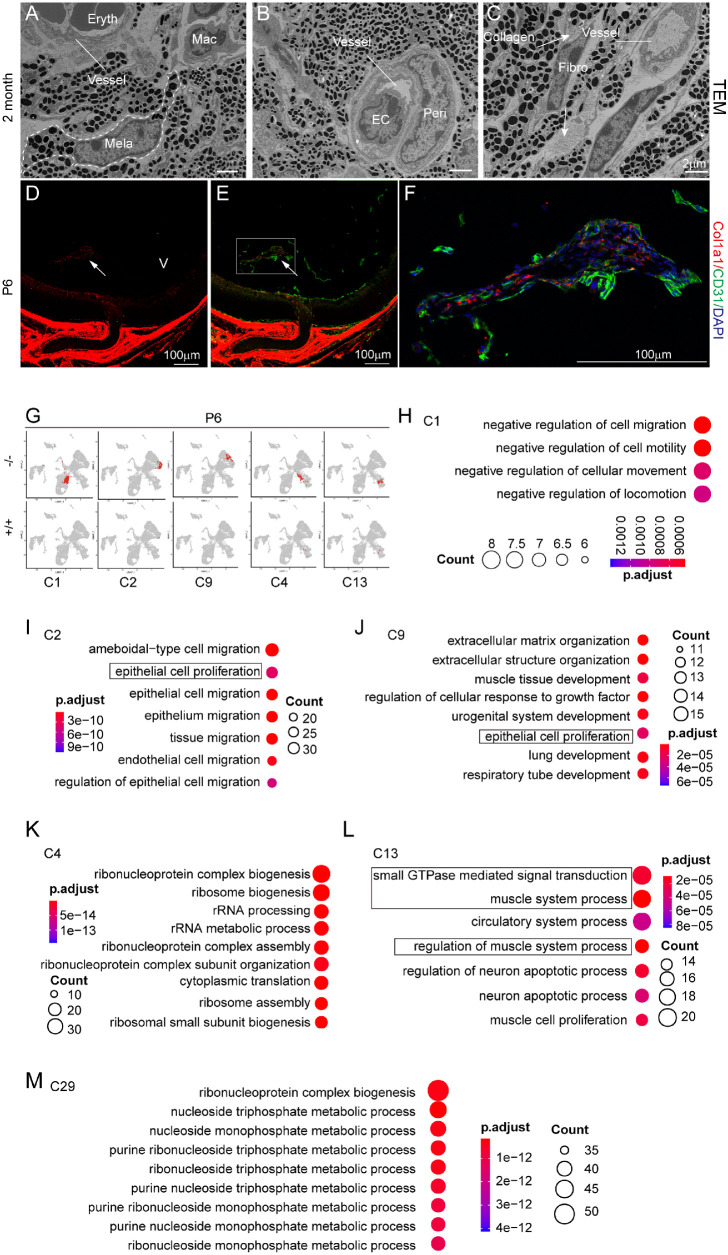
Molecular profiles of the P6 remaining clusters. (**A–C**) Transmission electron microscopy of the adult mutant pigmentary vitreous showing the existing PFV cell types. Peri, pericyte process; EC, endothelial process; Mac, macrophage; Fibro, fibroblasts; Mela, melanocytes (*dashed lines*). Vessels are indicated by short lines. Arrows point to collagen fibers. (**D–F**) Expression of Col1a1 (*red*) and CD31 (*green*) in the vitreous tissues detected by immunohistochemistry. The *arrows* in the mutant vitreous retrolental mass. The *boxed area* in (*E*) is magnified as (**F**). V, vitreous. (**G**) The remaining P6 mutant clusters on UMAPs. C1, endothelial; C2 & C9, fibroblasts; C4 & C13, pericytes. (**H**) GO terms of C1 selected from top items with *P* value < 0.001. (**I**) GO terms of C2 selected from top items with *P* value <6e-10. (**J**) GO terms of C9 selected from top items with *P* value <4e-5. (**K**) GO terms of C4 selected from top items with *P* value <5e-14. (**L**) GO terms of C13 selected from top items with *P* value <4e-5. (**M**) GO terms of C29 selected from top items with *P* value <2e-12. The boxed items highlight features consistent with cluster survival or regression. Note that “gene ratio” was left out from the GO items for conveniently viewing the data.

We next examined molecular features of P6 mutant vitreous cells, which would probably survive in the adults. We first investigated the cell types in the adult mutant vitreous using electron microscopy. Although melanocytes, endothelial cells, and pericytes were present as expected in the adult mutant vitreous (Figs. 7A–C), extracellular collagen fibers were profound near the spindle-shaped cells, indicating that these cells were likely the fibroblast (Fig. 3C). We further confirmed the existence of fibroblasts in the P6 mutant vitreous by immunostaining of Col1a1 colabeled with Cd31 (Figs. 7D, 7E). Many Col1a1-positive cells were observed intermingled with Cd31 endothelial cells in the vitreous retrolental mass (Fig. 7F). Thus all the adult PFV cell types were consistent with these of the P6 mutant vitreous.

Among all P6 mutant vitreous cells, endothelial C1 was the largest group (Fig. 7G). Functional GO analysis of this cluster showed features of negative regulation of cell motility (Fig. 7H), which might prevent it from interacting with macrophages or other cells. Both C2 and C9 fibroblasts showed the epithelial proliferation GO item, with the former leaning toward cell migration and the latter on extracellular matrix remodeling (Figs. 7I, 7J). The pericyte C4 exhibited ribosomal biogenesis and translation activities (Fig. 7K), indicating its survival strength; C13 expressed cytoskeletal small GTPases associated with cell motility and polarity (Fig. 7L), probably involved in vessel stability and remodeling. These features may partially reflect cell resilience to the vitreous regression program.

Another mutant cluster, the astrocyte C29, also increased in the mutant P6 vitreous but had a small size (Fig. 6H). These cells showed vigorous activities in translation machinery assembly and nucleotide biosynthesis (Fig. 7M), similar to melanocyte C21 (Fig. 5D), which might correlate with their enhanced migrative ability contributing to the retrolental PFV mass. Other clusters that emerged or increased in P6 wild-type vitreous (Figs. 6J–M), probably because of an altered angiogenetic environment, will not be addressed in this study.

### Phagocytic Environments Differ Between Wild-Type and The Mutant Vitreous

**Figure 8.**
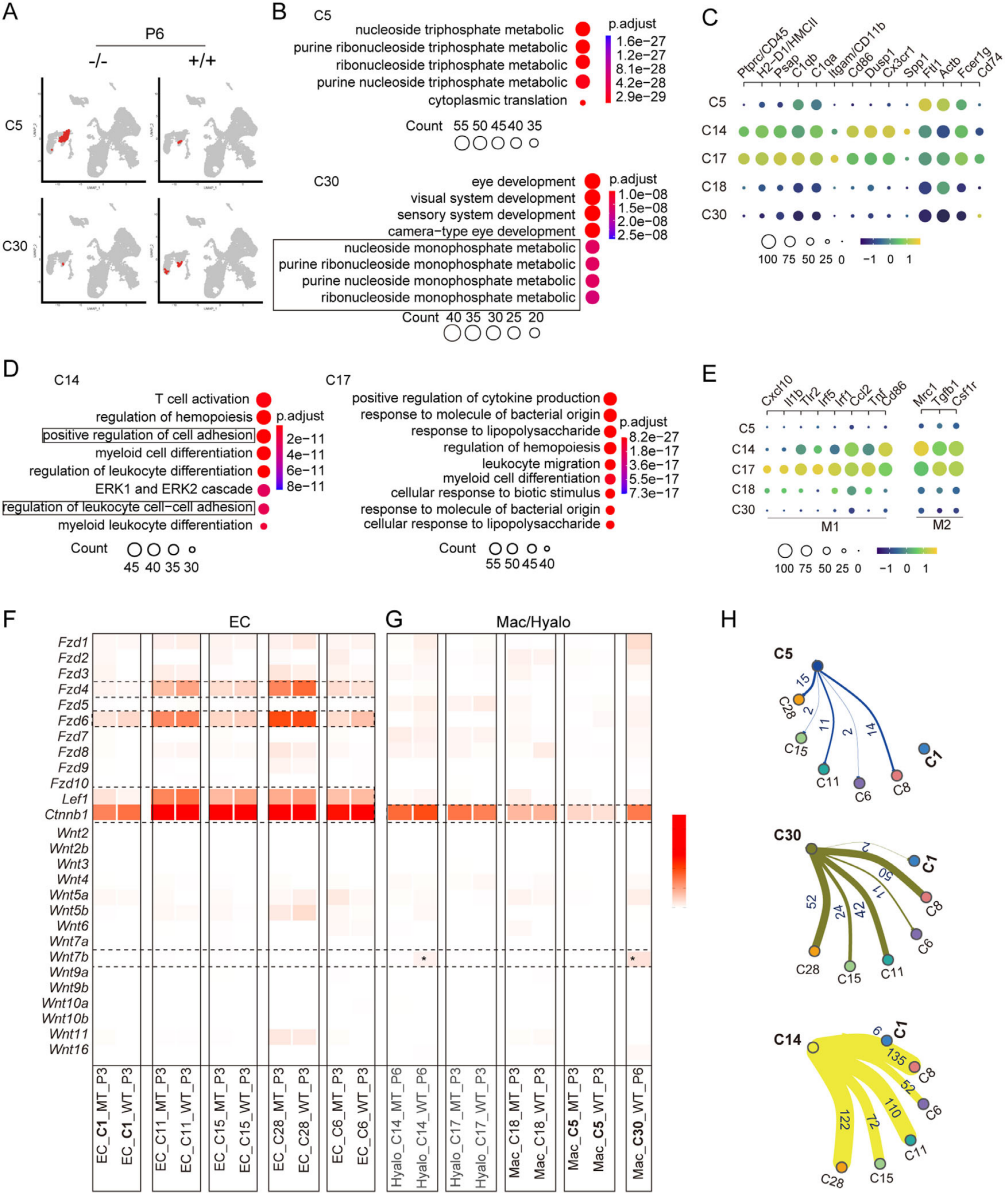
The different phagocytic environment and cell-cell interactions between the wild-type and mutant vitreous. (**A**) C5 and C30 clusters on UMAPs. (**B**) GO terms of C5 (*P* value < 8e-28) and C30 (*P* value < 2e-8). Boxed GO terms in C30 highlight the monophosphate ribonucleoside metabolic process. (**C**) Identification of the hyalocyte clusters, C14 and C17, using a set of human markers. The *color bar* indicates gene expression intensity, and the circle size indicates the cell number of a cluster. (**D**) GO terms of hyalocyte clusters C14 (*P* < 6e-11) and 17 (*P* < 8e-28). Boxed GO terms of C14 might be relevant to its phagocytic activity. (**E**) M1 and M2 marker gene expression in macrophages and hyalocyte clusters. (**F, G**) Expression of Wnts, Fzs, and Wnt canonical activity in endothelial clusters (**F**) and macrophages/hyalocytes (**G**). Note the relatively strong expression of *Fz4* and *Fz6* and canonical Wnt activity (*Lef1* & *Ctnnb1*) in all examined EC clusters except for C1. Also, note that Wnt7b (*asterisks*) was only detected in the wild-type macrophage C30 and C14 fractions. P3 was chosen for examination because a cluster's mutant and wild-type fractions can be reliably compared, because they have more cells at P3. P6 was only chosen for hyalocyte C14 and C30 because these clusters were not present in P3 vitreous. (**H**) Ligand-receptor pairs between phagocytic macrophages/hyalocytes and endothelial clusters. Note that the C1 and C5 form the least number of ligand-receptor pairs.

Macrophage phagocytosis was known to play a critical role in the HV regression.^22^^,^^23^ Macrophages are polarized into two extremes, known for pro-inflammatory M1 and phagocytic M2 states, each with unique metabolic signatures.^45^^–^^47^ C5 and C30 were the two macrophage clusters remaining in the mutant and wild-type P6 vitreous, respectively (Fig. 8A). GO term analysis showed both clusters were active in nucleoside metabolic and activity (Fig. 8B). Detail examination of their GO functional modules revealed that C5 expressed distinct genes of phosphate pentose (*Pgls* and *Taldo1*) and purine salvage pathways (*Apert* and *Hprt*), an indicator M1 state (Supplemental Data Sheet 2).^48^^,^^49^ On the other hand, C30 and expressed genes were involved in de novo purine synthesis, such as *Adss*, *Paics* and *Impdh2*, an indicator for M2 anti-inflammatory and phagocytic macrophages (Supplemental Data Sheet 2). In addition, both clusters expressed glycolysis- and electron transfer chain-related proteins (Supplemental Data Sheet 2). Furthermore, C30 expressed many crystallin genes (Go term: eye development) (Fig. 8B) known to protect against inflammation^50^^,^^51^ and also possibly from phagocytosis of the dead lens cells by C30.

In addition to macrophages, vitreal hyalocytes are macrophage-like monocyte-derived cells with phagocytic properties.^52^^–^^54^ Using a known set of human hyalocyte markers CD45, CD11b, CD86, C1qb, C1qa, and CX3cr1,^52^^,^^55^ we identified C14 and C17 (registered initially as macrophages in our scRNA-seq) to be the murine hyalocytes (Fig. 8C). By GO terms, C14 had molecular signs of T cell activation and cell adhesion, whereas C17 was relevant to the response to polysaccharide bacterial infection (Fig. 8D). Interestingly, examination of sets of markers respective to macrophage M1 and M2 states showed that both C14 and C17 expressed M1 and M2 markers, with C14 expressing relatively weaker M1 than M2 genes (Fig. 8E). Thus the lack of C30 and the smaller number of C14, both of which are M2-prone phagocytic macrophages, in the mutant vitreous may indicate a compromised phagocytic environment, contributing to the unresolved cells of the P6 mutant vitreous.

### Weaker Interactions of Macrophage/Hyalocyte and Endothelial Cells in the Mutant Vitreous

The vitreous macrophages are reported to induce HV endothelial cell death through Fz4(endothelial cell) /Wnt7b (macrophage)-mediated canonical pathway.^22^ Thus we first examined the expression of Wnt and Fz proteins in the P3 endothelial cells, which underwent nearly complete regression at P6, except for C1 of the mutant vitreous (Fig. 6E, Supplementary Fig. S3A). Among all the examined clusters, C1 had the least expression of *Wnts*, *Fzs*, and canonical Wnt activity compared with the others (Fig. 8F). *Fz4* and *Fz6* were detected in all endothelial clusters, but with weakest expression in C1 (Fig. 8F). Expression of Wnts were rather weak in all endothelial clusters, except for the mild expression being observed in C28 having fewer than thirty cells (Fig. 8F, Supplementary Fig. S3). There were no essential differences in the expression of Wnt signaling components between wild-type and mutant fractions (Fig. 8F). On the other hand, the macrophage and hyalocyte clusters were generally weak for both Wnts and Fzs expression, with the C5 being the weakest (Fig. 8G). Interestingly, only the C30 (only present in the wild-type vitreous) and the wild-type C14 fraction showed detectable *Wnt7b* expression (Fig. 8G). We further examined all ligand-receptor pairs between macrophage and hyalocyte clusters and endothelial cells and found that C5 formed no ligand/receptor pairs with C1, and C1 had the least ligand/receptor pairs with any of the P6 remaining phagocytic clusters (Fig. 8H). Therefore the weak or no expression of Wnt7b/Fz4 and other ligand/receptor pairs between the mutant macrophages/hyalocytes and endothelial cells may alleviate macrophage-induced cell death, contributing to PFV formation.

Non-canonical Wnt5a and Wnt11 in myeloid cells regulate retinal angiogenesis through VEGF inhibitor Flt.^31^ Noting that the two Wnts were expressed in several EC clusters (Fig. 8), we examined other non-canonical Wnt signaling components in the EC and Macs/Hyalo clusters (Supplementary Fig. S4). Strong expression of non-canonical Wnt effectors *Rhoa* and *Cdc42* were detected in all EC and Mac/Hylo clusters. Flt1 was also strongly expressed in ECs without significant differences between wild type and the mutants. Notably, Flt1 expression was stronger in wild-type Hyalo C14 and Mac C30 (Supplementary Fig. S4), coinciding with the stronger Wnt7b expression in the two clusters (Fig. 8G), which might together contribute to hyaloid vessel regression. Surprisingly, we did not detect cells expressing Wnt1, a marker for neural crest progenitors. A possible explanation is that Wnt-1 expression might be shut off once the neural crest progenitors reside in the vitreous.

### Vitreous Cell Types of PFV Patients and The Neural Crest Molecular Features

**Figure 9.**
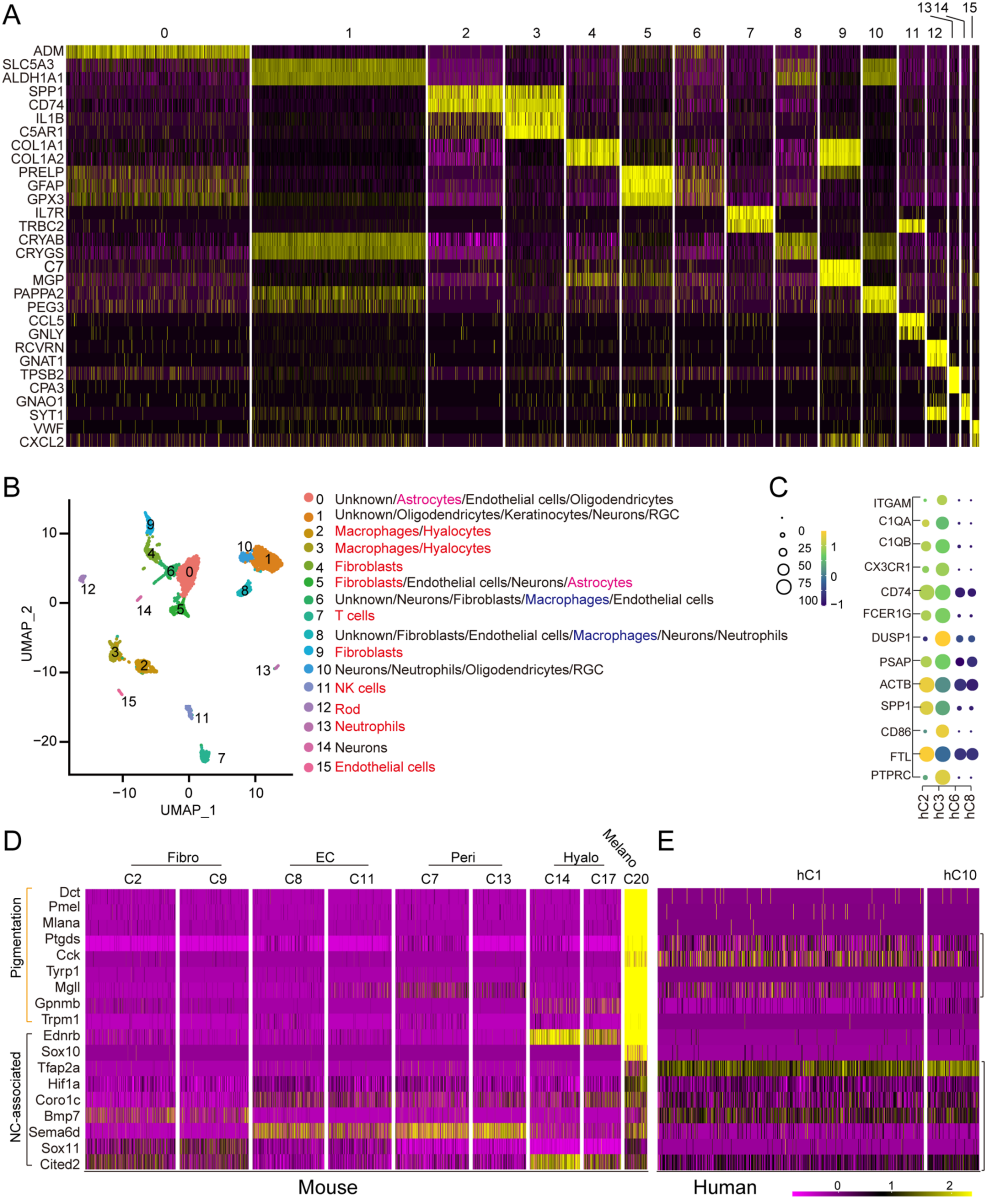
Sc-RNAseq analysis of human patient PFV samples. (**A**) A heatmap of a distinct gene set identifying cell clusters of the patient's vitreous. (**B**) UMAP projection of the identified cell clusters. Defined clusters are colored *red*. The hC6 and hC8 had some macrophage properties (*blue*). The hC0 and hC5 had some astrocyte properties (*purple*). (**C**) Hyalocyte gene expression in macrophage-related clusters hC2, hC3, hC6, and hC8. hC2 and hC3 were hyalocytes. (**D**) Expression heatmap of pigmentation and melanocyte-associated neural crest genes in mouse and human cell clusters.

To further explore whether the Fz5 mutant PFV resembles that of the human vitreous disease, we collected two human PFV/ persistent hyperplastic primary vitreous patient samples (aged 11 months and 20 days and one year and nine months, respectively) and performed sc-RNAseq analysis. The identified 15 cell clusters expressed distinct sets of genes (Fig. 9A), and their identities were defined by mining the sc-RNAseq database (Panglao sc-RNAseq DB) and the literature. Fibroblasts, endothelial cells, macrophages, rods, NK cells, T cells, and neutrophils can be readily characterized (Fig. 9B), whereas other clusters (hC0, 1, 2, 6, 8, 10, and 14) were ambiguous, having mixed features of several cell types (Fig. 9B). Using the same set of markers previously used for the mouse, we identified hyalocytes from these human clusters having macrophage properties (hC2, hC3, hC6, and hC8) (Fig. 9C) and found that hC2 and hC3 were hyalocytes (Fig. 9C). Thus several major cell types of the human PFV overlapped with the mouse PFV.

Human PFV samples do not often show pigment cells. However, the neural crest-derived mouse PFV pigment cells prompted us to examine whether human PFV cells also have melanocyte features. Hence, we examined neural crest and pigmentation markers of the mouse C20 in human and mouse clusters. A heatmap profile of single cells showed strong expression of both neural crest and pigmentation genes in the mouse C20 (Fig. 9D). Melanocyte-associated neural crest markers were also expressed in other clusters, including fibroblast C2 and C9, endothelial cell C8 and C11, pericyte C7 and C13, and hyalocyte C14 and C17 (Fig. 9D). Interestingly, the neural crest and pigment genes were also expressed relatively strong in two human PFV clusters, hC1 and hC0 (Fig. 9E). Thus the human and mouse PFV cells shared some of the neural crest and melanocyte molecular features.

## Discussion

PFV, also known as persistent hyperplastic primary vitreous, is a rare congenital condition that results from a failure of the primary vitreous and the hyaloid vasculature to regress.^56^ PFV can occur in isolated forms or be associated with other congenital disorders or syndromes, and the mechanisms of PFV formation are not well understood. Meanwhile, many genetic mouse models manifest PFV phenotypes offering great opportunities to understand the disease, among which the *Fz5* mutant PFV has not been fully characterized. In this study, we characterized vitreous cell compositions of both wild-type and *Fz5* mutant mice and human PFV samples. Through molecular marker, sc-RNAseq, and Pearson correlation analyses of the HV and PFV cells, we report a number of findings in this study: (1) a total of 10 defined and one undefined cell types were characterized in both HV and PFV of the mouse, covering all cell types combinedly reported in mice and humans, and likely represents a full spectrum of the vitreous cell types; (2) neural crest-derived melanocytes, astrocytes, and fibroblasts were specifically retained in the mutant PFV; (3) *Fz5* mutant vitreous starts with more cells and largely regressed with exceptions for a few clusters, which constitute the PFV; (4) altered phagocytic and proliferation environments and cell-cell interactions were detected in the mutant vitreous; (5) the human PFV samples shared fibroblast, endothelial, and macrophage cell types with the mouse but having distinct immune cells including T cells, NK cells, and Neutr; and last, (6) some neural crest features were also shared between certain mouse and human vitreous cell types. To our knowledge, this is the first systemic report of vitreous cell types and associated molecular features, which will pave a new avenue for molecular examination of vitreous pathologies.

The conventional view on PFV formation concerns the impairment of HV regression but rarely its formation. Accordingly, two crucial elements are mainly considered in PFV pathogenesis: the hyaloid vessel itself and the surrounding phagocytic macrophages. Altered proliferation or apoptosis of hyaloid vessel components or phagocytic activity of the macrophages often induces PFV formation, which has been shown in several elegant studies.^3^^,^^16^^,^^22^^,^^23^ On the other hand, excess vitreous cell migration through the faulty optic fissure or annular opening during development is another driver, as seen in *Neogenin*, *Ephrin-A5* and *Fz5* knockouts.^10^^–^^13^ The PFV in these mice is not conventional because the vitreous of these mice form a pigmentary retrolental mass more than hyaloid vessels. It would be clinically meaningful to understand the nature of this type of PFV and evaluate whether it would represent some of the human vitreous pathologies. The sc-RNAseq technology offers opportunities for exhaustedly looking into cell composition and associated molecular features underlying the disease pathogenesis.

The total defined ten vitreous cell types by sc-RNAseq covers all types combined in the previous studies of humans and mice. EC, Peri, Mac, Eryth, Neutr, and smooth muscle cells are vessel-related cells expected to be found in the vitreous. Fibro, Hyalo, and astrocytes are mentioned in several human vitreous studies.^57^^–^^59^ Melanocytes are often found in mouse PFV, though rarely in humans.^10^^,^^11^ A yet-to-be-defined novel cell type/cluster exists similarly in the wild-type and mutant vitreous. Interestingly, both mutant and wild-type vitreous has the same cell types and clusters at P3, regardless of the larger numbers in the former. This suggests that the HV precursors are not qualitatively altered in the mutants; rather, the lineage sizes might differ from that of the wild type. Moreover, the larger total number of cells in the mutant vitreous at P3 declines to similar levels as the wild type at P6, implying that the majority of mutant clusters regressed with exceptions for only a few constituting PFV tissue.

To understand possible mechanisms underlying PFV pathogenesis, we looked into the molecular and cellular properties of these unresolved mutant clusters. Several mechanisms could be inferred for the persistence of the PFV cells. First, the unrestricted vitreous migration of the melanocyte precursors/neural crests (through the *Fz5* defective optic fissure/disk during development) exceeds the disposal capacity of the HV regression program. This is reflected by melanocyte C20, which remarkably decreased from P3 to P6 but can't be resolved entirely (Fig. 6J). A similar rationale may apply to several other clusters, including endothelial cluster C1 and fibroblast C2 and C9, which greatly decreased in the mutant vitreous (like C20) at P6, but still remaining considerable sizes. Second, increased cell proliferation or anti-apoptotic activity in certain clusters may override the HV regression program. For example, melanocyte C21 and macrophage C5 having similar sizes in the wild-type and mutant vitreous at P3 increased in the mutants whereas declined in the wild types at P6 (Figs. 6H, 6J). The opposite changing dynamics of the two clusters might reflect different vitreous microenvironments or cell-cell interactions in the mutants and wild types. Last, the cluster dynamics also depend on intrinsic property, as exemplified by the largest mutant fibroblast cluster C0, which is involuted completely at P6 in contrast t the C2 or C9.

The macrophages and hyalocytes are critical for vitreous vessel regression.^52^^–^^54^ Because hyalocyte has not been characterized in mice, we first identified two hyalocyte clusters according to a set of markers for humans.^59^ Although the size and dynamics of one of the hyalocyte clusters are similar between the wild-type and the mutant vitreous, C14 size is smaller in the mutant vitreous. C14 is a more phagocytic- and anti-inflammatory- M2 type by marker analysis^60^ and its smaller size in the mutants may reflect a lower phagocytosis capacity. The wild-type–specific macrophage C30 also leans toward M2-type, whereas the mutant C5 leans more toward M1 based on their metabolic signatures.^48^^,^^49^ Therefore weaker M2-related phagocytic activity in the mutant vitreous might contribute to the unresolved cells.

Although it is unclear whether macrophages or hyalocytes phagocytose melanocytes or fibroblasts in the vitreous, their interactions with endothelial cells mediated by the Wnt7b/Fz4 ligand-receptor pair induce HV apoptosis.^22^ The least expression of *Fz4* receptor and canonical Wnt activity in the mutant endothelial C1 and undetectable Wnt7b expression in the mutant macrophage C5 is consistent with the fact that C1 does not involute completely in the mutant vitreous. On the other hand, Wnt7b expression in the C30 and C14 also indicates more vigorous phagocytic activity, consistent with the efficient vessel regression in wild-type vitreous. In addition, non-canonical Wnts in myeloid cells were found to regulate retinal angiogenesis through VEGF inhibitor Flt.^31^ The strong expression of *Rhoa* and *Cdc42* in all EC and Mac/Hylo clusters supports the existence of non-canonical Wnt activities. Meanwhile, Flt1 expression was stronger in the two clusters expressing Wnt7b, suggesting they may coordinate during vessel regression. Furthermore, C1 and C5 form the least reciprocal ligand-receptor pairs with other macrophage and EC clusters, which might also attenuate cell-cell interaction-induced apoptosis or phagocytosis, contributing to their survival during HV regression. Last, the ectopic vitreous astrocytes (likely from aberrant migration of retinal or optic nerve astrocytes) ensheathing the mutant hyaloid vessels may further prevent cells from interacting with macrophages or hyalocytes, thus regression. This has also been observed in *Large* mutant mice.^14^^,^^24^

The human PFV is rarely pigmented but occasionally manifests aberrant neural crest migration.^61^ We sequenced the single-cell transcriptome of two human patients diagnosed with PFV. Endothelial cells, fibroblasts, macrophages/hyalocytes, and neutrophils were similarly identified as those in the mouse vitreous. On the other hand, many others, including the existing T cells and NK cells, were not found in the mouse vitreous of the examined ages. These cells are likely from the pathological immune response but not of native vitreous components. A plausible explanation for their absence in mice is that at the examined ages, the PFV has not caused retinal damage to the degree of immune cell infiltration. Indeed, the converted mouse ages (see Methods) of the two human specimens are older than those used in this study. Several undefined clusters have neural and glial features requiring further characterization. Notably, melanocyte-associated neural crest features are also present in several human PFV clusters, even though these clusters are not the same as the mouse. Nonetheless, to the minimal evaluations, these findings suggest that the human and mouse PFV has some degree of similarity in cell composition and molecular profiles. Further investigations will be conducted with larger human samples to address whether the two species share similar pathogenic PFV programs.

In summary, we characterized PFV cell composition and associated molecular features in the *Fz5* mutant mice and two human PFV samples. The excessively migrated vitreous cells, intrinsic molecular properties of these cells, phagocytic environment, and cell-cell interactions may together contribute to PFV pathogenesis. Human PFV shares certain cell types and molecular features with the mouse.

## Supplementary Material

Supplement 1

Supplement 2

Supplement 3
