# Supplementary material for: Single-Cell Characterization of the Frizzled 5 (Fz5) Mutant Mouse and Human Persistent Fetal Vasculature (PFV)
Source: Invest Ophthalmol Vis Sci. 2023 Mar 3;64(3):8. doi: 10.1167/iovs.64.3.8 (PMC9988703; doi:10.1167/iovs.64.3.8)
Supplement: Supplement 1 [file iovs-64-3-8_s001.pdf]

Fibroblast Cluster 0, 2, 3, 9, 10; **Dcn or Lum**
Melanocytes Cluster 12, 20, 21; **Dct**
EC Cluster 1, 6, 8, 11, 15, 28; **Egfl7**
Pericytes Cluster 4, 7, 13, 31 (pericyte/smooth muscle); **RGS5**
Smooth muscle Cluster 24; **Acta2**
Macrophage Cluster 5, 14, 17, 18, 30 (*); **Apoe**
Erythroid-like and erythroid precursor cells Cluster 16, 19, 23, 25; **Alas2**
Neutrophils: Cluster 22; **S1009**
Astrocytes: Cluster 27, 29; **Gfap**
Novel: undefined Immuno-related cells: Cluster 26; **Histh2ap**

Markers to identify the cell types are highlighted

Fibroblast

Cluster0:

Col1a1,Gm26870,Col1a2,Crym,H19,Peg3,Nfia,Lum,Rps26,Mdk,Col3a1,Ptn,Dcn,Hnrnpa1,Rbp1,Rps28,Rps19,Rpl39,Rps27,Rplp1,Gas1,Rps15a,Rpl6,Serpinf1,Fbn2,Pdcd4,Rps5,Rpl36a,Mest,Rps21,Cebpd,Rpl12,Col5a2,Rps12,Rpl10a,Rps18,Rpl36,Rpl10,Rplp2,Npm1,Rpl22l1,Rpl37,Rps8,Rpl35,Rpl38,Rpl37a,Cenpb,Cdh11,Rpl31,Rpl26,Malat1,Rps27a,Rpl32,Ptp4a1,mt-Nd4l,Rps17,Emcn,Rpl17,Rps10,Rpl5,Eef1g,Eef1b2,Eef1a1,Rpl23a,Rpl41,Polr2m,Igfbp5,Snrpg,Zcchc11,Nsa2,Rpl34,Ctsk,Rnd3,Rps23,Rpl23,Rps14,Rpl28,Rpl14,Rcn3,Prrc2c,Rpl35a,Gnas,Cyth3,Rpl4,Rpl27,Tceal9,Rpl24,Pcolce,Rps13,Nap1l1,Rpl11,Rpl18a,Rplp0,Rpl9,Rps4x,Rps20,Plpp3,Luc7l3,Oat,Rpl13,Rab34,Sec61g,Thra,Rps3a1,Gm10076,Anp32b,Rpl21,Rpl27a,Socs2,Ppp1r2,Mmp2,Rps7,Rpl18,Nr2f2,Rps25,Rpl7a,Morf4l2,Rps15,Rpl29,Sox4,Ptov1,Nudt4,Rpsa,Tshz2,Siva1,Tpm1,Cbx3,Rpl22,Pmepa1,P3h3,Tfdp2,Nfib,Gsn,Maged1,Rps24,Ptges3,Rpl3,Psip1,Rpl13a,Pitx2,Epb41l4aos,Akap12,Rps9,Spats2l,Nop58,Lrp1,Il11ra1,2810474O19Rik,

Cluster2:

Ogfrl1,Ptn,Igf1,Tfap2b,Ecm1,Dcn,Igfbp2,Epb41l2,Dkk2,Lum,Vcam1,Ndnf,Pitx2,Cdkn2a,Cdkn1a,Crlf1,Phlda3,Nrp2,Col6a1,Dpysl3,Notum,Wnt5a,1500009L16Rik,Ntm,Arl4a,3632451O06Rik,Igfbp5,Pla2g4a,Nupr1,Bgn,Col26a1,Pid1,Id3,Rasl11b,Col6a2,Abi3bp,Tcf12,Tmem132c,Plcxd3,Ccnd2,Tcf4,Cacna2d1,Calcrl,Ncam1,Cdh11,Lypd1,Zbtb20,Nid1,Id1,Nav1,Nkd2,Tbx3os1,Pde10a,Fgfr1,Edn3,Zfhx3,Islr,Msi2,Ank,Lsamp,Ctsk,Nr4a1,Mylk,Rnd3,Serpinf1,Col7a1,Mmp2,Tshz2,Egr1,Nampt,Jun,Alcam,Col3a1,Gmds,Hes1,Sdk1,Pfn2,Rerg,Col8a2,Xist,Mfap4,Pcolce,Tra2a,Hsd11b1,Enpp2,Arhgap20,Ptgs2,Ogt,Rap2b,Col4a5,Cgnl1,Itm2c,Tle4,Gxylt2,Tmem9,Laptm4a,Eif1b,Atrnl1,Atp1a2,Loxl1,Nrp1,Sostdc1,Gpc6,Srpx2,S100a6,Fjx1,Clmp,Kitl,Zfhx4,Aebp1,Col5a2,Sdc2,Hey1,Abcg5,Cox6b2,Lamb1,Emilin1,Ext1,Gpr137b,Irs2,Rest,Wnt6,Creb3l2,Tspan6,Lmx1b,Tbx2,Dach1,Sms,Med13l,Eva1b,Cdkn2b,Foxc1,Hspb1,Rbp1,Tusc3,Prrx2,Gfra2,Ier5l,Bmp3,Csad,Efnb1,Lrp1,Gria3,Hlf,Colec12,Exoc4,Nfat5,Sulf2,Ttc9,Mllt3,Mfap2,Etv4,Lama2,Scarf2,Plk2,Bzw2,Ntng1,Trp53inp1,Col4a6,Crispld2,Mdk,Lgals7,Il11ra1,Ttc19,Bax,Auts2,Malat1,Igsf3,Col12a1,Snhg18,Nkd1,Nid2,Ddit4,Tmem158,Cop1,Nav3,Cadps2,Tmem2,Bmp4,Ptp4a2,Zfp703,Twist1,Kctd1,Olfm1,B3gat2,Arrdc3,Aldh1a3,Ube2e2,Fam160b1,Map1lc3a,Ltbp3,Nr2f1,Itpripl2,Rnf149,Rcn3,Spry1,Myo6,Col23a1,Tob1,Sdc4,Wdr35,Nktr,Lmo7,Plxna4,Fbln2,Plekhh2,Zfp36l2,Dd

x50,Ncald,Jund,Lhfp,Gpx8,Iigp1,Son,Zfp618,Hunk,Sh3rf1,Trub1,Tead1,Cyr61,Dner,Kcnq1ot1,Tenm1,Rhob,Rhod,Fbn2,Tfpi,Lpl,Fance,Glis1,Lrrc8d,Cpne8,Ptprs,Lpar1,Hnrnph1,Gas6,Slco3a1,Nfix,Kmt2e,Etnk1,Nfic,Mex3b,Mmp14,Pdia6,Kdm5b,Col6a3,Cped1,Rcn2,Tspan3,Sulf1,Gsn,Crym,Lrp8,Ckap4,Rbms3,Cacna1g,Psrc1,Selenos,Pnisr,Dkk3,Tiparp,Stox2,Nisch,Sdc1,Cpq,Camk2n1,Itgav,Gm42047,Purb,Ralgps2,Slc1a3,C130021I20Rik,Dpm1,6330403K07Rik,9530068E07Rik,Casc4,Spock3,Bptf,Efna5,Fscn1,Fgd6,Cask,Capg,Dnajb9,Ankrd12,Stk40,Flrt3,Ppib,Cited2,Itgbl1,Antxr1,Nbea,Itm2a,Coq10b,Rab6a,Igfbp4,Tenm3,Mrc2,Atxn7l3b,Msrb2,Robo2,Pofut2,Kctd12b,Efnb2,Rbm39,6430573F11Rik,Rsrc1,Fam53b,Calr,Peg3,Aplp2,Srsf11,Gfra1,Rps27l,Dnm1,Gdap10,Ash1l,Fam171b,Shox2,Dhrs3,Rbfox2,Sec62,Slc22a17,Fstl1,Adgrl3,Tpcn1,Snrnp70,Tnrc6a,Araf,Ror1,Rsrp1,Csnk1g1,Calu,Yipf5,Rspo2,Nrxn1,Emx2,Sat1,Gsta4,Kdm2a,Foxn3,Csnk1g2,Stx3,Mat2a,Kit,Adamts16,Samd5,Chst1,Mgp,Rarb,Mast4,Tmed1,Enpp1,Rbbp6,Golim4,Tagln,Prrx1,Rpl22l1,Fzd2,Tbx3,Cygb,Hspa5,Fos,Rab34,Layn,Ttc3,Mxd4,Sec61a1,Uba7,Yaf2,Metrnl,App,2900097C17Rik,Brd2,Gaa,Twsg1,Setd5,Pdgfra,Rbm4b,Col5a1,Pard6g,AC160336.1,Zmat3,Lmna,Tanc1,Adgrl1,Pdgfrb,Ptges,Wnt5b,Btg2,Dact3,Chchd3,Ssr3,Klf4,Xpc,P4hb,Pabpn1,Ccng1,Bmp7,Aen,Polr2m,Pura,C1qtnf4,Gm30648,Gpatch8,Chst2,Pros1,Srpr,Bfar,Vasn,Atp7a,Grb14,Frmd4a,Bmpr1a,Maml3,Wipi1,Unc13b,Atp2b4,Igf1r,Lrp1b,Hax1,Rab11fip2,Epb41l3,Ndel1,P3h3,Eya2,Isyna1,Maged1,N4bp2,Gopc,Cdkn2aip,Ccnl2,Arf4,Commd3,Gtf2ird1,Aff4,Alkbh1,Clk1,Klhl24,Htra1,Wls,Mkln1,Atf4,Timp2,Gas1,Tmed9,Pdgfc,Prrc1,P3h1,Plpbp,Senp2,Nr4a2,Fgfr2,Sema3f,Hsp90b1,Bambi,Trps1,Spin2c,Zfp644,Ptk7,Sema6a,Dzip1,Celf2,Ttc28,Ormdl3,Glg1,Ube3a,Srrm2,Zfp280d,Ski,Dhx36,Cd248,Erlec1,Pnrc1,Gpc1,Gabbr1,Tnfrsf19,Helz,Mbtps1,Rsrc2,Ergic2,Epha2,Rtn4,Mpzl1,Wrnip1,Efemp2,Ilf2,Fst,Slc38a2,Rab27b,Zcchc11,Smad1,Frem1,Dtd1,Pdia3,Aspscr1,Rbm5,Ing1,Zranb1,Stx5a,Igf2r,Piezo2,Selenom,Adgrl2,Pbx1,Errfi1,Srrm1,Lrrk1,Srsf10,Sncaip,Fxyd1,Siva1,Gle1,1190002N15Rik,Ppig,Pias1,Rbm25,Trim44,Btg1,Ssr2,Bicc1,Tceal9,Ddhd2,Shc1,Tnrc6b,Ryk,Clk4,Tulp4,Arcn1,Phf14,Tra2b,Fndc3b,Gpr153,Heph,Zfp148,Nfia,Eif4a2,Uvssa,Sf3b3,Fubp1,Ext2,Zfp292,Crebbp,Zfp266,Lman1,Gpc3,Cdip1,Abhd14a,Alx3,Bcl7c,5031425E22Rik,Kdelr2,Bet1l,Trpc4,Snai1,Abraxas2,Scarb2,Rchy1,Nab2,Robo1,Tmem119,Col16a1,Rsf1,Ankrd11,Smim3,Cldnd1,Itfg1,Oxr1,Fbln1,Tmem181a,Fn1,Mau2,Plxdc2,Milr1,Akap8l,Myh10,Dusp6,Ing4,Tmem59,Arl1,Pard3,Fkbp9,Zfp326,Bcl2,Surf4,Slc39a7,Cuedc1,Gigyf1,Lacc1,Sox11,Txndc5,Sash1,Junb,Zufsp,Ganab,Fopnl,Sox5,Cttnbp2,

Cluster3:
Col1a2,Col1a1,Peg3,H19,Cxcl12,Plagl1,Fn1,Crym,Col3a1,Gdf10,Nfix,Cpxm1,Postn,Tgfbi,Dcn,Mdk,Cebpd,Pdgfra,Cygb,Igfbp5,Lox,Crispld1,Col6a3,Epyc,Cdh11,Col5a1,2810474O19Rik,Eln,Cped1,Kazald1,Mfap4,Meg3,Col6a1,Plpp3,Mest,Nfib,Gadd45b,Col8a1,Socs3,Lum,Rplp1,Foxd1,Emcn,Socs2,Foxp2,Lrp1,Col5a2,Col16a1,Mfap2,Mmp14,Mmp2,Kcnq1ot1,Lpar1,Gpc3,Gnas,Gas1,Irs2,Ikzf4,Serpinf1,Rbp1,Aldh1a2,Adamts1,Bicc1,Nr2f2,Fbln1,Tpm1,Rpl39,Fbn2,Ccdc80,Oat,Sfrp1,Adamts2,Rps28,Igsf3,D10Wsu102e,Boc,Cdkn1c,Clmp,Irx1,Zfp36l1,Itih5,Crispld2,Stat3,Cldn11,Grb10,Rps5,Nfia,Cpm,Six1,Ppp1r15a,Rbms3,Scube1,Fgfr1,Rps19,Col12a1,Foxc1,P3h3,Emilin1,Icam1,Lgals1,Tshz2,Capn6,Rps15a,Col6a2,Rps12,Nr4a2,Ppp1r2,Rcn3,Rpl23a,Cxcl1,Rps8,Ctsk,Itgbl1,Tcf4,Klf9,Pkdcc,Col14a1,Selenom,Rps21,Rps26,Rps27,Rpl12,Il11ra1,Fst,Rab34,Akap12,Rpl10,Ifitm2,Rpl36a,Rpl36,Slc38a2,Itga8,Rpl28,Zbtb20,Pcolce,Klf5,Cyth3,Rpl37,Rpl23,Tenm3,Xist,Lama2,Barx1,Sntb2,Rps18,Rora,H2afy2,Rps27a,Abi3bp,Rpl41,Jmjd1c,Usp2,Ttc19,Rpl18a,Hmcn1,Rpl35a,Ogn,Svep1,Ltbp3,Fstl1,Ly6h,Rpl32,Parp8,Aebp1,Gclc,Rpl10a,Rps10,Pbx1,Rpl24,Hlf,Lhfpl2,Rps15,Il6st,Rpl17,Zfhx4,Rpl18,Rpl6,Spats2l,Stk39,Snhg18,Eef1a1,Ccnd2,Ta

b2,Cthrc1,Rhou,Rbfox2,Rps7,Gas2,Cntfr,Gsn,Rpl34,Rps17,Twist1,Ankrd12,Art4,Rnf122,Epb41l3,Olfml3,Ckap4,Kdm5b,Ptn,Phldb2,Rpl38,Rpl31,Col26a1,Nktr,Rpl4,Rps20,Rpl13,Smarca2,Sobp,Rpl11,Rpl22l1,Foxc2,Pdgfrb,Il33,Rps9,Nedd4,Npm1,Rps13,Rpl14,Rplp2,Scara3,Loxl1,Nop58,Tmem132c,Ptp4a1,Eya2,Ddx50,Alpl,Rplp0,Rpl13a,Bptf,Mxra8,Zim1,C1qtnf4,Cyp26a1,Shisa2,Rpl9,Rpl22,Rps4x,Lmo4,Rpl37a,Sulf2,Colec12,Ugcg,Rpl27a,Ssbp2,Calu,Eef1b2,Hes1,Rpl15,Txndc5,Fzd2,Rpl3,Chd3,Irx2,Rps2,Col11a1,Nenf,Nrp2,Rps23,Pi15,Sox4,Adamts9,Nr4a1,Gm12216,Asxl3,Rps16,Grk3,Scarf2,Foxn3,Rps25,Tceal9,Arrdc4,Rps14,Rpsa,Rpl26,Tra2a,Rps3a1,Kcne4,Tln2,Six2,Mex3a,P4hb,Arih1,Sorcs2,Gm10076,6330403K07Rik,Rpl21,Tmem159,Hey1,Sox9,Igsf10,Ptprd,Dach1,Naca,Pdgfrl,Ldlr,Gm10073,Nkd2,Nsa2,Rpl19,Matn2,Nr2f1,Bace1,Rcn2,Ttc3,Scara5,Igfbp6,Cish,Itm2a,Maged1,Dact3,Rpl5,Ank2,Rpl8,Selenos,Ptgis,Cox6c,Rack1,Hk2,Kdelr3,Cd24a,Crebbp,Rpl7a,Ankrd11,Rpl29,Ank,Mbnl2,Rpl30,Tbx15,Pmepa1,Bhlhe40,Fam110b,Siva1,Rpl7,Fmo2,Sec61a1,Bmpr1a,Sec31a,Foxp4,Prrx2,Aox3,Kcnk2,Nell1,Ptprs,Topors,Setbp1,Pamr1,Nfil3,Rps24,Fndc1,Pofut2,Ash1l,Map1lc3a,Fndc5,Zcchc11,Bdnf,Pde10a,Mpp6,Ints6,Tnrc6c,Rarres2,Runx1,Zmiz1,Epb41l4aos,Islr,Tnrc18,Rpl35,Rrbp1,Fbn1,Pitx2,Chd2,Osmr,Tnrc6b,Col9a2,Sec61g,Rarb,Igf1r,Acot1,Per3,Sdk1,Frem1,Sncaip,Gk5,Coq10b,Thbs2,Col27a1,Igf1,Rbbp6,Loxl2,Cebpb,Eef2,Fam171b,Polr2m,Rpl27,Eef1g,Nrk,Gadd45a,Vkorc1,Vcan,Mgp,Smo,Csrp2,Ptov1,Adgra3,Piezo2,Lrig3,Thra,Fmo1,Fkbp7,Emx2,Crtap,Emb,Eif3e,Aprt,Lamb1,Ntrk3,Igf2r,Uba52,Gmds,Bcl7c,Tenm4,Hdgfl3,Tspan3,Exoc4,Lix1l,Ltbp1,Tle2,Ptprk,Atp8a1,Gsta4,Cyp51,Eprs,Snhg12,Ssr2,Thbs3,Ttc28,2900097C17Rik,Hic1,Fos,Vegfa,Tmem263,Laptm4a,Tspan11,Il17rd,Tcea3,Arf4,Srebf2,Prrc2c,Hunk,Ier5l,Timp2,

Cluster9:
Col3a1,Crym,Dcn,Igfbp5,Lum,Ogfrl1,Col1a2,Ptn,Tfap2b,Pitx2,Col1a1,Dpysl3,Col5a2,Peg3,Col6a1,Nrp2,Abi3bp,Col6a3,Calcrl,Eln,Tcf4,Serpinf1,Col26a1,Nkd2,Ctsk,Nfix,Pcdh17,Mdk,Bgn,Igf1,Gdf10,Pcolce,Cdh11,Dkk2,Mfap2,Fst,Nr4a1,Itih5,Col6a2,Tshz2,Hey1,Sostdc1,Pdgfrb,Fgfr1,Rap2b,Fbn2,Igfbp2,Mfap4,Cped1,Sdk1,Sulf2,Igsf3,Pde10a,Pdgfra,Mmp2,Ank,Tcf12,Ccnd2,Lox,Zfhx4,Il11ra1,Arl4a,Plagl1,Pid1,Nid1,Gpc3,Tenm3,Kcnq1ot1,Irs2,Tmem132c,Mest,Msi2,Col5a1,Tpm1,Id3,Col16a1,Loxl1,Nr2f1,Clmp,Aldh1a2,Col8a1,Crispld2,Fam171b,Twist1,Cdkn1a,Prrx2,Foxc1,Sox11,Fbln1,Aebp1,Mmp14,Zbtb20,Nupr1,Kazald1,Zfp36l2,Nr4a2,Mllt3,Gas1,Col8a2,Edn3,Hes1,Igfbp4,Gpc6,Lypd1,Scarf2,Ier5l,Xist,Adgra3,Colec12,Pla2g4a,Mgp,Fjx1,Lrp1,Ogt,Ncald,Nav1,Ttc19,Itgbl1,Col4a5,Nktr,Rasl11b,H19,Gmds,Mylk,Hlf,Ecm1,Sncaip,Bzw2,Spry1,Rnd3,Ankrd12,Mex3b,Tln2,Dach1,Phlda3,Gxylt2,Rnf182,P3h3,Rbms3,Emilin1,Foxn3,Rbp1,Itm2a,Nfia,Myo6,Med13l,Aldh1a3,Ckap4,Spats2l,Lhfp,Epb41l2,Fbn1,Igf1r,Rbfox2,Lpar1,Gsn,Creb3l2,Postn,Timp2,Bptf,Rcn2,Six1,Rab34,Ddx50,Tmem159,Cpne8,Cygb,Laptm4a,Igsf10,Lamb1,Kmt2e,Plxna4,Fbln2,Tnc,Cyp26a1,Agtr2,Fn1,Tra2a,Rcn3,Socs2,Atrnl1,Antxr1,Grb10,Rerg,Rora,S100a6,Kitl,Svep1,Adgrl3,Ogn,Palld,Cdkn2a,Kdm5b,Ltbp3,Id1,Fam160b1,Selenos,Lmo7,Nfib,Sec61a1,Selenom,Ntm,Cpq,Auts2,Fstl1,Heca,Ptprs,Hunk,Klf5,Pura,Calu,Tnrc6c,Prrc1,Exoc4,Tle1,C1qtnf7,Flrt3,2900097C17Rik,Robo1,Mxra8,Aplp2,Itga8,Gas6,Lmna,Bcl2,Smad7,Dact3,Nr2f2,H2afy2,Eya2,Carmn,Rest,Frmd4a,Stc1,Fzd2,Cdo1,Zfp36l1,Pbx1,Rnf149,Myh10,Tox,Prdx4,Zcchc11,Kctd1,Ski,Lama2,Tead1,Col13a1,Rpl22l1,Il17rd,Tob1,Tspan3,Purb,Eif1b,Pnisr,Flrt2,Slc38a2,P4hb,Nab2,Csnk1g1,Sox4,Csad,Trp53inp1,Ndel1,Rasd1,Nbea,Vegfa,Pmepa1,Tusc3,Zfhx3,Etv4,Sel1l,Lsamp,Rarb,Rbm4b,Siva1,6330403K07Rik,Ntng1,Mat2a,Ttc3,Son,Ankrd11,Il33,Ptp4a2,P3h1,Pbrm1,Sec31a,Bhlhe40,Helz,Pgf,Frem1,Col27a1,Parp8,Epb41l3,Gtf2ird1,Ifitm2,Rps19,Polr2m,Efemp2,B3gat2,Ash1l,Bmpr1a,Cacna1g,Ryk,C1qtnf4,Enpp1,Kdelr3,Apod,Zmat3,Rbm39,Zim1,

Tceal9,Ptgis,Eif3e,Crebbp,Pdia6,Tab2,Rab27b,Islr,Atxn7l3b,Twsg1,Ssbp3,Col12a1,Ube2e2,Zfp354c,Bcl7c,Gfra2,Hsd11b1,Efnb1,Lrrk1,Id2,Steap1,Bbx,Rbbp6,D10Wsu102e,Fosb,Tbx3,Nfat5,Actn1,Pofut2,Sh3d19,Adgrl1,Itpr1,Tnrc6a,Casc4,Bicc1,Sik1,Gpr137b,Smarca2,Nfic,Fbln5,Cop1,Cpxm1,Plekhh2,Lix1l,Glg1,Steap2,Rbm25,Adgrl2,Nfatc4,Sh3rf1,Efnb2,Col23a1,Rpl12,Wipi1,Trps1,Zfp618,Vasn,Klhdc2,Cask,Smo,Eif3h,Grk3,Hdgfl3,Col4a6,Ints6,Map1lc3a,Ppib,Tiparp,Emx2,Ttc28,Lmo4,Ptp4a1,3632451O06Rik,Boc,Olfm1,Mbtps1,Ccnl2,Kcnk2,Bnip3l,Prrx1,Abcg5,Dock10,Meg3,Tnrc6b,Zmiz1,Oaf,Slc22a17,BC005561,Fkbp9,Coq10b,Lpin2,Brd3,Rpl10a,Tmem2,Malat1,Slc5a3,Stox2,Hmcn1,Kdm2a,Ptgs2,Mysm1,Arid4b,N4bp2,Dnm1,Pfn2,Mex3a,Plk2,Podxl2,Fras1,Tfpi,Yipf5,Dhx36,Ppig,Gnas,Igf2r,Tmcc1,Arf4,Jund,Dner,Setbp1,Nenf,Fxyd6,Sdc1,Cyth3,Stk40,Hdlbp,Nav2,Brd1,Neo1,Fbxo11,Arhgef40,Dchs1,Rnf122,Dzip1,Cadps2,Scara3,Ugcg,Ext1,Sulf1,Gpatch8,Hnrnph1,Gpr153,Ddr2,Scube1,Pdia3,Sntb2,Rps15a,Srpr,Cgnl1,Aff4,Vgll4,Fkbp7,Tcf7l1,Eprs,Maged1,Zranb1,Bmp3,Cdh6,Stx3,Adamts2,Arrdc3,Phf3,Chd3,Ccdc68,Gfpt1,Eva1b,Zfp148,Rps8,Sparc,Acbd3,Uvssa,Chd2,Eef1a1,Ntrk2,Rictor,Nbl1,Fndc3b,Trub1,Tle4,Setd5,Phip,Ndnf,Zfp704,Ccdc80,Cthrc1,Fam110b,Prkab2,

Cluster10
Igfbp2,Ogfrl1,Dcn,Ptn,Lum,Cdkn2a,Tfap2b,Dkk2,Phlda3,1500009L16Rik,Dpysl3,Vcam1,Pla2g4a,Igf1,Pitx2,Ecm1,Cdkn1a,Col26a1,Epb41l2,Ctsk,Col3a1,Col6a2,Rasl11b,Abi3bp,Col6a1,Igfbp5,Col1a1,Arl4a,Bgn,Nkd2,Hes1,Rnd3,Crym,Id3,Serpinf1,Ccnd2,Nfia,Msi2,Nupr1,Rap2b,Pid1,Ncam1,Mdk,Nrp2,Pcolce,Cdh11,Calcrl,Sdc2,Rbp1,Xist,Laptm4a,Mfap4,Malat1,Col1a2,Mfap2,Tcf4,Nav1,Rps26,Nfat5,Zfhx3,Tceal9,Lhfp,mtNd4l,Ccnl2,Pabpn1,Rpl22l1,Son,Gigyf1,Rab6a,Rbm39,Kdm5b,Tfpi,Gm26870,Igfbp4,Rcn2,Rcn3,Rab34,Gm26917,Med13l,Ube2e2,Fstl1,Nfic,Yaf2,Peg3,Pofut2,Hnrnph1,Cpq,Hnrnpa1,Tmem5,Srsf11,Ilf2,Tra2b,Gsn,Smad7,Safb,Ptprs,Slc39a7,Ralgps2,Dpm1,Ei24,Yipf5,Nrp1,Cox6c,Fam133b,Txndc5,Ppib,Sar1a,Spry1,Rbfox2,

Melanocytes

Cluster12
Dct,Mlana,Pmel,Tyrp1,Ptgds,Cck,Trpm1,Mgll,Gpnmb,Gstp1,Ednrb,Slc45a2,Syngr1,S100a1,Bace2,Chchd10,Kcnj13,Car4,Cyb5a,Slc24a5,Mgat4b,Tyr,Bmyc,Cd63,Atp1a1,Bhlhe41,Mt2,Cdk2,Mt1,Myo5a,Mif,H2afz,Ugp2,Vegfb,Atp6v1g1,Prdx1,Spg21,B2m,Psmd8,Selenoh,Phlda1,Aldoa,Selenop,Txn1,H3f3a,Lgals1,Atpif1,Stmn1,Uqcr11,Ndufa4,Ftl1,Cyba,Rbm3,Hnrnpa3,Uqcrq,Ndufb9,Hmgb2,Cox7c,Ppia,Cox7a2,Hint1,Dbi,Gapdh,Cox8a,Tubb5,Slc25a4,Bri3,Gm26870,Atp6v1d,Glmp,Rack1,Akr1a1,Pebp1,Banf1,Usmg5,Ndufab1,Stx12,Supt16,Vim,Svbp,Lsm4,Grpel1,Acadl,Pdcd5,Mknk2,Ybx1,Cdc40,Npm1,Lamp1,Iqgap1,Ranbp1,Atp5b,Pgk1,Fam104a,Hmgb1,Cdk4,Hspd1,Tpi1,Anp32e,Coq7,Bola2,Atp6ap2,Vps29,Nucks1,Edf1,Romo1,Lamtor2,Ndufa3,Mdh2,Smc6,Lsm5,Selenof,Ptprs,Uqcrb,Abracl,Slk,Sdhc,Ndufs5,Sdhb,Ndufa5,Wapl,Atp5g1,Pin1,Ubl5,Mdh1,Tmem160,Ndufc1,Pgam1,Cstb,Minos1,Odc1,Ubald2,Pdap1,Hsbp1,Ube2e3,Echs1,Psmb7,Sgta,Rnaseh2c,Cuta,Atp6v1a,Dst,Fuca1,Cox7b,Tmsb10,Atp5l,Fkbp4,Dctn3,Nudc,Alyref,Tmbim6,Vdac3,Fkbp2,Taf10,Ptges3,Rpl39,Tsen34,Pa2g4,Lsm2,Tcf25,Gsk3b,Grina,Atp6v0d1,Paics,Lamtor4,Hmgn2,Elf2,Gm10076,Sf3b2,Nudt21,Ndufa2,Fth1,Reep5,Ndufa12,Rps25,Cox5a,Eif3b,Samm50,Hadha,Hnrnpa1,Rpn1,Anxa2,Dnajc19,Ndufs4,Chchd1,Tmem14c,Atp6v0e,S100a10,Prelid1,Lima1,Cetn3,Rbx1,Sec11a,Ndufb6,Calm3,Psma1,Tmed10,Psmb5,Denr,Cdv3,Glrx3,Rab7,Ndufb3,Rpl36,Psmb2,Arl6ip4,Park7,Rps17,Tmpo,Rwdd1,Vdac1,Ube2m,Cacybp

Cluster20

Cck,Pmel,Dct,Mlana,Ptgds,Tyrp1,Gpnmb,Mgll,Trpm1,Ednrb,Kcnj13,Gstp1,Syngr1,Slc24a5,Bace2,Syt4,S100a1,Slc45a2,Cyb5a,Cd63,Selenop,Tyr,Chchd10,Mlph,Bmyc,Atp1a1,Car4,Cdk2,Phlda1,Myo5a,Met,Mgat4b,Tmem51,Neat1,C2,Bhlhe41,Rab38,Gyg,Atp6v1g1,Pax3,Cited1,Npnt,Oca2,Ago2,Fmn1,Igsf8,Ahnak2,Mt1,Ss18l2,Vegfb,Oxct1,Hif1a,Fam174b,Hpse,Spg21,Car6,Tinagl1,Cystm1,Sytl2,Tecpr1,Mgst1,Aldoa,C4b,Eps8,Gjb2,Ugp2,Uap1l1,Vat1,Enho,Txn1,B2m,H3f3a,Mif,Psmd8,Aph1c,Insig2,Mt2,Lgals1,St3gal6,Cort,Gpr143,Fabp3,Dmxl2,Sptlc2,Trim63,S100b,Plp1,Prdx1,Art5,Emp3,Lyst,Lbh,Idh2,Mindy2,Tmem55a,Glrb,Atp6v0b,Ldhb,Dennd5b,Fxyd2,Cyba,Mitf,Psen2,Ubl3,5031439G07Rik,Tspan10,Sdcbp,Smpdl3a,Eya1,Gsta2,Gsta1,Rab27a,Pdpn,Cd44,Atp11a,Mcoln3,Uqcr11,Eci1,Ftl1,Adam10,Aebp1,Sorbs1,Ap1s2,Tmem189,Tmf1,Bst2,Socs3,Scn8a,Anxa5,Brca1,Mgat5,Sox10,Lima1,Tmem268,Pmp22,Bri3,Ahnak,Oat,Eci2,Smyd2,Ppp1r12a,Csrp1,Mpc1,Slc6a17,Plxnc1,Tpd52l1,Tfeb,Atp6v1a,Qpct,Rragd,Dhrs3,Optn,Rab5b,Bfsp2,Mme,Ugcg,Cuedc2,Fuom,Ndufa4,Rtn4,Renbp,Gapdh,Stx12,Nckap1,Atp6v0a1,Dst,Smpd1,Ptprs,Tpp1,Nrcam,Glmp,Ssu72,Gm3776,Ctnnb1,Ctsd,Gpc1,Pianp,Lamp1,Atpif1,Selenoh,Slc25a4,Plin3,Tm7sf3,Sort1,Nenf,Dpp7,Gabarap,Lta4h,Rasgrp3,Akr1a1,1110008P14Rik,Fuca1,Celf2,Aldh2,Tmem160,Car14,4931406C07Rik,Edf1,Arap2,Glul,Atp6ap2,Rexo2,Ddt,Mtch1,Lmna,Anxa2,Itga9,Ctsl,Aff3,Uqcrq,Fam53b,Gsta4,Eogt,Nav2,Slk,Wsb2,Dbi,Ndufb9,Tcf25,Id2,Acadl,1810058I24Rik,Tmem256,Arl6ip1,Grina,Dkk3,Fdps,Gpr37,Ebp,Fth1,Vim,Wbp2,Atp6v1d,Cox7a2,Paqr6,Rnh1,Ctsz,Bex4,Timm8b,Cyp2j6,Atp6v1b2,Mknk2,2010107E04Rik,Pebp1,Synpr,Nme1,Naglu,Tmem192,Ywhah,Zfyve21,Fez1,Hacd1,Myadm,Dtnbp1,Cfap54,Il10rb,Lamtor4,Hebp1,Ppia,Zfp704,Mical3,Basp1,Iqgap1,Uqcc2,Timm13,Eno1,Gpx4,Shc4,Tmed10,Cox8a,Stx7,Chpt1,Cstb,Kit,Slc4a8,Gm11837,Aig1,Daam1,Acot1,Fstl4,Dcdc2a,H2D1,Gtf2h1,Atp6v1e1,Arl2,Svbp,Lmo4,Prelid1,Stox2,Ifit3,Cuta,Cdc40,Npc2,Rell1,Hexa,Adam33,Bex3,Ccdc152,Arpc1a,Calr,Ppargc1a,Nudt16l1,Rack1,Prss12,Dclk1,Elf2,Atp5g3,Tspan7,Amdhd2,Rbm3,Ak2,Myo7a,Gm4876,Ezr,Sgcg,Osbpl1a,Uqcr10,Cpeb4,Sord,Jarid2,Ddah1,Derl1,Sh3kbp1,Scly,Hist1h2bc,Hint1,Msmo1,Spsb1,Mgat4a,Snx10,Cox5b,Pi4k2b,Coro1c,Selenok,Sec11c,Selenof,Bex1,Wapl,Plpp2,Atp5j2,Sec61b,Fbln5,Insc,Rap2b,Phyhipl,Tbx2,Coq8a,Mien1,Ppfibp2,Dstyk,St13,1810043G02Rik,Tmem176a,Rps6ka3,Rapgef4,Loxl2,Atp5b,Cdk4,Nckap5l,Reep5,Creg1,Kif21a,Smyd3,Slc48a1,Cdh13,Cox7c,Acot13,Tcn2,Fam213b,Pole4,Cited2,Dpy19l1,Vamp8,Fuca2,Gng12,Mindy3,Rbbp7,Dad1,Scpep1,Cib1,Cadm1,Ktn1,Gnb5,Jpt1,Smap1,Scamp3,Tfap2a,Tmcc3,Anxa4,Hsd17b12,Slc25a3,Arhgdia,Hint2,Snx15,Hap1,Slc7a5,Atp6v1f,Irgm1,Cep135,Sdf2l1,Atp9a,Nipa2,Cox6b1,Rhou,Tmem176b,Mpv17l2,Cntfr,Eif3b,1810065E05Rik,Ptprj,Plekha8,Tecr,Bnc2,Dcps,Mpzl1,Thsd7a,Ndufb7,Taf1d,Gpr146,Etl4,Galk1,H2afz,Hs2st1,Cox6a1,Tmbim6,Mreg,Plxna1,Gstp2,Gabarapl1,Ndrg1,Slc3a2,Tex264,Coa3,Fam104a,Mc1r,Cycs,Cdk15,Cfl2,Plekhm2,Ndufa1,Uqcrb,Mvb12a,Serf2,Hmga1,Fam213a,Cyp39a1,Vps29,Slc39a14,Nagk,Fxyd6,Pgk1,Dag1,Scrn1,Pikfyve,mtNd1,Ccdc141,Gpr137b,Ctnna1,Atp1b3,Epdr1,Ephx1,Snhg7os,Lamtor1,Lgals3bp,Iah1,Churc1,mtCo1,Rnf13,Cdc14a,Rab11fip2,Rnf7,Ndufab1,N6amt1,Itgb3bp,Bhlhe40,Ndufa13,Myo10,Nipal3,Efemp1,Asl,Hacd2,Hspe1,Pkp4,Rragc,St3gal4,Zc3h7a,Dctn3,Itgb1bp1,Commd4,Ralgps2,Psmd10,Dlc1,Ntmt1,Rabgef1,Tmem205,Rabep1,Stat3,Ndufc1,Ccdc13,Tmod1,Rbx1,Timm10b,Cers4,Xrra1,Ywhae,Mfsd5,Txnrd1,Tuba4a,Tmem14c,Gpm6a,Mrps24,Ubl5,Cd274,Abracl,Akt1,Sbno2,Ctnnbl1,Nedd4l,Plscr1,Rab4a,Pla2g12a,Ybx1,Lamtor2,Tnfrsf21,Sgpl1,Ubl7,Stat2,Nudt3,Crtc3,Tanc1,Letm1,Snx1,Tspan4,Fam49a,Ddrgk1,Ndufb4,Sema6d,Mapk6,Il3ra,Timp2,Tmem126a,Tmem56,Cdc37l1,Txndc16,Gaa,Rtn3,Plekhj1,Got1,Nedd4,Zfand5,Scand1,Arfgef3,Atp5j,Atp5g2,Gna13,Atp5d,Tmem98,Ndufb6,Gstt2,Psenen,Slc38a1,Insig1,Yif1b,Usf2,Wdfy1,Abhd5,Plagl1,Cnp

y2,Zdhhc2,Gga2,8Sep,Dusp4,Tram1,Hectd1,Pik3r3,Ptpn14,Adi1,Atp6v0d1,mtNd4,Bsg,Nubp1,Alx1,Ndufv1,Kazn,Canx,Nrbp2,Atp5o.1,Lamp2,Lgals8,Cep83,Hnrnpa3,Etfa,Ndufs5,Ppp1r37,Npm1,Plcb4,Spp1,Atp5a1,Srp14,Atp5g1,Ndufb3,Degs1,Rps27l,Slc38a10,Vps35,Pla2g2e,Spata6,Dcaf6,Usp53,Mrpl34,Gas7,Ppfia1,Cox7b,Ptprm,Hadha,Ap3m2,Taf10,Htra2,Fnip1,Atp5c1,Hspa2,Dhcr24,Vti1b,Cst6,Scrg1,Adcy2,Josd2,Igsf11,Hspd1,Ubxn1,Tbca,Tbcb,Gtf3a,Rcc2,Pet100,Tceal9,Retreg1,Wdr1,Stau2,Snhg18,Smc6,Sox11,Ogdh,Csnk1e,Swi5,Trappc3,Gas2l3,Mdh2,Mfsd1,Vdac1,Chaf1b,Ppp1r7,Atp5k,Axin2,Washc2,Nceh1,Epb41l4b,Erlin2,Ten1,Rdx,Dip2c,Slc25a13,Selenot,C77080,Lap3,Gm1673,Cox6b2,Sirt2,Npc1,Tmem251,Fkbp2,Cdh19,Tmbim4,Fam241a,Gstm2,Naxe,Ran,Cyc1,Hdac4,Cltc,Mbd2,Klhl30,Crebrf,Plpbp,Ndufb5,Cdpf1,Wbp4,Mrps21,Camk2d,Magt1,Txndc17,Tmc6,Piezo1,Ost4,Sypl,H2afj,Adh5,Chchd2,Ndufb10,Paxip1,Gkap1,Atxn10,Wls,Ndufa3,St3gal1,Naa50,Coq7,Dync2h1,Sox6,Mtmr2,Nudt9,Park7,Rnase4,C1qtnf12,Pdha1,Kcna2,Dock7,Car8,Xpnpep1,Vdac2,Golga7,Sars,Dgcr6,Gnb1,Vapa,Cyp51,Capns1,Ahcyl2,Tenm4,Hsd17b11,Dnaic1,Baz1a,Abcd3,Aes,Atp5e,Sqstm1,Pura,St6gal1,Jkamp,Pcbp4,Bscl2,Ndufb8,Tex261,Zc3hav1,Napa,Megf10,Aup1,Ndufa12,Socs6,Cox17,Sub1,Gm2115,Ddhd2,App,Plscr2,Ctdspl,Ndufv3,Tomm20,Flot1,Mrpl41,Paics,Appl2,Pygl,Hypk,Haus7,Cd72,Tmem208,Cotl1,Ppdpf,Bcl2,Ndufc2,Washc5,Tmem191c,Sec11a,Asna1,Art3,Gm10642,Pbxip1,Sema3b,Mrps36,Pnkd,Atp6ap1,Pkib,Mmp16,Cox5a,

Cluster21
Krt18,Trpm1,Pmel,Dhrs1,Chchd10,Ptgds,Clu,Pcp4,Tst,Mgll,Dct,Rlbp1,Mgst1,Mif,Aldoa,Fam129a,Slc6a15,Mlana,Spon1,Ldhb,Pfn2,Gja1,Gapdh,Pkm,Gpnmb,Mdh1,Rbp1,Sfrp1,Gsta4,Mdk,Pax6,Gm42418,Ndufa4,Atp5b,Cst3,Slc24a5,Sesn3,Uqcr11,Tpi1,Aldh2,Slc25a4,Vegfb,Atp5g1,Bex3,Atp5o.1,Ucp2,Basp1,Eno1,Gpi1,AY036118,Tyrp1,Col9a1,Tpd52,S100a1,Glul,Cox8a,Gpx4,Uqcr10,Mt1,Atp6v1g1,Atp5j,Slc25a3,Prdx1,Ftl1,Atp5a1,Atp5d,Tkt,Atp5f1,Ndufa13,Hsp90aa1,Arl6ip1,Dbi,Selenow,Pebp1,Cox4i1,Uqcrh,Atp5h,Cd63,Ppia,Taf10,Nedd4,Chchd2,Cyc1,Actg1,Ube2m,Cryab,Scp2,Tmem256,C1qbp,Prdx2,Rnf187,Eif3l,Ctsl,Ndufs5,Polr2g,Atp6v1e1,Mrpl11,Sdha,Ndufb6,Fnta,Cuedc2,Ndufa8,Smim14,Echs1,Cope,Psmd12,H2afj,Tomm5,Cops6,Pcbp1,Pfdn6,Mrpl27,Nckap1,Cox14,Pigp,H3f3a,Dgcr6,Sdhc,Sf3b5,Edf1,Ghitm,Coq7,Mrpl28,Anapc11,Prmt1,Ndufa5,Mrpl54,Snx17,Cacybp,Zmat2,Rab7,Tgfb2,Naa20,Nudt21,Psma6,Rex1bd,Mtx2,Cltb,Sqstm1,Ctdsp2,Med10,Cox5a,Chmp4b,Snrpd2,Cct2,Hadha,Psmb5,Cyb5a,Nsmce4a,BC031181,Aurkaip1,Fundc2,0610012G03Rik,Mrps14,Commd1,Anp32a,Ndufv1,Psmc2,Ybx3,Fkbp4,Dynlrb1,Trappc2l,Sf3b6,Eif1ax,Thap12,Ybx1,Vcp,Evi5,Csnk2b,Mrpl30,Ndufs8,Ctnna1,Cops5,Rcn1,Bcas2,Ndufb2,Ndufa3,Psmd1,Lin7c,Rtn3,Mrpl41,Agpat3,Prelid1,Slc25a39,1110004F10Rik,Uqcrc2,Rsl1d1,Cnpy2,Pdhb,Paics,Mlf2,Gm16286,Cpq,Mocs2,Vamp8,Pfdn2,Hsbp1,Elof1,Gatad1,Hnrnpul1,Lamtor1,Glo1,D8Ertd738e,Fam96a,Pcif1,Nudc,Ran,Nr2f6,Selenoh,Mrps24,Cox6a1,Ergic3,Psap,Psmc5,Zfand6,Capzb,mtCo3,Rab11b,1110065P20Rik,Hsd17b10,Tgoln1,Sri,Timm17a,Wls,Mrpl18,Smim10l1,Minos1,Srsf9,Fam104a,Ppp1cc,Ubxn6,Emc8,Rab21,Polr2j,Jtb,Snrpf,Hnrnpa2b1,Vps29,Siva1,Tmem147,Sars,Psmb2,Mpc1,Arhgap5,Abracl,Plgrkt,Nras,H3f3b,Uqcrc1,Psmb6,Ccnd1,Llph,Eif3c,Chic2,Ppp2r1a,Uqcc3,Fam204a,Anp32b,Clptm1l,Ahsa1,1110008F13Rik,Cltc,Pgls,Derl1,Mrpl43,mtCo2,Adrm1,Tmem234,Tm9sf2,Psmd2,mtNd1,Rad23b,Slc2a1,Hcfc1r1,Eny2,Mtch1,Tpd52l2,Fam92a,Sirt2,Gpx8,Ccdc124

Endothelial cells
Cluster1:

Gm42418,Egfl7,Slc7a5,Cd34,Ctla2a,Slc2a1,Cldn5,Tsc22d1,Col18a1,Cd200,Tmsb10,Ramp2,AY036118,Calm1,Lars2,Sat1,Bok,Slc9a3r2,Abhd2,Vim,Abcg2,Myl12b,Htra1,Eng,Snx3,Slc6a6,S100a13,Cavin3,Abhd17a,Ablim1,Ctnnb1,Epas1,Sod1,Pitpna,Csrp2,Stk25,Sptbn1,Arl2bp,Ptma,Igfbp7,Stmn1,Mxra7,Cltb,Tnfaip1,Pim3,Crybb3,Mapk3,Arf2,Kif5b,Tpm3,Anp32a,Rps6,Afdn,Ndufa8,Gng11,Ctnnbip1,Pea15a,Ralb,Evl,Map1b,Rbm8a,Gnb1,Raly,Vamp3,Foxp1,Cthrc1,Tsc22d3,Actg1,Sparc,Ctnna1,BC028528,Ets1,Atox1,Fscn1,Tpm4,Luzp1,Ybx3,Mpzl1,Prpf19,Ddah2,Rpl38,Ptp4a3,Anxa2,Ywhab,Ppdpf,Hmgb3,Dstn,Ckb,Dab2ip,Set,Bnip2,Taldo1,Actb,Tcf3,Psma7,Oaz2,Metap2,Ehd4,Csrp1,Snrpe,Sypl,Ywhaq,Arpc3,Cdk2ap1,Serbp1,Tgfb2,Dynll2,Id1,Ccnd1,Hspg2,Cmpk1,Wasf2,Cenpb,Rpl28,Timm10b,Utp3,

Cluster6:
Egfl7,Cd34,Slc2a1,Slc7a5,Ramp2,Cd93,Adgrl4,Ctla2a,Cldn5,Pecam1,Col18a1,Flt1,Cdh5,Ptprb,Cd200,Plvap,Spock2,AU021092,Esam,Lmo2,Tspan13,Kdr,Fxyd5,Abhd2,Palmd,Eng,Slc39a8,Slc3a2,Col15a1,Vwa1,Mfsd2a,Tmsb10,Sox17,Adgrf5,Tsc22d1,Cyyr1,Calm1,Msn,Tm4sf1,Bok,Sptbn1,S100a16,Ecscr,Clec14a,Fkbp1a,Sparcl1,Tie1,Rflnb,Slc9a3r2,Afap1l1,Anxa3,Foxq1,Pim3,Pglyrp1,Rasip1,Crip2,Igfbp7,C130074G19Rik,Grasp,Rgcc,Acvrl1,Hspg2,Sparc,Mmrn2,Adam15,Emp1,F11r,Grrp1,Emcn,Sgk1,Epas1,Snx3,Nostrin,S100a13,Efna1,Ddah1,Gnai2,Gpcpd1,Abcg2,Rbpms,Ccdc85b,Luzp1,Dusp3,Slc6a6,Limch1,Afdn,Srgn,Six3,Myo10,Sat1,Anxa2,Ece1,Gimap6,Gadd45g,Macf1,Hbegf,Prnp,Abhd17a,Arl2bp,Fli1,Cdh2,Htra1,Ablim1,Mpzl1,Myl12b,Rhoa,Ctnnb1,Tpm3,Actn4,Wasf2,Col4a2,Cd81,Klf2,Ets1,Actb,Pam,BC028528,Plk2,Cavin3,Rabac1,Tpm4,Cfl1,Gng11,Cyb5r3,Atox1,Ptp4a3,Foxp1,Csrp2,Gm26870,Sh3glb1,Klf4,AY036118,Gnb1,Qk,Ppic,Vim,Cthrc1,Dstn,Ptma,App,Pomp,Actg1,Timp3,Col4a1,Arglu1,Arpc3,Rpl35,Serpinh1,

Cluster8
Ctla2a,Cldn5,Slc2a1,Cdh5,Slco1a4,Col18a1,Flt1,Egfl7,Aqp1,Slc7a5,Col15a1,Cd93,Tsc22d1,Ecscr,Esam,Slc7a1,Slc3a2,Abcg2,Ramp2,Sparcl1,Ptprb,Rgcc,Foxq1,Ablim1,Fli1,AU021092,Adgrf5,Slc39a8,Abcb1a,Kdr,Nrarp,Clec14a,S100a16,Dll4,Lmo2,Pecam1,Maoa,Acer2,Csrp2,Tril,Tmem252,Rasip1,C130074G19Rik,Spock2,Cd34,Gpcpd1,Sat1,Kank3,Lef1,Crip2,Efna1,Itga6,BC028528,Tspan13,Igfbp7,Cd200,Grrp1,Sptbn1,Six3,Adgrl4,Mmrn2,Sparc,Mal,Slc39a10,Hspg2,Bambi,Gimap1,Eng,Sema6a,Vamp5,F11r,Sbno2,Calm1,Tm6sf1,Mfsd2a,Gja1,Klf2,Sox17,Tek,Vwf,Pdgfb,Sox18,Ier2,Insr,Tbx1,Tie1,Plk2,Nampt,Limch1,Rhob,Prnp,Cthrc1,Htra1,Cyyr1,Acvrl1,Dock9,Vwa1,Eogt,Tm4sf1,Emp1,Prcp,Fkbp1a,Slc40a1,Ctnnb1,Grap,Mfng,Icam2,Gja4,Rbpms,Cavin3,Cavin2,Ece1,Sorbs2,Dusp6,Slc9a3r2,Abhd17a,Notch1,Tjp1,Grp,Fryl,Junb,Foxp1,Clec2d,Cdh2,Robo4,Aplnr,Filip1,Gimap6,S1pr1,Bok,Prkch,Sec11c,Prex2,Macf1,Tnfrsf19,Lcp1,Pde8a,Sox7,Tspan5,Tdrp,Fam13c,Arhgap31,Epha4,Ushbp1,Pomp,Rapgef5,Ifitm3,Ostf1,Arpc3,Ocln,Sgms1,Mast4,Fzd6,Palmd,Git2,Bvht,Ppic,Atp1b3,Cracr2b,Rassf3,Elovl7,N4bp3,Ccdc28b,Itih5,Dlc1,Wwc2,Trove2,Bmpr2,Cyr61,Jcad,Ccdc85b,Arap3,Scarf1,Cd320,Id1,Msn,Rgs3,Synm,Afap1l1,Tspan18,Sptan1,Gch1,Myct1,Hmcn1,Tmsb10,Epas1,Sipa1,Mecom,Vim,Kif5b,Fam43a,Actg1,Gnai2,Slco2b1,Ralb,Bsg,Anxa3,Gimap5,Lxn,Nostrin,Fam167b,Cd109,Dynll1,Apbb2,Hip1,Orai1,Yes1,Dock4,Ipo11,Lrp10,2900026A02Rik,Unc5b,Stk25,Hdac7,Elk3,Slc6a6,Pitpna,Cpe,Bnip2,Zfp36,Rab12,Pcnx,Arhgap29,Plxnd1,Fyn,Ddx50,Ppp1r16b,Ctgf,Timm10b,Spns2,Cd151,Sod1,Ppp1r13b,Jag1,Cpd,Cnot6l,4931406P16Rik,Fgd5,Frmd4b,Rapgef6,Degs1,Grasp,Fbxl7,Ivns1abp,Afdn,Thsd1,Arl4a,Slco1c1,Slc50a1,Ehd4,Unc45b,Sertad1,Lmcd1,Col4a2,Sgk1,Srgn,Pqlc1,Myl12b,Scarb1,Rassf9,Myo10,Car2,Auts2,Mpzl1,Reep3,Mcf2l,Foxf2,Entpd1,Ddah1,Mapk3,2010111I01Rik,Jup,

Znrf1,Net1,Slfn5,Slc31a2,Npr3,Gm26532,Guk1,Luzp1,Cxcr4,Plod1,Utrn,Ptprm,Btg2,mt-Co1,Snx3,Tmem88,1700123O20Rik,St3gal6,Hhex,Ppfibp1,Map4k4,Tmed5,Tnfaip1,Fmnl3,Rabac1,Hbegf,Ttc28,Map3k11,Zcchc6,Slc7a8,Mkl2,Edn1,Mef2c,Tmem204,Lrrfip1,Isg15,Sema6d,Hebp1,Zfp503,Skil,Flt4,Rsu1,Srp14,Rab11a,Pir,Slc35f2,Dab2ip,Itga5,Jam2,Apold1,Cr1l,Stap2,Htra3,Wdfy1,Map4k2,Exoc5,Srgap1,Nova2,Pim3,Rflnb,Serp2,Plec,Rai14,Plekhg1,Gnb4,Arhgef7,Sypl,Cdc42ep3,Aqp11,Zeb1,Comt,Cbfa2t3,Tgfb2,Kti12,Chst15,Ptprg,Sft2d2,Exoc3l,Fosl2,Saraf,Ccm2l,Slc38a2,Fam13a,Emcn,Mcl1,Mrpl34,Tsc22d2,Arhgef1,Dusp2,Cryab,Gtf2i,Tmsb4x,Tgfbr2,Trp53,Eml1,S100a13,Ets1,Tnfsf10,Nox4,Tspo,Rps6,Cd2ap,Lrrc49,Qk,Snu13,St8sia4,Klk8,Sh3bp5,1700020I14Rik,Slc30a1,Dad1,Nt5c,Bcl6b,Pcdh1,Akap13,Ube2g1,Midn,Enc1,Cacna1a,Cdc42ep1,Pde4b,Notch4,Mtus1,Adipor1,Gm10036,Abcg1,Srgap2,Nos3,Ctnnbip1,Arl15,Ssfa2,Cd59a,Tcf4,Anxa2,Ctnna1,Caskin2,Snn,Trim47,Eif3f,Pon2,Smco4,Gclm,Cryaa,Dgkh,Lysmd2,Vat1,Pkm,Cmtm8,Tmem30a,Foxl2,Arhgef2,Hyal2,1110034G24Rik,Pea15a,Ahnak,Arl6ip5,Lsr,Lrrc8a,Adam15,Egfl8,Klhl5,Hspa12b,Pllp,Uqcc3,Nedd9,Irf1,Avl9,Col4a1,Flnb,Kbtbd11,Rasgrp3,Irf2,Jmjd6,Kctd17,Ctnnd1,Kif26a,Rexo2,Meox1,Gcnt2,She,Taldo1,Parvb,Rcan3,Cachd1,Rell1,Shroom2,Piezo1,Tcf7,Ubl3,Swap70,Nmi,Sigirr,Magi3,Arhgef15,Ccser2,Psma7,Amotl1,Tanc1,Zfp366,Heg1,Ttc14,Wasf2,Taok2,Hspa8,Ccdc85a,Sema3g,Dock6,Plekha1,Dnm3,Arl2bp,Cep68,Wscd1,Slc16a4,Lrrc58,Crybb3,Ap2b1,Aph1a,Cat,Cav1,Cav2,Polr2e,Clic1,Tfrc,Ccdc47,Trappc4,Mob2,Rpl15,Utp14a,Slc44a1,Rnf125,Efnb2,Tagln2,Foxo1,Tspan15,Tpm3,Gltp,Taf7,Zdhhc20,Prrg2,Btf3,Vasp,Npr1,Abhd2,Coa3,Cmtm6,Ripor1,Rnf7,Smad1,Klf3,Itpr2,Actn4,Pnkd,Wbp1l,Khdrbs3,Rgl2,Rasal2,Arl6ip1,Ephb4,Rras,Ankrd13a,Bst2,Spr,Dusp11,Arhgap27,Magix,Tm2d3,Faim,Sp5,Prkab1,Chst2,Tspan12,Adh1,Nxn,Tmod3,Plvap,Eif3e,Traf7,Fam181b,Gdap10,Zfp521,Nectin2,Rtp4,Lpar6,Jag2,Tbc1d23,Fscn1,Nudt14,Podxl,Bhlhe40,Cnih4,Gpsm2,Glod4,Selenop,Sox13,Crim1,Tpd52,Klf7,Kif1b,Pabpc1,Hprt,Abca3,Pcmtd1,Zdhhc9,Timp1,Gimap4,Rasa1,Cdc42bpb,Atp1a1,Lama4,Msrb2,Ablim3,Fam102a,Utp3,Peli1,Ccnyl1,G3bp1,Klhl2,Arhgef5,Glrx2,Xiap,Ophn1,Tal1,Lyn,Rhoj,Yipf3,Adamts1,Map2k3,Cox7a2l,Mtss1,Paqr7,Eef2,Cd40,Stx6,Icam1,Snx15,Gm32688,Chst7,Tjp2,Dhrs7,Rac1,Isyna1,Fzd4,Sh3bp4,Elmo1,Ube2a,Erf,Nckap5l,Ifi35,Bmf,Mbnl2,Atraid,Golgb1,Efr3b,Tpst2,Ifnar1,Nxpe4,Ehd2,Fam198b,Bik,Lamtor5,Tspan14,Armcx1,Lmbr1,

Cluster11

Egfl7,Cd34,Slc2a1,Ctla2a,Slc7a5,Col18a1,Cdh5,Flt1,Cldn5,Kdr,Ramp2,Spock2,Eng,Adgrf5,Ptprb,Slc3a2,Cd93,Vwa1,Mmrn2,Rflnb,Adgrl4,Esam,AU021092,Cd200,Ecscr,Tspan13,Col15a1,Mal,Pecam1,Hspg2,Cyyr1,Sparcl1,Sox17,Tsc22d1,Palmd,Slc39a8,Plvap,Vwf,Sptbn1,Grrp1,Fxyd5,Lmo2,Sparc,Crip2,Slc9a3r2,Igfbp3,Abcg2,Bok,Calm1,Rasip1,Fkbp1a,Igfbp7,Msn,C130074G19Rik,Anxa3,Clec14a,Afdn,S100a16,Tbx1,Acvrl1,Eogt,Gpcpd1,Tie1,Sat1,Abcb1a,Limch1,Pglyrp1,Aqp1,Robo4,Mfsd2a,Afap1l1,Gnai2,Efna1,Podxl,Anxa2,Rgcc,Epas1,Ece1,Acer2,Ccdc85b,Tmsb10,Bmpr2,Htra1,Adam15,Prex2,Foxp1,Six3,Foxq1,Col4a2,F11r,Pdgfb,Slc39a10,Hbegf,Snx3,Ptprg,Fli1,Tmem252,Srgn,Ablim1,Tek,Mfng,Ddah1,Dock9,Tm4sf1,Entpd1,Arhgap31,Emp1,Prkch,Esm1,Slc6a6,Dok4,Rbpms,Grasp,Thsd1,Luzp1,Pim3,Unc45b,Fam13a,Gimap6,Timp3,Tdrp,Prss23,Prnp,Arl2bp,Itga6,Plxnd1,Cav1,Tmem204,Abhd17a,Ivns1abp,Emcn,S1pr1,Sgms1,Myct1,Wasf2,Cd81,Kank3,Heg1,Serp2,BC028528,Nostrin,Lsr,Macf1,Myl12b,Cavin2,Col4a1,Qk,Spns2,Fzd6,Cavin3,Ptprm,Cdh2,Klk8,Wwc2,Pde8a,Ctnnb1,Pitpna,4931406P16Rik,Ctgf,Rassf9,S100a13,Kctd17,Abhd2,Icam2,Plpp1,Cd151,Unc5b,Jcad,Tjp1,Dll4,Gimap5,Dock4,Maoa,Ccm2l,Cd59a,Tspan18,Ptp4a3,Arl6ip5,Myo10,Fyn,Hdac7,Mpzl1,Vamp5,Htra3,Atox1,Enc1,Clec2d,Sptan1,Cra

cr2b,Aplnr,Cmtm8,Dusp3,Lxn,Gja4,Tpm3,Jup,Rras,Net1,Cdc42ep3,Bcl2l1,Ly6c1,Arhgap29,Stk25,Tsc22d3,Sh3glb1,Rapgef6,Tm6sf1,Kif5b,Ets1,Clic4,Trove2,Plod1,Fgd5,Ehd4,Tgfb2,Gng11,Id1,Cyb5r3,Actn4,Pon2,Mast4,Saraf,Cthrc1,Git2,Csrp2,Reep3,Rassf3,Pltp,St3gal6,Cd109,Sipa1,Bvht,Tlnrd1,Ppic,Rab11a,Mapk3,Prcp,Sgk1,Dlc1,Sec11c,Lef1,Shroom2,Arl15,Plxna2,Ostf1,Tnfaip1,Parvb,Klf2,Mob2,Srgap2,Sbno2,Insr,Rtl8a,Klhl6,Sod1,Degs1,Gna11,Scarb1,Klf3,Dab2ip,Hecw2,Arhgef1,Rcan3,Arf2,Leprot,Hmcn1,Ctnnbip1,Slc30a1,Znrf1,Filip1,Hip1,Tpst2,Tpm4,Ppfibp1,Arap3,Ramp3,Pqlc1,Adipor1,Cfl1,Cdc42bpb,Slfn5,Rhoa,Cav2,Wscd1,Zfpm1,Ly6e,Scarf1,Golm1,Arpc3,Wwtr1,Rell1,Git1,Cltb,Klf13,Rai14,Klf7,Pomp,Ppp1r13b,Zfp503,Notch1,Arhgef15,Elk3,Lcp1,Sema6a,Rapgef5,Slc40a1,Ankrd13a,Sema7a,Jam2,Bnip2,Pls3,Cbfa2t3,Tmem88,Chst15,Rab12,Mef2a,Caskin2,Jag2,Serpinh1,Stra6,Nkd1,Yes1,Gnb1,Trim16,Ralb,Swap70,Cr1l,Fam198b,Fmnl3,Tspan5,Cpd,Auts2,4921524J17Rik,Gch1,Litaf,Ushbp1,Pcnx,Kif26a,Hprt,Sh3bp5,Pak1ip1,Sypl,Flt4,Slc7a1,Ctnna1,Rock2,Adam10,Edn1,Oaz2,Fam241a,Xiap,Dock6,Fam171a2,Tacc1,Grap,Crybg3,Bambi,Tagln2,Ppp1r16b,Gimap1,Myl6,Krtcap2,Dusp2,Tubb2a,Rgs3,Ubald2,Rapgef4,Ephb4,Rasgrp3,Cmtm6,Mfhas1,Tcf7,Aqp11,Actg1,Grp,Fzd4,Tmsb4x,Srgap1,Orai1,2900026A02Rik,Pcdh1,Mxd4,Foxl2,Adgre5,Rgs12,Mef2c,Myl12a,Cnih1,Fermt2,Notch4,Rabac1,Pea15a,Ocln,Lrrc8a,Bend5,Smpdl3a,Gle1,Vim,Evl,Lrp10,Ubl3,Reep1,Exoc3l,Arf6,Ica1,Cd38,Rras2,Baiap2,Dynlt3,Cdc42ep1,Gja1,Nova2,Cd40,Plec,Arhgdia,Fam212a,Tmod3,Slco2b1,Cdipt,Azin1,Fscn1,Nedd9,App,Lmcd1,N4bp3,Foxf2,Tnfrsf19,Pitpnm2,Adcy4,Klf4,Mindy1,Raly,Epha4,Plk2,Rac1,Ick,Mtus1,Tprgl,Ybx1,Trim25,Dysf,Map3k11,Tmed5,Ccdc85a,St6galnac4,Tpgs2,Irx3,Rrbp1,Utrn,Ybx3,Dock1,Dgkh,Nrarp,Fam43a,Tiam1,She,Piezo1,Tspo,Tmem37,Klhl5,Nid2,Ecm1,Trp53i11,Prrg2,Snrk,Mkl2,Kras,Ube2g1,Adipor2,Smagp,Arhgef28,Sp5,Tspan15,Pxn,Triobp,Lims2,Palm,Psma7,Elmo1,Mecom,Lrrc58,Vamp3,Kazn,Rnpep,Pcmtd1,Ccdc12,Card19,Tspan9,Stap2,Tmed9,Inpp5k,Cd320,Smarcd1,Akap13,Fam129b,Ppdpf,Lima1,Gng5,Kcnq1,Ube2d3,Ywhaz,Bmp2k,Lrp8,Arpc1b,Plcb1,Gnb2,Clic1,Plekha1,Clptm1,Acot9,Mxra7,Arf1,Lysmd2,Ctsw,Rab11fip5,Tmbim6,Tes,2010111I01Rik,Slc50a1,Slc44a1,Sh3bgrl3,Rsu1,Gmpr,Spag9,Rhoc,Arpc2,Adgrg1,Tmem44,Itga5,Plekho1,Anp32a,Ndufa8,Sh3bp4,Ankrd33b,Pak2,Rtl8b,Zfp521,Tnk2,Ccdc88c,Kitl,Prom1,Ccny,Cds2,Arhgef2,Ahnak,Exoc5,Vat1,Aph1a,Slc31a2,Lyn,Rbms1,Plekhg1,Clec1a,Adss,Gpr146,Mrps5,Hyal2,Sh2b3,Rps6ka3,Ehd2,Asap1,Dad1,Psma6,H2-D1,Piezo2,Impdh1,Dync1h1,Ssbp4,Rpp21,Tmeff1,Dstn,Klhl2,Tanc1,Fryl,Arl6ip1,Ccnyl1,Bik,Rps11,Tcf4,Ywhab,Cpe,Apbb2,Sema6d,Actb,Cacna1a,Agfg1,Arpp19,Sox7,Map4k4,Slc35b1,Mknk2,Tmem109,Tjp2,Fmnl2,Gatsl3,Ttc28,Rhbdf1,Vps36,Ripor1,Dazap2,Sav1,Ppia,Zcchc6,Slc38a2,Ptpn12,Btbd7,Rassf8,Nos3,Crk,Rapgef1,Zdhhc20,Rbm39,Slc35f2,Nras,Ddx50,Nes,Prkar1a,Ppp1ca,Rwdd1,Icam1,Itgb1,Arhgap27,Magi3,Cavin1,Mall,Smad5,Cdk9,Timp1,

Cluster15:
Hist1h2ap,Hist1h2ae,Stmn1,Hmgb2,Cdk1,Hist1h1b,Birc5,Hist1h1e,Pclaf,Cdca8,Top2a,Ube2c,Mki67,Cd34,Slc2a1,Prc1,Cenpf,Smc2,H2afz,Plvap,Tuba1b,Tubb5,Cks2,Hist1h4d,H2afx,Pglyrp1,Tubb4b,Lmnb1,Tk1,Fxyd5,Nusap1,Egfl7,Bok,Ramp2,Cdc20,Eng,Ctla2a,Cenpa,Slc7a5,Crip2,Tmpo,Tmsb10,Tspan13,Abhd2,Kif23,Msn,Rflnb,Slc39a8,H1fx,Palmd,Adgrl4,Spock2,Tpx2,Arl6ip1,Aurkb,Ccna2,Racgap1,Col18a1,Nucks1,Cks1b,Calm1,Tubb6,Serp2,Pbk,Ran,Cdca3,Selenoh,Ube2s,Ccnb1,S100a16,Pecam1,Ccnb2,Ptma,Spc24,Snx3,Adgrf5,Tsc22d1,Tyms,Hmgb1,Kdr,Ccdc85b,Tagln2,Pimreg,Slc3a2,Dek,Slc39a10,Gmnn,Tpm4,Sptbn1,Klk8,Kif22,Incenp,Nkd1,Grrp1,Spc25,Flt1,AU021092,Hmmr,Cdh5,Cyyr1,Cd93,Fkbp1a,Slc9a3r2,Ramp3,Eogt,Afap1l1,Smc4,Cd200,Vwa1,Rasip1,Cenpe,Tpm3,Cdkn2c,Lsm2,Rrm2,Afdn,Ckap2,Tbx1,Banf1,Bub3,Anp32b,Do

k4,Atad2,Mmrn2,Myl12b,Ubald2,Dut,Rhoa,Esco2,Actb,Anp32e,Mfsd2a,Cldn5,Ddah1,Hbegf,Kif11,Anxa3,Abcg2,Arl2bp,Hspg2,Ranbp1,Jpt1,Anxa2,Sae1,Rangap1,Six3,Ecscr,C130074G19Rik,Prex2,Arf2,Gch1,Usp1,Rbm3,Sgk1,Hmgb3,Ywhah,Grasp,Hmgn2,Robo4,Topbp1,Rrm1,Gnai2,Esam,Igfbp7,Sox17,Cavin3,Bub1,Raly,Fli1,Hnrnpab,Lmo2,Lig1,Ppia,Srgn,Ptprb,Crip1,Shcbp1,Ctgf,Adam15,Tmem109,Alyref,Hdgf,H2afv,Icam2,Podxl,Tacc3,Rad51ap1,1810037I17Rik,Hint1,Tubb2a,Clspn,Cfl1,Hprt,Smc1a,Cd109,Nrm,Mad2l1,Cd81,Pim3,Calm2,Cenpw,Nasp,Ybx1,Actg1,Cdkn2d,Mis18bp1,Kctd17,Cavin2,Prdx4,Pitpna,H1f0,Jup,Pomp,Tipin,Ywhaq,Tdrp,Sat1,Plpp1,Tie1,Txn1,Nudc,Ptprg,Gpcpd1,Luzp1,Cltb,Spns2,Maoa,Ctnna1,Ece1,Mcm6,Dctpp1,Tmem37,Nme1,Foxp1,Nostrin,Plk4,Ybx3,Snrpd1,Ncapd3,Hjurp,Ccdc34,Suz12,Acvrl1,Rpp21,Ivns1abp,Clic1,Pkm,Lcorl,Ctnnb1,Tmem14c,Cox5a,Emcn,Col15a1,Kank3,Nap1l1,Mpzl1,Nsmce4a,F11r,Hmgn5,Rad21,Tuba1a,Dbf4,Hnrnpa3,Parvb,Atox1,Arpp19,Hnrnpd,Tmco1,Set,Ddx39,Srsf7,Kpnb1,Macf1,Snrpe,Mrpl33,Tlnrd1,Hist1h1c,Srsf3,Mknk2,Golm1,Icam1,Ppil1,Cracr2b,Cnih1,Actn4,Serbp1,Rpa2,Cd59a,Jam2,Aes,Bmpr2,Snrpb,Ckap5,Gng11,Rdx,Csrp1,Mrpl18,Ostc,Cdh2,Cbx3,Bcl2l1,Emp1,Gng5,Wasf2,Ostf1,Dazap1,Prdx1,Clic4,Abcb1a,Snrpa1,Sparc,Rtl8a,0610010K14Rik,Stk25,Degs1,Nes,Thsd1,Vamp3,Mcm7,Ick,Gspt1,Lsm5,Gnb1,Arf6,Supt16,St3gal6,S1pr1,Ppp1ca,Plxnd1,Rpn2,Timm50,Sh3glb1,Slc25a5,Rassf1,Snrpg,Prss23,Mfng,Impdh2,Ssna1,Cdk4,Prkch,Tjp1,Dstn,Htra1,Srsf2,Eif5a,Itga6,Prcp,Cntln,4931406P16Rik,Rbpms,Csrp2,Lbr,Pdgfb,Cox6a1,Dnajc8,Evl,Acer2,Efna1,Tsen34,Ndufa8,Abhd17a,Rassf3,Wwc2,Dtymk,Slbp,Eif2s2,Sypl,Timp1,Kitl,Psmb5,Gnb2,Gimap6,Gclm,Qk,Pea15a,Dnajc9,Yeats4,Arpc3,Hnrnpf,Ddx39b,Slc29a1,Snrpd3,Pmf1,Cav1,Pa2g4,Cpe,Hdac7,Zcrb1,Cdc37,Fyn,Anp32a,Tspan18,Lsm3,Mfhas1,Smagp,Rras,Rfc2,Gtf2h5,Rnps1,Tspan12,Anapc5,Orc6,Cyb5r3,Tspan5,Rras2,Limch1,Rwdd1,Tnfaip1,Klf2,Metap2,Alad,Rbbp4,U2af1,Krtcap2,Cav2,Lxn,Fscn1,Pon2,Rtn3,Cenpx,S100a10,Rap1a,Ssrp1,Arl6ip5,Ppdpf,S100a13,Cwc15,Lef1,Foxq1,Rac1,Snrpf,Prelid1,Cdv3,Tek,Osbpl9,Arhgdia,Tmsb4x,Ablim1,Hnrnpa0,Erh,Ncl,Gna11,Ptges3,Rgcc,Tmem204,Eif1ad,Baiap2,Nans,Git2,Capns1,Actl6a,Gadd45g,Nsmce1,Eif6,Sec11c,Arpc5l,Thrap3,Atp5g2,G3bp1,Haus4,Vdac3,Pole3,Ube2e3,Eif4h,Terf1,Manf,Gapdh,Sptan1,Myef2,Spr,Nsd2,Smarca4,Reep5,Api5,Cd151,Stag1,Dusp3,Myo10,Commd8,Pafah1b1,Sgms1,Klf3,Acadl,Psip1,Tpr,Uqcrq,Ddx1,Fam171a2,Snrpd2,Ndufs5,Snrpa,Arf1,Hsp90aa1,Taf11,Vat1,Park7,Bzw1,Dab2ip,Rab11a,Hnrnpdl,Odf2,Pls3,Plod1,Hnrnpa1,Gltp,Clec14a,Rnaseh2c,Rbbp7,Bola2,Ctcf,Nudt21,Txndc12,Glud1,Mrpl57,Kras,Txnl1,Smndc1,Tcp1,

Cluster28

Tm4sf1,Aqp1,Igfbp3,Vwf,Slc6a6,Gja4,Stmn2,Ptprb,Clec14a,Cldn5,Efna1,Egfl7,Tmem252,Mmrn2,Eln,Ltbp4,Fbln5,Edn1,Vim,Cdh5,Fbln2,Sox17,Pecam1,Flt1,Ramp2,Cd34,Prex2,Cd93,Slc9a3r2,Ly6e,Unc5b,Cd200,Podxl,Adgrf5,Col18a1,Acvrl1,Cyyr1,Gadd45g,Efnb2,S1pr1,Klf2,Fxyd5,Esam,Msn,F11r,Crip2,Ehd2,Entpd1,Acer2,Sparcl1,Mal,Grasp,Ehd4,Azin1,Tspan13,Ly6c1,Hspg2,Mast4,Epas1,Bmx,Slc2a1,Notch1,Eng,Nebl,Id1,Cthrc1,Ecscr,Fryl,Timp3,Tek,Kdr,Heg1,Jag2,Ly6a,Sgk1,Ace,Tie1,Lmo2,Plpp1,Mecom,Tsc22d1,Trim47,Sparc,Sema3g,Foxp1,Ccdc88c,Eogt,Ece1,Sat1,Sema7a,Plec,Prss23,Cav1,Dll4,Cavin2,Afdn,Pdgfb,Txnip,Clec2d,Luzp1,Lifr,Plk2,Ifitm3,Myct1,Arhgap31,4931406P16Rik,Pqlc1,Nedd9,Mgp,Cavin3,Klf4,Itm2b,Rdx,Gnai2,Grp,Rasip1,Egfl8,Jcad,Ppic,Slc45a4,Lima1,Cyb5r3,H2-D1,Gatsl3,Ptprm,Fzd6,Htra1,Grrp1,Bmpr2,Syt15,Adipor2,Lrg1,Lysmd2,Icam2,Ankrd13a,Apbb

2,Tmod3,Stom,Ssfa2,Adamts1,Mapk3,Tgfbr2,Tspo,Smpdl3a,Sox13,Arl15,Adam15,Vegfc,Cmip,Adgrg1,Fbn1,Lsr,Bcam,Dab2ip,Sbno2,Cd151,Afap1l1,Arl6ip5,Wasf2,Utrn,Ppp1r15a,Kif26a,Snrk,Srgn,Emp1,Gimap6,Slco3a1,Gkn3,Ctnnb1,Fam198b,She,Rasgrp3,Rgs3,Fgd5,Calm1,Rbpms,Uaca,Pcsk5,Cgnl1,Cbfa2t3,S100a16,Adgre5,Kitl,Ptprg,Cltb,Cd2ap,Nfib,Ldb2,Fkbp1a,Itga6,Mkl2,Tns1,Lims2,Mfng,Rfk,Ablim1,Col15a1,Prnp,Jag1,Armcx6,Adam10,Glul,Frmd4b,Spns2,Igfbp4,Six3,Nos3,Gata2,Klf13,Tjp1,App,8430408G22Rik,Mef2c,Cdc42ep3,Rab11a,Mpzl1,Macf1,Ppp1r16b,Clec1a,Rassf9,Plcb1,Palmd,Notch4,Cd59a,Dst,Ing2,Git2,Clca3a1,Swap70,Prdx4,Cmtm8,Cxcl12,Hey1,Filip1,Ccnyl1,Nr4a2,Sptbn1,Ahnak,Xist,Gimap5,Sox18,Bvht,Tpgs2,Hdac7,Socs3,Ccdc85b,Itga5,St8sia6,Kif5b,Tmed10,Sgms1,Zfp46,Ptp4a3,Card19,Rassf3,Crip1,Adgrl4,Degs1,Rb1,Lamb2,Pon2,Fzd4,Cav2,2900026A02Rik,Cracr2b,Cx3cl1,Tpm3,Cavin1,C130074G19Rik,Sav1,Tsc22d3,Vwa1,Efr3b,Rras,Abcb1a,Jam2,Arhgap29,Fam129b,Lrp10,Tbx1,Gpcpd1,Mxd4,Plxnd1,H2-Q4,Atp8b1,Arpc1b,Cpd,Dock9,Klf3,Ccm2l,Apold1,Sash1,Btbd7,Tspan18,Clic4,Abcg2,Sox7,Cd81,S100a13,Gna11,Ephb4,Bcl2l1,Ddit4,Arhgap5,Wwc2,Ppfibp1,Elk3,Tubb2a,Parvb,Cr1l,Limch1,Asap2,Clu,Cd109,Prom1,Arhgef15,Chd3,Vamp5,Arhgef1,Tspan9,Tmem100,Krtcap2,Rnf125,Rtl8a,Dusp3,Ier2,Tes,Pcdh1,Arglu1,Tacc1,Itsn2,Smad6,Kctd12b,Cd38,Thsd7a,Tacc2,Actn4,Ybx1,Gclm,St6galnac3,Slc30a1,Qk,Ccnt1,Hip1r,Agrn,Map4k3,Lmcd1,Uchl1,Znrf1,Plxna2,Nav1,Cdk11b,Tgm2,Arf2,Mcfd2,Ushbp1,Fmnl3,Plekha1,Rassf1,Arl2bp,Ccdc28b,Clic1,Ppp1r13b,Spag9,Arap3,Stc1,Pdcd4,Unc45b,Atp1b3,Ctnna1,Rapgef5,Tspan2,Klf7,Itpr3,Col4a2,Pde8a,Dgkh,Rflnb,Cast,AC160336.1,Akap13,Orai1,Tmem88,Thsd1,Tcf4,Sypl,Ralb,Git1,Rbm39,Adcy4,Iqgap1,Esm1,AU021092,Isg15,Tgfb2,Ano6,Dynlt3,Tanc1,Plekhg5,Msx1,Smarcd1,Reep3,Golim4,Anxa3,Gimap1,Cdipt,Itgb1,Arhgef2,Sh3glb1,Prkch,Tapbp,Mob2,Rapgef2,Maoa,St3gal6,Itm2c,Anxa7,Robo4,Ttll7,Osmr,Atp2a3,Ttc28,Zcchc6,Anxa2,Tmed5,Myo10,Cyb561,Adarb1,Map3k11,Tm6sf1,Tmem204,Yes1,Gja5,Ripor1,Lrrc8c,Dync1li1,Dock6,Bnip2,Tead2,Caskin2,Tmem44,Mctp1,Cyth3,Ssu2,Emp3,Tanc2,Emp2,Fbxo7,Aplp2,Rabgap1,Myl12b,Pradc1,Zmiz1,Rps6ka3,Ick,Atp13a3,Nfat5,Mall,Crispld1,Prdm16,Ccser2,Ctla2a,Fchsd2,Slc38a2,Taok2,Tnfrsf1a,Col4a1,Gnaq,Tcf15,Ptpn14,Npr3,Cdk19,Pcnx,Smad7,Sptan1,Kank3,Slc44a1,Amd1,Slc35b3,Dync1h1,Emcn,Atp1a1,Rftn1,Map4k4,Nrp1,Ywhab,Elmo1,Jade1,Pltp,Litaf,Atl2,Sh3bgrl3,Lrrc8a,Sertad2,Atox1,Leprot,Nus1,Abhd17a,Vat1,Slfn5,Lama5,Sema6a,Plod1,Nmt2,Ywhaz,Slc35e4,Cenpt,Pls3,Klf9,Gle1,Fis1,Rgs2,Ets1,Hyal2,Ptpn12,Igfbp7,Tgoln1,Ctnnbip1,Sod1,Fam181b,Ceacam1,Gcnt2,Dennd3,Tmem109,Spop,Kras,Fam171a1,Rtn4,Btbd3,Prcp,Itgb4,Mbnl1,Plscr2,Aqp11,Pdzd2,Anp32a,Pcmtd1,S100a10,Mmp2,Cttnbp2nl,Srgap1,Ktn1,Stox2,Ube2g1,Zfpm1,Arhgap27,Pnpla2,Pdia3,Map4,Serpinf1,Pik3c2b,Ocln,Wwtr1,Scarf1,Nudt4,Mef2a,Mbnl2,Vgll4,H2-K1,Osbpl9,Plpp3,Ubl3,Tmbim6,Card10,Cd9,Sec11c,Smchd1,Tox3,Fosl2,Tox2,Taf7,Ndst1,Prrg2,Oaz2,Alas1,Rabac1,Msrb3,Arid5b,Fyn,Crybg3,Snx3,Anxa11,Lrrc58,Furin,Dram2,Gsk3b,Hsd17b4,Ifngr1,Ifi203,Fes,Gpd2,Hey2,Ubr7,Spata6,Csrnp1,Arhgef28,Tspan14,Kctd10,Fam241a,Npnt,Fli1,Marveld1,Pik3ip1,Guk1,Pim3,Dock1,Cep85l,Wdr37,Cdc42bpa,Pttg1ip,Rbbp6,Picalm,Tmod2,N4bp3,Piezo1,Tet2,Krit1,Luc7l2,Zfp91,Ddah1,Ccdc85a,Rtl8b,Elf1,Arhgef12,Sos1,Arhgdia,Gnb1,Pou2f1,Ubald2,Rgcc,Sh2b3,Chd7,Ppp2r2d,Cdc42bpb,Smc1a,Diaph2,Procr,Rnf144b,Zfp148,Rrbp1,Shroom2,Foxf2,Synpo,Cab39,Rlim,Triobp,Tmed9,Vamp3,Zdhhc12,Sh3bp4,Ankrd33b,Il2rg,Foxl2,Mindy1,Mtpn,Fam13c,Rab12,Stat3,Dag1,Pmp22,Tmbim1,Nrbp1,Wipf3,Atp2b4,Fam107a,Epha4,Arhgap23,Kbtbd2,Chd8,Rit1,Cnih1,Olfml2a,Cdc42ep1,Rapgef1,Gltp,Erbin,Hip1,Mylip,Pbrm1,Hhex,Scarb1,Tmem86a,Nr4a1,Lama3,Slc44a2,Plp2,Raly,Mycbp2,Tax1bp1,Sh3bp5,Camk2d,Tmem50a,Dnajc5,Golm1,Gch1,Lfng,Lrp6,Nr3c1,Smarca2,Nisch,Jak1,Rnf215,Fam

69b,Klf6,Hlx,Clock,Plekhf2,Mysm1,Fstl1,Sel1l,Tpd52,Tnfaip1,Igf2bp2,Cds2,Syngr2,Mcam,Galnt18,Zfp292,Slc50a1,Ptpre,Rock1,Sf3b1,Nin,Tlk1,S100a4,Tmem59,Kdm5c,Rps6ka5,Pdlim7,Irf2bp2,Rexo2,Rhoa,Hnrnpa0,Ammecr1,Prex1,Mgst3,Map1lc3b,Klhl2,Flnb,Phf20l1,Gm4707,Fos,Arpc2,Rbms1,BC028528,Mcl1,Rb1cc1,Tprgl,Foxo1,Elovl5,S100a6,Setd5,Gpr146,Saraf,Isca1,Ube2d3,Rheb,Sigirr,Sipa1l2,Mmp15,Npdc1,Iffo2,Dazap2,Midn,Id2,Rgs12,Pxn,Mapre1,Capn2,Sptlc2,Strn3,Cers4,Dctn2,Tcim,Phactr4,Arfgap3,Dnm3,Magix,Upp1,Hpcal1,Wscd1,Mtus1,Rhoc,Csnk1a1,Mindy2,Cmtm6,Hnrnpf,Hmg20b,Hspa12b,Dap,Ggta1,Plcg2,Sys1,Zcchc14,Ggnbp2,Cgrrf1,Avpi1,Pkn2,Enpp4,Tagln2,Ccdc12,Impad1,Acot9,Wdr43,Fas,Pitpnb,Zfp644,Nck1,Trim25,Ptbp3,Mkln1,Traf7,Zmym5,Nfkbia,Fam212a,Myzap,Fbxw2,Pik3r3,Edem2,Ddah2,Larp1b,Elovl1,Psma7,Gars,Per1,Lxn,Fn1,Slc25a32,Rc3h1,Rab2a,Klhl6,Pum2,Plcb4,Peak1,Tox4,Psmd1,Zfp521,Inpp1,Xbp1,Nhsl2,Atp2b1,Tra2b,Armc10,Senp6,Rab14,Tmem255b,Tmc6,Rac1,Zeb1,Gabarapl1,Srsf5,Ophn1,Hist1h2bc,Eml1,Srrm2,Purb,Pafah1b2,Ctnnd1,Tcf7l2,Pdgfd,Dnajc3,Tmem128,Lpar4,Ythdf2,Ubc,Ptma,Dnajb11,Pam,Vangl1,Shroom4,Atrx,St6galnac4,Tsc2,Mcur1,Cfl1,Rnf4,Prkd2,Tsg101,Paqr7,Cd47,Nipbl,Abcc4,Elk4,Kdm6a,Pafah1b1,Usp34,Zfp280d,Ccny,Ormdl3,Arpp19,Gramd1a,Stk24,Rab6a,Slc12a7,Eif4a2,Api5,Ccdc186,Mtmr11,Gng11,Ndfip2,

Pericytes

Cluster4:

Rgs5,Ndufa4l2,Higd1b,Myl9,Gm26870,Nrep,Gucy1a1,Itga1,Cald1,Mgp,Plat,Mfge8,F2r,Mcam,Serpine2,Gjc1,Gm13889,Col4a1,Filip1l,Maged2,Pdgfrb,Ebf1,Gng11,S100a11,Crip1,Lhfp,Col4a2,Meg3,Epas1,Atp1a2,Cdkn1c,Tm4sf1,Fstl1,Arid5b,Ifitm3,Ptk2,Cryab,Csnk1e,Myl6,Itm2a,Gm42418,Cnn2,Akap12,Ccnd1,Sdc2,Zeb2,Ptpn11,Ptp4a3,Pik3r1,Slc39a1,C1qtnf6,Ptms,Actb,Rhoj,Timp3,Nr2f2,Uba2,Malat1,Tpm1,Agrn,Pitpnc1,Map1b,Tnfaip1,Phlda1,Bmp1,Thra,Igfbp7,Tln1,Lars2,Phc2,Lamc1,Anxa6,Acaa2,Imp3,Pttg1ip,Cenpb,Lmna,Vasp,Ubr5,Zfand3,Prkar1a,Lama4,Dag1,Cyb5r3,Snhg18,Rab13,Anxa1,Mef2c,Psip1,Sh3bgrl,Foxc1,Ndufa13,Mmp14,Gnb4,Ndufb2,Ppp1r12c,Cox8a,Plxdc2,Pea15a,Pam,Ddx46,Anp32b,Eid1,Efhd2,Cd81,Dnajc8,Nkd1,Flna,Selenom,Rbfox2,Dkk3,Anxa5,Rora,Celf1,Igf2r,Cbx3,Uqcrc1,Fkbp10,Lamb1,Eif3j1,Ilk,Uqcr10,Coq7,Ndufs3,Pdcd5,Ddah2,Ktn1,Ginm1,Nptn,Utrn,Mxd4,Dpysl2,Nsrp1,P3h3,Anapc13,Lrrc58,Hnrnpl,mt-Atp8,Cmtm3,Prdx5,Cox4i1,

Cluster7

Rgs5,Serpine2,Higd1b,Nrep,Ndufa4l2,Itga1,Cryab,Myl9,Acta2,4Sep,Col4a1,Gm13889,Cald1,Zic1,Gja4,Ifitm1,Ebf1,Gucy1a1,Meg3,Mfge8,Rasl11a,Hspb2,Atp1a2,Gng11,Cspg4,Tbxa2r,Mcam,Myo1b,Notch3,Rgs16,Pde5a,Vstm4,Pde4d,Pdlim1,Fabp5,Tagln,Col4a2,Parm1,Tm4sf1,Il34,Gucy1b1,Mgp,Timp3,Trpc6,Aspn,Nkd1,Maged2,Apold1,Gjc1,Uaca,Rrad,Slc12a2,Cryaa,Cfh,Igfbp7,Fermt2,Pdlim2,Ednra,Crip1,Plat,Cox4i2,Rasgrp2,Tpm2,Des,F2r,Plce1,Epas1,Chp2,Heyl,Pdgfa,Rgs4,Nbl1,Lurap1l,Kcnj8,Lhfp,Atp1b2,Zeb2,Vtn,Spon2,Hmgcs2,Pcdh18,Cpe,Ifitm3,Lama4,Phlda1,Rarres2,Prrx1,Tagln2,Dmd,Pdgfrb,Ptk2,Foxs1,Vcl,Ets1,Prdx5,Ift43,Pten,Gm14964,Anxa5,S1pr3,Pdzd2,Atp13a5,Arhgap42,Fam162b,C1qtnf6,Slc19a1,Gper1,Agrn,Gnb4,Rasl12,Cd248,Crybb3,Ggt5,Ech1,Tspan17,Cysltr2,Fads3,Hspb1,Dhx58os,Ndst3,S100a11,Cnn2,Myl6,Angptl4,Iigp1,Cdkn1c,Adap2,Enpep,Pde3a,Ppp1r14a,Ccdc80,Axl,2010111I01Rik,Crip2,Inpp4b,Mprip,Eva1b,Capg,Csnk1e,Vasp,Oaz2,Tspan12,Atp5b,Pde8b,Nr2f2,Mmp11,Rras,Rgs7bp,Slc38a11,Cobll1,Daam2,Tpm4,Tbx2,Fam20a,Nid1,Abcc9,Myh9,Sema6d,Arhgef17,Tuba1a,Cisd3,Btg2,Acaa2,Lrrc8b,Myl12a,Lamc1,Naalad2,Clip1,Rab13,Filip1l,Egflam,Afap1l2,Tnfrsf21,Art3,Postn,Rgs6,Ifi

27,Mustn1,Cavin1,mt-
Cytb,Ecm2,Stc1,Zic4,Pitpnc1,Ilk,Prkar2b,Bmp1,Tns1,Sdc2,Gadd45b,Diaph2,Cd81,Ndrg2,Bcr,Rap2a,Itga4,Anxa1,Cd151,Crygs,Tmem45a,Lmna,4930523C07Rik,Prkar1a,Slc29a1,Arhgdib,Itgb1,Pls3,Sema5a,Fam162a,Marcks,Tnfaip1,Utrn,Ifi203,Efhd2,Lrrc32,Dapl1,Ralgapa1,Tgfb1i1,Lgals1,Nes,Prkcb,Gdpd3,Anxa6,Gcnt2,Lamb1,Plxdc2,Foxq1,Rhoc,Mylk,Col9a1,Dynll1,Car2,Fstl1,Farp1,Ajuba,Loxl2,Cxcl1,Wls,Fry,Tspan15,Uba2,Isg15,Plekhh3,Eid1,Pik3cd,Serpinh1,Lims1,Klhl23,Phldb2,Pgf,Gng2,Ak3,Atp2c1,Krcc1,Cdk6,Itgb5,Dbn1,Ptp4a3,Ldha,Folr1,Endod1,mt-
Nd4,Ppic,Plekhg2,Angpt2,Zfp703,Ccdc12,Tprgl,Gja8,Cdh6,Crybb2,Gpcpd1,Tbc1d1,Crhbp,Stk39,Jag1,Selenom,Dlk1,Uchl1,Sesn3,Fos,Mndal,Ogn,mt-
Co3,Dact1,Rsu1,Snrk,Amotl1,Specc1,Rem1,9930111J21Rik2,G0s2,Pon2,Ctdspl,Ndufs7,Imp3,Flna,Slc25a4,mt-
Atp6,Etfb,Gpx8,P2ry14,Stard8,Hmgn3,Nuak1,Mdh1,Pik3r1,Hbegf,Mxra8,Tcim,Gipc1,Fam118a,Ehd2,4833422C13Rik,Plxnb2,Nrarp,Map3k20,Smim3,Alad,Cyc1,Itm2b,Atp5d,Ndufa13,Atp5o.1,Rapgef5,2700046A07Rik,Gfra2,Rflna,Mdh2,Oasl2,Nudt4,Gadd45g,Acadl,Cdc42bpa,Mark1,Junb,Klf7,Pmp22,Cox4i1,Arhgap6,Atp5c1,Tmem51,Bcam,1700020I14Rik,Ndufs2,Mgarp,Crim1,Prr5l,Tnks1bp1,Bgn,Emp2,Dkk3,Cox8a,Gamt,Ube2r2,Apbb2,Pdlim7,Psmb5,Plcl1,Pkig,Armcx3,Dlc1,Acsl1,Gm867,Tax1bp3,Ptprk,Uqcrc1,Mef2a,Ddah2,Mical2,Pcdh19,Pea15a,2310022B05Rik,Ankrd50,Id3,Colec12,Ndufb10,Tmem59,Sptbn1,Ppp1r12a,Hspa1a,Vdac2,Klhdc8b,Dennd2a,Tmem30a,Lamb2,Pop5,Ccrl2,mt-
Nd1,Nab1,Pold4,Ndufv1,Ccnd2,Ccdc68,Esam,Smarcb1,Lpp,1110065P20Rik,Nt5dc2,Akap12,Dnpep,Cd9,Htr1b,mt-Nd2,Ndfip2,Sri,Srsf9,BC028528,Loxl3,Tnfrsf19,mt-
Co2,Fhl2,Shisa4,Tbx3os1,Arhgap1,Fam110b,Snhg18,Rnf152,Nnmt,Mnd1,Ndufb9,Mical1,Rit1,Pear1,Actr3,Nbeal1,Tspan6,Slc44a2,Fam96b,Actn1,Lrp1,Limd1,Tcf12,Selenof,Btd,Arid5b,Dock10,Pth1r,Map1lc3a,Serinc3,Gnb2,Serping1,Ginm1,Adgre5,Traf4,Bcas3,Spry4,Ndrg3,Atp5g3,Slc7a2,Thap3,Jam3,Aldoa,Serpine1,Dctn2,Ccdc3,Agpat5,Grb14,BC031181,Camk1,Itpkb,Nr1h3,Atp5a1,Prdx3,Il13ra1,Calm3,Atp5f1,Adcy6,Atp7a,C1qtnf2,Hrct1,Trappc6b,Btbd3,Dag1,Pla1a,Dtx3,Esyt1,Cxxc5,Psmd8,Isyna1,Actn4,Chmp5,Dynlrb1,Tbcb,Rbpms,Fkbp10,Vim,mt-
Co1,Gem,Gpsm3,1110008F13Rik,Clcn4,Prkar1b,Atp6v0e,Aco2,Pcdh1,Btg1,Gprc5c,Zfhx3,Selenow,C1qtnf7,Polr1d,Ndufs3,Timp2,Aoc3,Capn2,Mcm6,Atraid,Ift20,Bdnf,Pck2,Sh3glb2,Arhgef7,Plin3,Trib2,Ikbip,Ptms,Slc25a5,Sox5,Pfkp,Egr1,Acyp2,Galnt16,Rheb,Arhgef6,Kctd1,Gm12216,Txn2,Arhgef25,Slc25a3,Acat1,Dgkh,Sh2b3,Ndufab1,Synm,Wipi1,Rcsd1,Nr2f6,Ubr1,R3hdml,Nostrin,Dstn,Cp,Jkamp,Cbfa2t3,Atp5h,Fat1,Epb41l1,Galk1,Capns1,Olfml2a,Cers4,Fam174a,Acads,Rundc3a,Wtip,Fabp7,Unc13c,Foxf2,Tbx18,Abca1,Tead2,Ubr5,Dapk3,Dpysl2,Usp25,Iqgap1,Anxa2,Camk2n1,Arl3,Ywhab,Foxc2,Slc9a3r2,Ndufa11,Ndufb8,Ifngr2,S100a6,Tgfb2,Tns3,Gstk1,Diablo,Efemp2,Abhd4,Hey2,Anpep,Agap1,Cycs,Setbp1,C1qtnf1,Arhgap10,Golim4,Ano1,Paip2,Emp3,Gtf2i,Suclg1,Notch1,Myo1c,Smco4,Pam16,Decr1,Coro2b,Tln1,Ehd3,Coa3,Ppp1ca,Psmc5,Eif4e3,Itpr2,Nedd4,Snapc5,Ppp1r12c,Pygm,Vdac3,Runx1t1,Trpc1,Pigp,Cryba2,Chmp2a,Ebpl,Ankrd10,Zbtb43,Ptprf,Uqcc3,Psmc4,Phc2,Ypel3,Bcl2l12,Dlgap4,Dgcr6,Cpq,Begain,Rhoj,Arid5a,Elmo2,Lbh,Csk,Pkia,Il1r1,Ube3a,Chn1,Tmem131,Sugt1,Dnajc10,Tpm1,C1qtnf12,Ttyh2,Plod2,Cnn3,Sult1a1,Src,Dbp,Pdcd10,Etfa,Ncf2,Sapcd1,Ndufs5,Tomm22,Sh3bgrl,Itpripl2,Ptprd,Cacna1c,Nck2,Timm13,Adprh,Tns2,Fam208a,Prdx2,Mrpl58,Olfml2b,Hdac1,Zfp467,Osbpl7,Slc25a25,Spry1,Gnpda2,Nub1,Lsm10,Optc,Fam210b,Rabac1,Txndc15,Adam9,Orai3,P3h3,Cfap20,Bad,Gm14005,Mrps34,Psmb6,Ccs,Syne2,Aamp,Pttg1ip,Ndufv2,Ccser2,Plekha2,Ext2,Nt5c,Adora2a,Serinc1,Vdac1,Snai1,Peak1,Heg1,Tspan7,Cers5,Mettl26,Uqcrb,Rhbdf1,Zcchc14,Sumo2,A

ebp2,Ndufb5,Smim14,Tfpi,Prdm1,Uqcrc2,Fh1,Acadvl,Cyb5r3,Lsm1,Pir,Pmpcb,Asph,Cnpy2,Tmod3,Ccdc124,Mrpl36,Top2b,Rasgrp3,Cops6,Znhit1,Rock1,S100a10,Fam13c,Slco3a1,

Cluster13

Rgs5,Itga1,Ndufa4l2,Cald1,Higd1b,Serpine2,Mfge8,Gucy1a1,Ebf1,Gm13889,F2r,Abcc9,Col4a1,Ifitm1,Pdgfrb,Meg3,Nrep,Myl9,Mgp,Plat,Gjc1,Notch3,Kcnj8,Mcam,Col4a2,Gng11,Atp1a2,Chp2,Zic1,Gja4,Lhfp,Gucy1b1,Cfh,Cspg4,Myo1b,Cox4i2,Adap2,Ednra,Maged2,Pdlim1,Pde5a,Cd248,Rgs4,Pten,Uaca,Pcdh18,Rgs16,Nbl1,Rasgrp2,Slc12a2,Ifitm3,Mprip,Arhgap42,Epas1,Art3,Hspb2,Myh9,Ncam1,Axl,Sparcl1,Atp1b2,Nid1,Prrx1,Cdkn1c,Il34,C1qtnf6,Phlda1,Stc1,Ptk2,Tm4sf1,Rasl11a,Arhgef17,Fermt2,Filip1l,Fam162b,Agrn,Arid5b,Igfbp7,Vstm4,Tns1,Foxs1,Ets1,S100a11,Parm1,Zeb2,Ggt5,Prkcb,Sdc2,Aspn,Mylk,Iigp1,Itga4,Limd1,Oaz2,Rgs7bp,Bmp1,Timp3,Cxcl1,Slc19a1,Dmd,Farp1,Gnb4,Rapgef5,Tnfaip1,Heyl,Daam2,Vcl,Lama4,Mmp11,Pik3r1,Apbb2,Cdk6,Mt3,Hspb1,Csnk1e,Tspan17,Fstl1,Fads3,Cpe,Pde3a,Ppp1r14a,Des,Loxl2,Tbxa2r,Vasp,Ptprk,Trpc6,Pde8b,Naalad2,Fam20a,Snrk,Ajuba,Plce1,Rap2a,Arhgef7,Ccdc80,Pdzd2,Gper1,Lrrc8b,Acta2,Ift43,Itgb1,Serpinh1,Rasl12,Fhl2,Ndst3,Vtn,Ifi203,Apold1,Itgb5,4931406P16Rik,Lurap1l,Tpm4,Pear1,Rhoj,Sema6d,Gm14005,Ctdspl,Arhgdib,Eva1b,Cpa1,Marcks,Bcr,Traf4,Gcnt2,Plxdc2,Ankrd50,Mxd4,Bgn,Utrn,Enpep,Lamc1,Akap12,Rarres2,Tnfrsf21,S1pr3,Cd81,Dtx3,Rcsd1,Phc2,Efhd2,Ehd3,Ralgapa1,Osmr,Gab1,Diaph2,Cobll1,Myl12a,Stard8,Slc38a11,Pde4d,Carmn,Egflam,Crip1,4833422C13Rik,Plcl1,Slc44a2,Myl6,Pitpnc1,Ak3,Lgi1,Itm2a,Eid1,Gadd45b,Flna,Gm14964,Lamb1,Tspan12,4921524J17Rik,Ccdc3,Dlc1,Prkar1a,Plxnb2,Rras,Map3k20,Mef2c,Cnn2,Ttyh2,Gm12216,Btbd3,Btg2,Atp5b,Wls,Zcchc14,Fabp5,Nab1,Imp3,Clip1,Rrad,Pla1a,Uchl1,Rab13,Capg,Atp2c1,Notch1,Rgs6,Spon2,Dag1,Fyn,Endod1,Plekhh3,Tbc1d1,H6pd,Hey2,Sptbn1,Anxa5,Mndal,Plekhg2,Cisd3,Oasl2,Fam107b,Mef2a,Tbx2,Acaa2,Prkar1b,Pik3cd,Loxl3,Zfp703,Mark1,Peak1,Hbegf,Nr2f2,2310022B05Rik,Prkar2b,Lrp1,Tnks1bp1,Klf7,Fam210b,Cdc42ep4,Tpm2,Prdx5,Sox5,Lpp,Sh3glb2,Smim3,Hspa1a,Lrrc58,Ppp1r12a,Arhgap10,Atp7a,Cox8a,Tbx3os1,Dhx58os,Hivep2,Tax1bp3,Hmgcs2,Tprgl,Itm2b,Pcdh19,Rock2,Btg1,Sema4c,Rbpms,Amotl1,9930111J21Rik2,Olfml2b,Inpp4b,Tagln2,Uqcrc1,Ndrg2,Ndufs7,Agap1,Anxa6,Epn2,Igf2r,Xpc,Sbno2,Dbn1,Tln1,Runx1t1,Epb41l1,Gpx8,Cdc42bpa,Ubc,Il1r1,Angpt2,Ptbp3,Kctd10,Tmem45a,P2ry14,Specc1,Ptpn9,Pdlim2,Gm867,Trib2,Hspa1b,Ppic,Rnf152,Dlk1,Selenom,Wwtr1,Ccdc12,Itgav,Ech1,Ilk,Nkd1,Il13ra1,Spry1,Zfp467,Trpc1,Lrrc4,4930523C07Rik,Dennd2a,Pttg1ip,Prkg1,Hrct1,Pls3,Plod2,Foxc1,Ldlrap1,Pdgfa,Dact1,P3h3,Arap2,Snhg18,Mmp14,C1qtnf1,Ogn,Actn4,Foxf2,Tmod3,mtCytb,Agpat5,Crip2,Emp2,Golim4,Mustn1,Junb,Airn,Rsbn1l,Ecm2,Ube2r2,Cers4,Ginm1,Cds2,Wtip,Sesn3,Tmem59,Gadd45g,Fkbp10,Fry,Gng2,Nub1,Afap1l2,Ndufa13,Tsc22d4,Eif4ebp2,Cerk,Phldb2,Arhgap1,Ywhaz,Cnrip1,Pold4,Tgfb1i1,Tmem86a,Zcchc24,Tcim,Fjx1,Rhbdf1,Spry4,Serinc3,Clcn4,Mxra8,Btd,mtNd5,Aaed1,Tspan15,Tns3,Pcdh1,Cryab,Ptms,Uba2,Mcrs1,Mef2d,Gfra2,Plscr1,Src,Car2,Dnpep,Colec12,Cebpd,E130308A19Rik,Itm2c,Syde1,Ppp1r12c,Sv2a,Egr1,Zic4,Ano1,Cyb5r3,Usp25,Ndufs2,Ptprd,Esam,Tuba1a,Galnt10,Klhl23,Sdc3,Ypel3,R3hdm2,Zbtb34,Ubr5,Nuak1,Zbtb43,Malat1,Adap2os,8Sep,Tspan6,Zfand3,Nf1,Cavin1,Prkacb,mtNd4,Mical1,Serping1,Dcbld1,Slc25a25,Frmd6,Lasp1,Jun,Sh2b3,Unc5b,Rflna,Lrrc32,Rbms3,Lims1,Myo18a,Pald1,Gna13,Timp2,Hipk1,Mfap3l,Ktn1,Isg15,Arhgef12,Nck2,Nt5dc2,Wipi1,Foxp1,Aoc3,Rhoc,Slc29a1,Chst2,Pon2,Bcar1,Ssr3,Dzip1l,Mrc2,Cacna2d1,Lamb2,Abhd4,Neu1,Top2b,Stx1a,Tle1,Ptp4a3,Itpr2,Lrrfip2,Nbeal1,Pea15a,Tjp1,Adcy6,Nnmt,Arhgef25,Ankrd10,Olfml2a,Rora,Osbpl7,Tmem106b,Arid5a,Tfpi,Arhgef6,Shc1,Tmem204,Ifi27,Syne2,Tspan7,Csk,Dgkh,Adora2a,Map4k4,Nr2f6,Cyth3,Pck2,Tmem136,Ehd2,Cd151,Tecpr1,Gnb2,Kank2,Crlf3,Brd4,

Hmg20b,Ppp1r18,Socs1,Foxq1,Atp5c1,Fam104a,Sh3bgrl,Ncf2,Apod,B230219D22Rik,Ltbp4,Rock1,mtNd2,9530068E07Rik,Gamt,Rit1,Leprotl1,Akap7,Kifc3,Serinc1,Krit1,Ap2s1,Cox4i1,Bod1l,Mgrn1,Dnajc3,Rhod,Dctn2,Arhgap6,Pkd2,Ccni,BC028528,Ankrd44,Begain,Camk2d,Tmem50a,Bok,Gm13470,Coro2b,Aco2,Rac1,Lin7a,Lats2,Wrnip1,Pygm,Gm26825,Pld1,Tmem259,Mnd1,Gstk1,Cirbp,Adamts4,Nedd4,Ccdc68,Acsl1,Atp5a1,Cacna1c,Atp5o.1,Itpripl2,Myo10,Picalm,Adamts1,Capn2,Cdk9,Nostrin,Eif1,Zfr,Socs3,Jak1,Adgre5,Tnfrsf1a,Itpkb,mtNd4l,Dock10,Pcbp2,Sh3pxd2b,Nckap1,Sri,Txn2,Ndfip2,Ptpn11,Ifngr2,Anxa11,Bmpr2,Peli1,Acads,Dock6,Chn1,Nxph4,Nes,M6pr,Nptn,Atp5d,Asap2,Cdk14,Csnk1g2,Ptpn1,Runx1,mtCo1,Pdcd10,Ccdc88a,Pla2r1,Fbxo32,Map7d2,Tns2,Snrnp48,Tbx18,Snx18,Gpsm3,Gucy1a2,9Sep,Rerg,Rapgef4,Sema5a,Commd8,Ptk7,Eif3c,Tmem131,Map1lc3a,Nrip1,Mgea5,Ppfibp1,Rbms1,Arpc5l,Kcne4,Capns1,Ubb,Rsu1,Tpst2,Rab11b,Ubr2,Pop5,Rictor,Cdk8,Actn1,Stk25,Rassf1,Tacc1,St5,Tead2,Dpysl2,Tagln,Synm,Igf2bp1,Slc9a9,Eif4e3,Myd88,Stk39,Htr1b,Dync1i2,Smpdl3a,Nabp2,Plekha2,Zfp91,Fam118a,Tbc1d2b,Rnf14,Stim1,Snai1,Mapk9,Fat1,2010111I01Rik,Gpcpd1,Il6st,Camk2n1,Cox5b,Prdm1,Inpp5a,Pkig,Pkia,Isca1,Trappc6b,Nectin2,Pdlim7,Rab31,Fabp7,Lrrc8a,Dirc2,Ncald,Itga7,Impad1,Thra,Maf1,Epb41l2,Tmem50b,Ndrg3,Zfp46,Dynlt3,Psmd8,Mtap,Bmp5,Ywhab,Scarf2,Cmtm3,Mrvi1,mtAtp6,Ncor2,Uqcrc2,Adprh,Camk1,Klhdc8b,Rnf122,Senp6,Aebp2,Ddx3y,Zfp362,Cacnb3,Rnase4,Ptpra,Raph1,Tmem30a,Zfp950,Mafk,Atraid,Arhgap31,Ncor1,Ext2,Tmem37,Adgra2,Cers5,Lancl1,Mapre2,Pip5k1c,Timm17a,Cbfa2t3,Rgs3,Cttn,Stard3,Csnk1d,Anpep,Dctn4,Calm3,Yaf2,Siah1a,Adamts8,Manbal,Plin3,Fblim1,H13,Glipr2,Cdc42ep1,Vdac1,mtAtp8,Ecscr,Carmil1,Fam162a,Sult1a1,Mbd2,Desi2,Nox4,Bicd2,Wasl,Rbfa,Esyt1,Bcam,Gnb1,Sgip1,Meis3,Purb,Gem,Angpt1,Elmo2,Uqcr10,Abi2,Ndufs5,Slc9a3r2,Iqgap1,Extl3,Syne1,Sppl3,Atp2b4,Ate1,Ifnar2,Kdm5a,Dennd5a,Gprc5c,Cxxc5,

Cluster31 Pericyte/smooth muscle

Top2a,Hist1h1b,Acta2,H2afz,Rgs5,Pclaf,Cenpf,Ube2c,Tubb4b,Itga1,Hmgb2,Higd1b,Cdca8,Cks2,Birc5,Smc2,Mki67,Cdk1,Prc1,Stmn1,Ndufa4l2,Tagln2,Myl9,Serpine2,Crip1,Tpm2,Fabp5,H2afx,Smc4,H1fx,Tuba1b,Spc24,Cenpa,Hist1h2ap,Cdca3,Cenpe,Tubb5,Nrep,Cks1b,Tyms,Col4a1,Cdc20,Pbk,Racgap1,Tpx2,H2afv,Ebf1,Tubb6,Cspg4,Tmpo,Ccnb2,Cald1,Ccnb1,Spc25,Tuba1a,Mcam,Tk1,Lmnb1,Ran,Zic1,Gjc1,Gm13889,Hist1h1e,Nusap1,Kif22,Cdkn2d,Ube2s,Ccna2,Gng11,Pimreg,Ccdc34,Apold1,Notch3,Kif23,Mxd3,Lgals1,Mfge8,Hmgn2,Jpt1,Pde5a,Rrm2,Selenoh,Plce1,Hspb2,Gucy1a1,Ckb,Pdlim1,Pcdh18,Hist1h2ae,Tpm4,Knl1,Ranbp1,Meg3,Trpc6,Tacc3,Gucy1b1,F2r,Hmmr,Tm4sf1,Rrm1,Kif11,Gja4,Aurkb,Arl6ip1,Neil3,Fbxo5,H1f0,Cox4i2,Myo1b,Kif20b,Calm2,Ckap2l,Slc12a2,Zeb2,Psip1,Uaca,Ddah2,Dnajc9,Ndc80,Cryab,Nucks1,Atad2,Mis18bp1,Tagln,Ckap2,Kif15,Col4a2,Tipin,Prrx1,Dut,Knstrn,Pmp22,Dmd,Cdkn3,Sgo2a,Melk,Fads3,Hmgb1,Cdkn2c,Marcks,Cenpm,Prdx4,Cenpp,Fermt2,Rangap1,Rbm3,Ptk2,Shcbp1,Lurap1l,Tbx2,Depdc1a,Lmna,Slbp,Usp1,Rarres2,Lig1,Fam162b,Rasgrp2,Atp1a2,Nde1,Banf1,Snrpd1,11-Sep,Ift43,Pdgfa,Ezh2,Ncapd3,Bub1,Nuf2,Clspn,Vstm4,Idh2,Plk1,Aurka,Atp5b,Des,Arpc5l,Mprip,Parm1,Esco2,Ppia,Dlk1,Cdca2,Hmgn1,Mrpl18,Nbl1,Anxa5,Mgp,Tubg1,Anp32e,Dbf4,Ascl1,Igfbp7,Incenp,Plat,Rad51ap1,Arhgap42,Dtymk,Hmgb3,Foxm1,Tbxa2r,Anp32b,Anln,Vcl,Pdlim2,Gmnn,S1pr3,Mad2l1,Lama4,Rnaseh2c,Hirip3,Ets1,Cenpw,Lsm2,Bub3,Nr2f2,Kcnj8,S100a11,Nxt1,Kif4,Srsf7,Ifi27,Sae1,Manf,Phlda1,Nt5dc2,Cd248,Dek,Tspan17,Cit,Timp3,Smc1a,Prkar2b,Acsl3,Abcc9,Sumo2,Ckap5,Cnn2,Uba2,Nkd1,Cfh,Mrpl51,Hjurp,Bub1b,Ddx39b,Arhgef17,Farp1,Sema6d,Atp5o.1,Pdgfrb,Trip13,Ncapg,Ndufab1,Smc6,Cisd3,Slc29a1,Cenpq,Ncapd2,Csrp1,Gm

14964,Loxl2,Ncam1,Nrarp,Rad21,Rasl11a,Gnb4,Aspm,Hes6,Crip2,Epas1,Pgp,Nsmce1,Pkig,Lhfp,Hnrnpa2b1,Spon2,Tuba1c,Dlgap5,Car2,Myl12a,Khdrbs3,Rnf26,Myl6,Vtn,Fam111a,Anxa1,Ska1,Topbp1,Atp13a5,Diaph3,Atp5d,Flna,Exosc8,Fam118a,H3f3b,Tnfaip1,Foxs1,Srsf3,Mrpl12,Hes5,Nudt21,Cpe,Rcc1,Rhoc,Ifitm3,Calm3,Espn,Atp1b2,Rap2a,Pmf1,Tspan15,Acadl,Vasp,Clic1,Bok,Ednra,Nsd2,Prim1,Nme1,Cox8a,Heyl,Kif2c,Sapcd2,Plekhg2,Alad,1810037I17Rik,Nsmce4a,Daam2,Pde8b,Snrpb,Ect2,C1qtnf6,Rfc5,Ywhah,Inpp4b,Nrm,Endod1,Rgs4,Atp5g3,Zwilch,Ube2t,Ptma,Axl,Gper1,Ccnd1,Dynlt1f,Pik3cd,Stard8,Ssrp1,Rnaseh2b,Pde3a,Ywhaq,Vdac3,Gm47283,Asf1b,Ssna1,Efhd2,Tgfb1i1,Dhfr,Myh9,Ppp1r14a,Cavin3,Ndst3,Prdx5,Ddx39,Tfdp1,Cysltr2,Baz1b,Maged2,Hnrnpab,Pcna,Krcc1,Iqgap1,Specc1,Ralgapa1,Cmc2,mt-Cytb,Mcm7,Rfc4,Lsm5,Ndufb8,Lsm8,Kpnb1,Nasp,Map3k20,Etfb,Hsp90aa1,Ndufv1,Txn1,Pls3,Iqgap3,Il34,Rfc2,Dynll1,Enpep,Hist1h3c,Fam81a,Lrrc8b,Ndufs7,Tspan12,Ptp4a3,Rrad,Pten,Ptms,Plekhh3,Ccdc88a,Ajuba,Dhx58os,Emp2,Srsf9,Lmf2,Cycs,Gapdh,Cenph,Uhrf1,Rock2,Nudc,Spag5,Ech1,Sdha,Rab13,Ndufb10,Pea15a,Lamb1,Hnrnpu,Fry,Larp7,Kif20a,Med30,mt-Nd1,Ndufv2,Mdh2,Art3,Itgb1,Tbc1d1,Hat1,Kpna2,Ncapg2,Pgam1,Coq7,Gtf2a2,Ppp1ca,Elof1,Fkbp2,Hpf1,Aard,Stc1,Emsy,Gipc1,Ncaph,Rbl1,Hmgcs2,Mns1,Cenpn,Cyc1,Mdh1,Immt,Cd81,Uchl1,Plxnb2,Pitpnc1,Ppp1r12a,Rhno1,Sgo1,Slc19a1,Mcm6,Mastl,Plk4,4930523C07Rik,Filip1l,Cobll1,Lamb2,Rasl12,Rheb,Cbx3,Csnk1e,Cntln,Frat2,Dpy30,Anapc11,Fabp7,Timm50,Rdm1,Rorb,Agrn,Prkar1a,Apbb2,Psmb6,Cenpk,Smchd1,Commd1,Nudt4,Lsm4,Clip1,Utrn,Psmc3ip,Txn2,Tsen34,Gnb2,Notch1,Kif18a,Haus4,Bmp1,Rgs16,Lamc1,Pde4d,Psmc3,Lsm6,Dazap1,Trappc1,Cep192,Etfa,Tnks1bp1,Rgs7bp,Oxct1,Ndufa12,Pfn1,Tns1,Slc44a2,Mlf1,Mrps17,Fam162a,Slc25a5,Cdc45,Bcl2l12,Ing1,Rapgef5,Rbbp7,Psmd13,Capg,Ccdc12,Cdc42bpa,Ccdc80,Cox5a,Dnmt1,Eif1ad,Egflam,Lockd,Rassf1,Psmc2,Ilk,Ggt5,Afap1l2,Hmgn3,Uqcrc1,Cdc7,Atp5h,Xist,Timm13,Mnd1,Chaf1b,Cavin1,Lims1,Lrrc32,Cox4i1,Snrpa1,Mast2,Gng2,Mbd3,Cenpl,Ap1s1,Psmd8,Aspn,Gatad1,Mical1,Trim59,Ccdc82,Gins2,Cbx5,Miip,Tmem45a,Lsm3,Eny2,Pin1,Emp3,Angptl4,Prkcb,Stub1,Rock1,Vsx2,Wls,Psmc5,Sdhd,Trir,Nelfe,Pold2,Cby1,Rem1,Brd8,Fstl1,Ndufaf2,Pmepa1,Epb41l1,Gm14005,Srsf1,Ctcf,Cdk6,Egln1,Raph1,Cacna1c,Cdk5rap2,Nes,Hdgfl2,B230118H07Rik,Vim,Oaz2,Cpa1,Rpa3,Acat1,Fam96b,Cox5b,mt-Nd4,Rnaseh2a,Nono,Ldha,Cbx1,Cep57,Oard1,Cep89,Rax,Rbmx,Tln1,Rpn2,Trim28,Cdh6,Pkmyt1,Acp1,Itga4,Nup62,Hmg20b,Ywhae,Nelfcd,Alyref,Arhgdib,E2f8,Xpo1,Smarcb1,Pcmt1,Rnf187,Syt11,Pla1a,Csrp2,Dbi,U2af1,Wdhd1,Atp5f1,Ubald2,Tmem30a,Pdzd2,Brca1,Vdac1,Cnih4,mt-Nd2,C330027C09Rik,Mef2a,Rad51,Ptbp1,Tcf19,4930503L19Rik,Brca2,Immp1l,Ndufb9,Cep55,Bora,Cspg5,Mapk12,Hnrnpr,Mcm2,Bmp5,Fen1,Ak3,Slc43a3,Impdh2,Raly,Ywhab,Tnfrsf21,Mustn1,Psmc6,Ak6,Smc3,Htr1b,Actn4,Bcas3,Sdc2,Cxxc5,Actr3,Bcam,Brip1os,Hnrnpf,Lbr,Med9,Psmb5,Hells,Syne2,mt-Atp6,Cops6,Cers4,Smarcd3,Cd9,Phc2,Diablo,Nin,Ctc1,Mrpl28,Dgkh,H3f3a,Slc38a11,Col2a1,Uqcrb,Camk2n2,Hist1h4i,Hnrnpd,Sac3d1,Uqcrq,Zfp91,Slc25a3,Ogdh,Arid5a,Dzip1l,Plcl1,Cdk4,Zic4,Hdgf,Atp5c1,Actn1,Hspa14,Sf3b6,Stk39,Gamt,Cfl2,Atp5a1,Dhrs4,Csk,Abhd8,Arhgap1,Emc8,Nnmt,Tmem109,Usp25,Pbdc1,Cox7a2,Gstk1,St3gal5,Cenpt,Spdl1,Agpat5,Psmb2,Itgb3bp,Lmnb2,Fam20a,Maz,Myadm,Dag1,Pfdn1,Rpa2,Anapc15,Cenpu,Galk1,Grpel1,Rgs6,Tmem165,Hist1h1a,Prdx3,Tmx2,Emg1,Rtn3,Gins4,Eva1b,Tdp1,Adprh,Arhgef7,Vbp1,Cdca5,Psmd7,Ehd2,Phip,Hnrnpa3,Nab1,Aoc3,Rfc3,Kif21a,Tpi1,Fam107b,Bola1,Olfml2b,Uqcr10,Eloc,Cnot6,Srsf2,Ttk,Kmt5a,Serpinh1,Nek2,Tomm22,Ifitm1,Cfap20,Armcx3,Ppic,Mrpl55,Bcl7c,Tbcb,Prkg1,Phldb2,Eif4a1,Pkd2,Ctdspl,Hbegf,Ybx1,Pon2,Hist1h2ab,Cdc25c,Ndufs3,Ndufb7,Rfwd3,Ndufa11,Tm

em14c,Hist1h3e,Pcdh1,Lrrcc1,Cirbp,Hdac2,Cdc42ep4,Eif2s3y,Gemin7,Coro1c,Rbbp4,Acyp2,Meaf6,Pck2,Ska2,Pa2g4,Haus1,Snrnp40,Polr2a,E2f1,Fat1,Rac1,Pdcl3,Gpx8,Cenps,Pkia,Bgn,Mark1,Kifc1,Gpcpd1,Eif6,Plscr1,Foxc2,Hp1bp3,Ppp1cc,Mrps14,Pkm,Mtx2,Myef2,Fhl2,Cd151,Rer1,Sugt1,Btbd3,mt-Co2,Hspb1,Mybl2,Phf5a,Clic4,Bccip,Ints11,Itm2a,Mrpl13,Mrpl14,Yae1d1,Paip2,Pde6d,Dnajc2,Mrpl58,Lamtor2,Ilf2,Cdc25b,Pms2,4930579G24Rik,Mtap,G3bp1,Hdac1,Dda1,Pgf,mt-Co3,Tox3,Pth1r,Alg14,Gm42047,Mfsd10,Hmox2,Plod2,Abi2,Samm50,Fh1,Smc5,Mrpl15,Pias3,Tns3,Rad18,Kmt2a,Pdlim7,Samd1,Mical2,Reep4,Gpx4,Rap1a,Tmem138,Minos1,Dnph1,Bola3,Itgb5,Ptges3,Arhgef40,Zgrf1,Tex30,Metrn,Ndufs2,Atad5,Troap,Unc13c,Reep5,Atp7a,Ndufc1,Snrpc,2310009B15Rik,Erh,Setd1b,Med19,Fam193b,Ncaph2,Nid1,Esyt2,Ndufc2,Ube2a,Hist1h1d,Pgk1,Hspa1a,Ctbp1,Mndal,Gspt1,Galnt16,Selenof,Cuta,Cyp39a1,1110065P20Rik,Matr3,Gm32051,Iigp1,Yaf2,Sfxn1,Fignl1,9930111J21Rik2,Brd3,Ybx3,Arhgef6,Ifi203,Aebp2,Siva1,Ctxn1,Cdc123,Myg1,Gas2l3,Lhx2,Kcnk1,Gna11,Timm17b,Amotl1,Wee1,Dctpp1,Cenpx,G2e3,Top2b,Capn2,Wdr76,Ndufs5,Ppil1,Map2,Uqcrc2,B3glct,Ahctf1,Syce2,Vdac2,Mrpl57,Hdhd2,Cbfb,Pola1,Ssb,Hspd1,Cops3,Mcm5,Espl1,Arhgap10,Mrpl20,Chn1,Med28,Nr2f6,Bcas2,Taf1,Adora2a,Slc25a25,Smarcc1,Ndfip2,Smarca4,Baz1a,Prkag1,Ndufa13,Syncrip,Cep83,Gcnt2,Rnf152,Derl2,Odf2,Copz2,Skp2,Dcakd,Orc6,Mis18a,Pcbd2,Ap2m1,Nup37,Fxn,1110008F13Rik,Chp2,Rnf126,Psma2,Ube2m,Mettl9,Puf60,Rex1bd,Adcy6,Adap2,Tead1,Klhl23,Uqcr11,Trappc5,Mrpl17,Mrpl49,Lancl1,Nop56,

Smooth muscle cells

Cluster24
Acta2,Tagln,Myh11,Crip1,Myl9,Rgs5,Meg3,Notch3,Dlk1,Rgs16,Gucy1a1,Mgp,Agt,Gjc1,Tpm1,Ndufa4l2,Ptp4a3,Gadd45g,Tpm2,Filip1l,Itga1,Limd1,Gm13889,Mef2c,Uba2,Rasl11a,Mfge8,Abcc9,Mustn1,Tinagl1,Socs3,Mylk,Cald1,Phlda1,Pdgfrb,Cox4i2,Gadd45b,Ebf1,Gpc6,Col4a1,Aoc3,Timp3,Lhfp,Mcam,Aspn,Mgst3,Mprip,Ednra,Cdkn1c,Kcnj8,Ppp1r15a,Myo1b,Des,Gsn,Serpini1,Pten,Pdgfa,Nr2f1,Nrep,Rcan2,Tbx2,Snrk,Slc12a2,Cygb,Ntm,Epas1,Fbn1,Txnip,Errfi1,Col4a2,Cavin1,Tm4sf1,Lgi1,Pde3a,Plce1,Itga7,Prrx1,Prkg1,Zeb2,S100a11,Tuba1c,Serpine2,Rgs7bp,Ano1,Sbno2,Gja4,Adap2,Vcl,Itgb1,Rasl12,Parm1,Gucy1b1,Ndrg2,Ppp1r12a,Malat1,Flna,Mark1,Axl,Lamc1,P2ry14,Rasgrp2,Klf9,Ccdc80,Lpp,Ccdc3,Arhgef17,Maged2,Id3,Bcam,Cd248,Pdlim1,Arid5b,F2r,Sparcl1,Peak1,Fbn2,Cspg4,Atp1b2,Rbpms,Gng11,Carmn,Foxs1,Nab1,Pgf,Nr2f2,Sdc2,Zfhx3,Gas1,S1pr3,Mxd4,Daam2,Tbc1d1,Ggt5,Adamts2,Syne2,Tesc,Cxcl1,Btg2,Cebpd,Ppp1r12b,Myh9,Igf2r,Map3k20,Cpe,Tbx3os1,Lgalsl,Galnt16,Nrip2,Oaz2,Higd1b,Angpt2,Anxa1,Emp2,Emid1,C1s1,Epn2,Utrn,Arhgap42,Rit1,Rbpms2,Fam162a,Slc38a11,Map4k4,Trim2,Map1lc3a,Olfml2b,Ebf2,Amotl1,Dlc1,Lamb2,Ajuba,Dtx3,Tgfb2,Lbh,Farp1,Ralgapa1,Tns1,Cd9,Arhgap10,Cavin3,Ifitm3,Ptprf,Ier5l,Adamts4,Gnb4,Iqgap1,Inpp4b,Ehd3,Ifitm1,Steap4,Sema5a,Fads3,Rarres2,Cav1,Gnas,Fxyd1,Rap2a,Timp2,Wtip,Pcdh18,Cnn2,Lin7a,Hspa1a,Csnk1e,Fermt2,Mmp14,Ift43,Dgkg,Lmna,Arid5a,Sox5,Ptms,Cacna2d1,Rcsd1,Myl6,Pmp22,Rasd1,Tpm4,Hspb1,Fstl1,Akap12,Plekhg2,Pear1,Pard6g,Arhgef7,Hspb2,Fam107b,Actn1,Bcr,Junb,Cyth3,Tln1,Rnf152,Gm12216,Foxc1,Mrvi1,Bgn,Zfp46,Snai1,Agap1,Lgals1,Peli1,Cdk6,4931406P16Rik,Cacnb2,P3h3,Tspan3,Ppp1r12c,Eif4a2,Jun,C1qtnf6,Cdc42bpa,Rhoj,Pde5a,Sdc1,Gem,4921524J17Rik,Prkacb,Pcdh1,Rock1,Plekha2,Bdnf,Heyl,Epb41l1,Rock2,Adamts1,Jam3,Syne1,Lama4,Ramp1,Vstm4,Tspan17,Xist,Olfml2a,Arhgef25,Fam110b,Tgfb1i1,Rgs4,Pcp4l1,Spry4,Fzd4,Gab1,Thy1,Pde4d,Gli

pr2,Rrad,Itgb5,Trpc1,Picalm,Osmr,Pik3r1,Dnajb4,Serping1,Marveld1,Laptm4a,Agrn,Slc9a3r1,Mmp11,Atp2a2,Mapre2,Serpinh1,Pcolce2,Uaca,Zfr,Tmod3,Slc25a25,Lmod1,Foxc2,Calm2,Golim4,Bmpr1a,Ifngr2,Tns3,Apbb2,Endod1,Pde1a,Nptn,Dstn,Fhl2,Lims1,2010111I01Rik,Nrarp,Cdh6,Airn,Igfbp7,Antxr1,Ndfip2,Ctdspl,Egflam,Klhdc8b,Atp1a2,Mef2d,Ppp1r14a,Anxa6,Cdc42ep4,Slco3a1,Mical1,Ldlrap1,Mbnl2,Adamts9,Tra2a,Kifc3,Ntrk2,Dmd,Dzip1l,Atf3,Plekhh3,Ate1,Snx18,Brd4,Tnfrsf21,Klf7,Cirbp,Sorbs1,Prkar2b,Fam241a,Afap1l2,Nudt4,Pls3,Fam104a,Olfr558,Rhoc,Dgkh,1810032O08Rik,Mef2a,Enpep,Ncald,Itga4,Ripk1,Hey2,Map7d2,Il13ra1,Sv2a,Rras,Itm2c,Rbfox2,Col5a1,Ptpn9,Ahcyl1,Arrdc3,Ywhaz,Mtdh,Ehd2,Slc44a2,Crtc3,Dock10,Adgra2,Actn4,Tob2,Selenom,Tspan12,Lpl,Hrc,Rtn4rl1,Plec,Hmgn3,Clk1,Arap2,Ddit4,Naalad2,Wnt5b,Arhgap1,Son,Grk3,Arhgap6,Rab11b,Eid1,Csnk1g1,Peli2,Nbl1,Ptpn1,Fry,Cisd3,Snrnp48,Pkig,2310022B05Rik,Cacna1c,Pcdh19,Ubb,Myof,Tmem165,Ilk,Hdgfl3,Ptk2,Cul1,Aph1b,Ctxn1,Tspan15,Fkbp10,Jag1,Tacc1,Ssbp2,Tsc22d4,Itpripl2,Itgav,Dnajc10,Rap1a,S100a6,Ktn1,Csnk1a1,Pck2,Tbx3,Prkar1a,Cited2,Runx1,Klhl23,Arpc5,Btd,Slc25a4,Trpc6,Sema6d,Sparc,Adcy6,Itm2b,Snhg18,Tnrc6c,Cyb5r3,Ets1,Gcnt2,Esam,Fkbp9,Ddr2,Neo1,Ywhab,Serinc3,Stc1,Manbal,Notch1,Thra,Clstn1,Btg1,Rsbn1l,Luzp1,Samd4,Stk24,Dag1,C1qtnf2,Pdlim7,Eif4ebp2,Dync1i2,Nes,Fus,Sort1,Dgkb,Atp2b4,Pth1r,Tead2,Raph1,Agpat5,H3f3b,Zcchc24,Syde1,4833422C13Rik,Hspa1b,Tdp1,Ebf3,Cyp7b1,Rnf14,Plxnb2,Thbs1,Hlx,Ubr5,9530068E07Rik,Bcas3,Crip2,Impad1,Adap2os,Ndufs7,4930523C07Rik,Pofut2,Eif5b,Tsc22d3,Chd4,Ap2a1,Purb,Tmem50b,Cd81,Eif4g3,Scara3,Bace1,Zcchc14,Hspa5,Adprh,Bach1,Plcb4,Gamt,Sox4,Coq10b,Lrrfip2,Fyttd1,Mdh1,Rerg,Tcf7l1,Spats2l,Stac,Stk38l,Fndc3b,Capns1,Ttc28,Aplp2,Runx1t1,Adgrl1,Nexn,Tnks1bp1,Vangl2,Pttg1ip,Nfe2l1,Msrb3,Pgrmc1,Ppic,Nktr,Ddit4l,Gm14005,Lama2,Dctn1,Nedd4,Kdm5a,Ptprk,Sesn3,Pbxip1,Apold1,Lzts2,Per1,Vim,Mcrs1,Pxdn,Filip1,Cd93,Specc1,Nbeal1,Pawr,Herc4,Pdcd5,Scarf2,Abi2,Art3,Tmem246,Esyt2,Camk2d,Rassf1,Egr1,Nr4a1,Btbd3,Msx1,Trib2,Klhl24,Ankrd12,Pdcd10,Ccnd2,Smim3,Slc41a3,Spag9,Ccdc141,Ext2,AC160336.1,Uqcrc2,R3hdm2,Anapc5,Cobll1,Fam20a,Nelfb,Dact1,Nufip2,Xpc,Grk5,Fbxo32,Ssr3,Dab2ip,Lmo7,Cox8a,Ndst1,Tanc1,Strn3,Npy1r,Ier5,Slc16a2,Cd151,Bcl9l,Igfbp4,Pard3,Anxa5,Aebp2,Rsrp1,Vasn,Srpk2,mt-Nd4l,Arhgef6,Frmd4a,Dvl3,Ndufb9,Tra2b,Ccdc50,Krit1,Nckap1,Ppp2r2a,B230219D22Rik,Pitpnc1,Clcn4,Gucy1a2,Etnk1,Ankrd44,Nhsl2,Prkar1b,

Macrophage

Cluster5:

Pf4,Tyrobp,C1qb,Ifi27l2a,Fcer1g,C1qa,Aif1,C1qc,Rgs10,Fcgr3,Apoe,Lyz2,Ccl12,Tmsb4x,Ptpn18,Ltc4s,Gmfg,Ucp2,Ctss,Spi1,Ftl1,Fth1,Trem2,Rac2,Sh3bgrl3,Coro1a,Blvrb,Ms4a7,Cyba,Lgmn,Snx2,Ms4a6c,Arpc1b,Clta,Arhgdib,Cotl1,F13a1,Fcrls,Trf,Bcl2a1b,Ehd1,Psmb8,Plin2,Cd68,Ninj1,Limd2,Unc93b1,Snx6,Arpc3,Ostf1,Prdx5,B2m,Fcgr2b,Ctsd,Vamp8,Fcgrt,Dab2,Slfn2,Selenop,Snx5,Gabarap,Cst3,Ctsb,Bst2,Npc2,Pfn1,Csf1r,Use1,Ap2s1,Hexa,Cstb,Ctsz,Laptm5,Capza2,Mt1,Clic1,Serp1,Ehd4,Pnp,Gdi2,Arpc2,Maf,H2-K1,Atp6v1g1,Lgals1,Rps29,Actb,Bri3,H2-D1,Cfl1,Lamp1,Ckb,Fau,Akr1a1,Sh3glb1,Gng5,Rps11,Ctsc,Cdc42,Epn1,Etfb,Gpx4,Psme2,Hprt,Tmem176a,Hint1,Cenpx,Vapa,Mrpl23,Necap2,Timm8b,Hsbp1,Taf10,Ndufb7,Ubl5,Tma7,Ost4,Rpl10,Hras,Plgrkt,Ranbp1,Cuta,Rbm3,Ndufs8,Pgls,Tpt1,Pitpna,Vps35,Ctsh,Idh2,Stx7,Hcfc1r1,Ndufb8,Cox8a,Txnip,Tbcb,Chmp4b,Tmem37,Arpp19,Pkig,Anapc13,1110008F13Rik,Sirt2,Atp6v0e,Bag1,Timm13,Scp2,Ypel3,Pold4,Cops9,Rpl27a,Litaf,Pomp,Psenen,Trappc2l,Rps25,D8Ertd738e,Smdt1,Tspo,Ubxn1,Ten1,Ube2l3,Edf1,Cuedc2,Gapdh,Tubb2a,Scamp2,Rab7,Gm42418,Cmpk1,Cltc,Chmp2a,Aes,Jpt1,Nhp2,Eef1d,Itm2b,Dazap2,Mrpl28,Card19,Vdac2,Ppp1ca,Mrpl57

,Rnaseh2c,Man2b1,Oaz1,Uqcrb,Lamtor2,Stmn1,Hmgn2,Cyb5a,Ppia,Aldh2,Fkbp2,Cox14,Iscu,Gipc1,Cox7b,Rac1,Uqcrq,Nedd8,Rpl36al,Mrps14,Trappc3,Sec61b,Sdhd,Rragc,Sod2,Psma7,Fam96a,Lamtor5,Ufc1,Scand1,Atp5j2,Cyfip1,Tmem86a,Grb2,Rps12,Cebpb,Llph,Elof1,Gusb,Actr2,Rps9,Atp5g1,Mrps24,Psap,Cwc15,Rpl35,H2afj,Ssu72,Dnajc19,Ndufab1,Ndufc1,Srsf9,Akirin1,Psmc5,Rap1a,Snx17,Kras,Atp6v0d1,Ndufv3,Sf3b5,Ufm1,Ndufa2,Rpl22,Srp14,Ndufa7,Rpl26,Ndufb10,1810058I24Rik,Capns1,Twf1,Atp5k,Atp5o.1,Basp1,Snf8,Mrps16,Ralb,Rps3,Idh3g,Ndufs6,Kxd1,Ybx1,Ndufa9,Rplp1,Vamp3,Agpat3,Srp9,Mrps33,Gm16286,Chmp1a,Mpc1,Rnh1,Tbca,Rpl19,Mrpl42,Rps24,Gna12,Hnrnpf,Rplp2,Rpl35a,H2afy,Mkrn1,Psma6,Rtf2,Mrpl54,

Cluster14
Apoe,Mrc1,Lyz2,Ccl2,C1qa,Neat1,Fcgr2b,Ccl3,Csf1r,C1qc,Ccl7,Cx3cr1,Selenop,Stab1,Lgmn,Lyve1,Ier3,Fcgr3,C1qb,F13a1,Maf,Dab2,Fcer1g,Ctsc,Kctd12,Trem2,Ctsb,Zfp36,Grn,Tyrobp,Pf4,Ms4a7,Laptm5,Dusp1,H2K1,Trf,Hmox1,Ccr1,Ccl24,Adrb2,Sirpa,Atf3,Jun,Cxcl2,Ccl12,Nfkbia,Ctss,Clec4n,Unc93b1,C3ar1,Fcgrt,Cited2,Fcrls,Klf2,Abca1,Egr1,Plin2,Mafb,Marcksl1,Cd86,Fosb,Klf6,H2D1,Spp1,Fcna,Adgre1,Hpgds,Ctsa,Ctsd,Rnase4,Nfkbiz,Ednrb,Ltc4s,Ly86,Man2b1,Cd68,Serinc3,Gatm,Cd83,Fcgr1,Tnf,Ccl4,Cyba,Pld4,Ier5,Hexa,Hpgd,Ms4a6c,Ctsl,Ms4a6b,Cp,Cybb,Pla2g7,Cst3,Ehd4,Rgs10,B2m,Nfkbid,Gbp7,Rhob,Plek,Jund,Bst2,Ctsz,Ddit3,Blvrb,C5ar1,Abhd12,Cd53,Ier2,Fos,Ifi27l2a,Csf2rb,Sdc4,Btg2,Folr2,P2ry6,Ptgs1,Rsrp1,Evi2a,Ptpn18,Slc40a1,Ifi207,Hgsnat,Ubc,Snx5,Snx2,Txnip,Asah1,Snx6,Fam46a,Tbxas1,Atp6v0b,Ftl1,Lamp1,Dnajb1,Creg1,Gas6,Pou2f2,Aif1,Psap,Cd300a,Egr2,Scamp2,Tmem176a,Rcbtb2,Slfn2,Gmfg,Atp2b1,P2rx7,Cyth4,Gm26532,P2rx4,Ly6e,Cd38,Gm26522,Cltc,Cd36,Ssh2,Tgfb1,Cd84,Ncf2,Dnase2a,Clta,Tmem176b,Bcl2a1b,Gpr183,Ehd1,Irf8,Glul,Junb,Runx1,Klf4,Ifngr1,Spi1,Mef2c,Hexb,Neurl3,Ccnl1,Srsf2,Ap1b1,Tnfaip8l2,Brd2,Cebpa,Rnf130,Ms4a6d,Lipa,Fyb,Slc9a9,Npc2,Hacd4,Smap2,Ppp1r10,Pla2g15,Mfsd1,Lcp1,Tpp1,Pnp,Mat2a,Gns,Nrros,Cd28,Slco2b1,Bmp2,Lrrc25,Cd163,Clec4a2,Ptprc,Cfh,AC149090.1,Lyl1,Mylip,Rab3il1,Dnajb9,Il6ra,Ptpn6,Ctsh,Gusb,Mpeg1,Arhgap17,Dhrs3,Il4ra,Gimap6,Serp1,Rgs2,Ptafr,Nrp1,Mcl1,Fam105a,Zfand5,Gls,Dusp2,Rbpj,Clec4a1,Gpr34,Ucp2,Rac2,Il10rb,Zfp36l1,Tbc1d4,Tlr2,Cd300c2,P2ry12,Wwp1,Eif5,BC005537,Ncf1,Cyfip1,Lpar6,Eps8,Cd33,Cd164,Sgpl1,Stk17b,Zeb2,Lamp2,Zcchc6,Tmem86a,Man1a,Chka,Casp4,Vsir,Nckap1l,Icam1,Rasgef1b,Frmd4b,Egr3,Tmsb4x,Rnasel,Gas7,Stard8,Arrb2,Cfp,F630028O10Rik,Msr1,Tnfaip8,Lrp6,Atp6v0a1,Ang,Dusp6,Nfam1,Zeb2os,Ddx3x,Hfe,Mbnl1,Coro1a,Rin2,Ifi204,Cebpb,Kansl1l,Cd14,Tfe3,Slc11a1,Atp13a2,Pxdc1,Ifrd1,Fermt3,Ninj1,Efhd2,Ophn1,Slc15a3,Sertad1,Lgals3bp,Skap2,Sdcbp,Limd2,Pnrc1,Tgfbr2,Abca9,Hspa5,Cd200r1,Mtss1,Rilpl2,Ddx5,Arpc1b,Itm2b,Pnpla7,Smagp,Mertk,Hexim1,Pik3cg,Dse,Daglb,Ddx60,Ifi202b,Cd180,Colgalt1,Gm6377,Arhgap45,Lyn,Psmb8,Erp29,Gpx3,Nabp1,Tm6sf1,P2ry13,Fth1,Cstb,Tsc22d2,Adcy7,Rap1b,Arhgap30,Fcho2,Cndp2,Inpp5d,Bin1,Man1c1,Tmem37,Baiap2,Vamp8,Plekho2,Cd47,Cd74,Cd37,Lst1,Litaf,Clcn5,Slc43a2,Mpp1,Khk,Mfsd11,Dock2,H2-Q4,Pld3,Cotl1,Samhd1,Wsb1,Clk1,Arhgap19,Bmp2k,Lpcat2,Vps18,Adam33,Ostf1,Blvra,Sun2,Tlr7,Rapsn,Rgl1,Fgd2,Rab8b,Spint1,Slc16a6,Arl4c,Hspa1a,Atp6v1b2,Rnf150,Use1,Atp6v0e,Il21r,Gpr160,C2,Ppp1r15a,Tmem106a,Slc37a2,Atp6v1a,St8sia4,Igsf6,Neu1,Flcn,Slc17a5,Tob2,Gnpda1,Slc29a3,Myo5a,Polr2a,Arpc5,Milr1,Retreg1,Atp6ap1,Atf4,Rab11fip5,Capza2,Cnppd1,Pmp22,Washc2,Srsf7,Syngr1,Sqstm1,Ccdc152,Tmem55b,Tagap,Tln2,Jmjd1c,H2-T23,Abcc3,Morc3,Rel,Gem,Slc7a8,Zfp36l2,Rcan1,Snx8,Ptprj,Cln8,Rragc,Serpinb8,Itga6,Gm2a,Oser1,Scpep1,Slc12a9,Srgn,Tgfbi,Hnrnpa2b1,Glmp,Map3k8,Aoah,Mob3c,Picalm,Clec4a3,Plxnb2,Rnf19b,Mgat1,Zfp710,Tmbim6,Galc,Hsd17b12,Nhlrc3,mt-

Co1,Soat1,Fam49b,Nufip2,Atp6ap2,Lfng,Marcks,Mknk2,Rab32,Il16,Mafg,Syk,Naga,Dclre1c,Npl,Tra2a,Tor1aip1,Plekho1,Synj1,Trim30a,Hhex,Tapbp,Fnip1,9930111J21Rik2,Cmtm7,Sft2d2,Casp8,Gabarap,Cbl,Comt,Itsn1,Lcp2,Ets2,Vav1,Rassf4,Osbpl11,Vwa5a,Bcl10,Sft2d1,Bank1,Was,Engase,Renbp,Orai1,Slc35f6,Fam20c,Kdm6b,Grina,Fkbp15,Grb2,Ptpra,Rhog,Rnf13,Twf1,Pltp,Csf2rb2,Parp14,Bri3,Irf5,Atp1b3,Gmip,Il10ra,Tnfrsf11a,Cd48,Zfp622,Tug1,Gltp,Arid3a,Aup1,Ncoa4,Dock8,Eps15,Dazap2,Ptbp3,Ntpcr,Arpc3,Pip4k2a,Nceh1,Nlrp3,Tifa,Mia2,Ebi3,Fam213b,Cnpy3,Hcls1,Fchsd2,Tmem140,Ap2a2,Cyp27a1,Apoc1,Mid1ip1,Apobec1,Pycard,Anxa3,Ikzf1,Mgat4a,Aldh9a1,Cmtm6,Dnajc13,Raph1,Pepd,Rasa1,Clic1,Ints6,Eif4a1,Cmklr1,Npc1,Hist3h2a,Jpt1,Tk2,Aftph,Dnajb14,Arpc4,1700017B05Rik,Rreb1,Serpinb6a,Ncoa3,Lacc1,Slc38a6,Nfxl1,Mt1,Paox,Trib1,Xbp1,Sec14l1,Ranbp2,Taok3,Camk1,Aim2,Zc3h12a,Rab7b,Tgoln1,Tmem50a,Osm,Tcn2,Reep3,Hprt,Epor,Snap23,BC017643,Traf3ip3,Mndal,Cln3,Sat1,B4galt6,Pacsin2,Cryab,Syngr2,Crlf2,Vps35,Rab1a,AC127341.3,Hck,Cysltr1,Snx30,Fes,Pim1,Arl8a,Gpr137b,Rnf149,Ubash3b,Tnfaip3,Celf2,Ctns,Apbb1ip,Tmem141,Ppp1r9a,Zdhhc14,Ppt1,Cryl1,Dgkz,Slc23a2,Sesn1,Tex264,Dram2,Pde3b,Acp2,Zfhx3,Ifi203,Slc31a2,Dbnl,Irf2,Sh3bp5,Lamtor3,Tifab,Arl8b,Acox3,Prcp,Gcnt1,Fnbp1,Slc46a3,Atp6v1h,Ifnar2,Prkcd,H2M3,Adipor1,Trafd1,Wwp2,Fam174a,Arl4d,Tpst2,Gpsm3,Necap2,Sptlc2,Srsf5,Mknk1,Ahr,Ccdc50,Chd4,Fndc3a,Ccl6,Osbpl9,Sbf2,Abcd2,Cryaa,Tnfrsf1b,Arhgdib,Ppp1r21,Myo1f,Lag3,Tubb2a,Irf2bp2,1Mar,Ctsf,Arhgap25,Nagpa,Calm2,Nrap,Gapvd1,Tiparp,Itgam,Lncpint,Itgb5,Sirt1,Rhoh,Igsf8,Hmox2,Fkbp2,Epsti1,Timp2,Kdm7a,Psmb9,Arl6ip1,Tmem104,Actr2,Hsp90aa1,Gna15,Adam17,Irf7,Srsf6,Nudt16,Akr1b3,Qk,M6pr,Plk3,Hnrnph1,Fam234a,Gla,Mgst1,Pfkfb3,Klf7,Cd63,Mfsd12,Cklf,Grk2,Rbm47,Map3k1,Uap1l1,Adap2,Os9,Slc31a1,Ubap2l,Hivep3,Smim1,Hsd17b11,Derl1,Tpm3,Mepce,Sppl2a,Hk2,Gdi2,Sh3bp1,Stxbp2,Gm4951,B3galnt1,Zbtb7a,Mctp1,Pcna,Tpd52,Tmcc3,Srsf3,Slc16a10,Btk,Snx29,Eef2k,St6gal1,Metrnl,0610012G03Rik,Scamp5,Tspan8,Zmynd8,Tlr13,Shisa5,Lrmp,Tnfrsf13b,Mvp,Rnf213,Iqgap2,Scn1b,Tln1,Abcg3,Eif4a2,Tspan33,Tmem8,Ulk2,Slc20a1,Ccl9,Klhl9,Pcyox1,Scarb2,Mtus1,Epn1,Ppp1r18,Apobec3,Snx24,Tmed5,Ripk1,Tex261,Ppm1h,Cttnbp2nl,Dnaja1,Gja8,Actr3,Gsdmd,N4bp2l1,Nagk,Vps13c,Wdfy3,Fli1,Ly96,Cfl1,Fuca2,Gtf2b,Casp1,Ncstn,Rab7,Rasa4,Fus,Cd79b,Pfn1,Pkib,Stk4,Dpp7,Stard3nl,Impact,Sh3gl1,Vps26a,Naglu,Aagab,Pcf11,Dera,Rlim,Prex1,Rnpep,Hlx,Gt(ROSA)26Sor,Sp100,Pdcd6ip,Tap1,Agtrap,Coro1b,Lgals9,Cdc42se2,Ndel1,Kpna4,Fnip2,Slc25a45,Kdm2b,Elf1,Sord,Zfp90,Psmb10,Aph1c,Herpud1,Sema4a,Cnrip1,Gpr65,Sh3glb1,AI413582,Pik3cd,Dmxl1,Tgfbrap1,Fam173a,Rps6ka1,Dusp7,Stx7,Abcd1,Sqor,Camk1d,Zfp992,Lbh,Elmo1,Klf10,2610507B11Rik,Hps3,Wbp2,Tfec,Ints6l,Pkn1,Cflar,Arhgap18,Adam9,Selplg,Srgap2,Arhgef3,Herc1,Itgb2,Slc38a7,Sash3,Arhgdia,Mast3,Snx20,Lmbrd1,Pabpc1,Sec11c,Ptpro,Tnfsf12,Ccrl2,H3f3b,Nek6,Htatip2,Nfatc2,Vps29,Larp4b,Myo7a,Relt,Hebp1,Idh2,Sdf2l1,Tmem87b,Tnfrsf1a,Psenen,Amdhd2,B4galnt1,Plagl2,Plekhj1,Sema4d,Wipf1,Prkab1,Rassf2,Themis2,Plbd2,Ms4a14,Sirt2,Arid4a,Mycbp2,Slc12a6,Slc5a3,Rtf2,Birc3,Gpr146,Atp8a1,Exoc3,Rtn3,Tmem243,Hmgcl,Wdr81,Znfx1,Rnase6,Nacc2,AB124611,Pde7a,Galnt1,Ppp1r11,Zbtb4,Eloa,Dcxr,Rad50,Sp140,Mcfd2,Stard9,Slc35c2,Gtf2a1,Zfp703,Susd3,Sh3bgrl3,Ddrgk1,Capzb,Trim47,B4galt1,Wnk1,Acss1,Nr3c1,Mob1a,Aldh2,1700003F12Rik,Hspa8,Cdc42se1,Fgd4,Rab5c,Ftl1ps1,Mdfic,Fbrs,Tanc2,Arf3,Twf2,Slc16a7,Gbp9,Washc4,Rab43,Gsto1,Wdr26,Kansl1,Ccm2,Slc45a4,Relb,Hcst,Eepd1,Prdx5,Vmp1,Rap1gds1,Crybb3,Sh2b3,Atp6v1g1,Canx,Capza1,Heatr5a,Mapkapk2,Myo9a,Acly,Tmem230,Pts,Prkca,Csf2ra,Tbk1,Arap1,Ifngr2,Tbc1d14,Cept1,Arhgap12,Ccdc115,Rab20,Stat6,Sfmbt1,Slbp,Ggta1,Gipc1,Slfn5,Slc2a8,Ehbp1l1,Gm1673,Camk2d,Sash1,Dnm2,Sgpp1,Sp110,Ubn1,Taf6l,Hs6st1,Gm38843,Gaa,Rtp4,Gnl3,Spcs2

Cluster17

Cxcl2,Ccl4,Ccl3,Ccl2,Cd83,C1qa,C1qb,Pf4,C1qc,Tnf,Apoe,Ccl7,Csf1r,Lgmn,Dusp2,Cxcl10,Ctsb,Ctss,Lyve1,Fcer1g,Mrc1,Il1b,Ifi207,Slfn2,Laptm5,Sdc4,Hmox1,H2K1,Lyz2,Klf6,Ccl12,Grn,Tyrobp,Nfkbiz,Zfp36,Atf3,Cx3cr1,C3ar1,Nlrp3,Trf,Plin2,Dab2,Nfkbia,F13a1,Plek,Ier3,Bcl2a1b,Fcgr3,Rgs1,Cebpb,Ctsd,Nfkbid,Rasgef1b,C5ar1,Ms4a7,H2D1,Unc93b1,Gpr84,Neat1,Fcna,Marcksl1,Ctsc,Fcrls,Spi1,B2m,Fcgr2b,Ms4a6c,Cyba,Trem2,Stab1,Tlr2,Ltc4s,Ehd1,Nfe2l2,Ccrl2,Rab7b,Tnfaip3,Cd14,Cd53,Maf,Icam1,Mafb,Mpeg1,Sirpa,Cd68,Ptgs1,Ctsz,Ifrd1,Pim1,Cybb,Mcl1,Psap,Pld4,Dusp1,Srgn,P2ry6,Selenop,Cd86,Irf8,Ifi27l2a,Fcgrt,Irf1,Il1a,Ctsa,Ccr1,Clec4n,Trib1,Serinc3,Fyb,Runx1,Adgre1,Gpx3,Ptafr,Chka,Ncf2,Kdm6b,Man2b1,Gdf15,Rel,Slc15a3,Ucp2,Ly6e,Csf2rb,Igsf6,Tgfb1,Kctd12,Ms4a6b,Ms4a6d,Pla2g7,Lamp1,Bmp2,Aif1,Mt1,Jun,Ftl1,Casp4,Hexa,Bst2,Cd74,Cst3,Ptpn18,Rab20,Ninj1,Ly86,Atp2b1,Rilpl2,Hpgds,Coro1a,Snx2,Ier5,Atp6v0b,Ptprc,Ppbp,Rgs10,Plekho2,Ccl9,Gbp7,Ehd4,Efhd2,Cd300c2,Cd163,Lyn,Ccl24,Scamp2,Npc2,Hgsnat,Serpinb8,Litaf,Dnase2a,Rac2,Ccnl1,Gns,Rnf149,Gmfg,Ctsl,Ubc,Rnase4,Arpc1b,Lrrc25,Nrros,Cxcl1,Fam20c,Nfkb1,Abhd12,Hexb,Sqstm1,Mef2c,Mapkapk2,Evi2a,Snx5,Cotl1,Egr1,Cstb,Asah1,Man1a,Zfand5,Lpcat2,Tpp1,Fcgr1,Lcp1,Gadd45b,Klf2,Il6ra,Junb,Gpr183,Folr2,Eps8,Arl5c,Rhob,Sdcbp,Tmsb4x,Gas7,Mfsd1,Rcan1,Fth1,Ctsh,Tbxas1,Zeb2,Il4ra,Tgfbr1,Pnp,Ifngr1,Clec4a2,Fermt3,Blvrb,Gusb,Top1,Tlr7,Cd33,Cfp,Adrb2,Arl4c,Cndp2,Cyth4,Pou2f2,Inpp5d,Cebpa,Creg1,Cd84,Acp5,Cited2,Sgpl1,Cd52,Neurl3,Dennd4a,Spint1,Fos,Ier2,Lilr4b,Tubb6,P2rx4,Cxcl16,Arhgap17,Alox5ap,Atp6v1a,Plaur,Slc9a9,P2rx7,Snx6,Rap1b,Gatm,Ets2,Vsir,Cyfip1,Cd164,Egr3,Jund,Sh3bgrl3,Lst1,Slc20a1,Cltc,Msr1,Ap1b1,Sod2,Gm6377,Abca1,Rnf19b,Zc3h12a,Ppp1r18,Aoah,Mbnl1,Lcp2,Cd44,Ccl6,Arhgdib,Ifi204,Clta,Tgif1,Picalm,Cfl1,Rab3il1,Hilpda,Rragc,Gna13,Atp6v1b2,Fnip1,Cln8,Lgals3bp,Mtss1,Tlr13,Cd300a,Hcls1,Rnf130,Arpc2,P2ry12,Pfn1,Jmjd1c,Slc43a2,Oser1,Atp6ap2,Cxcr4,Ssh2,Id2,Tnfaip2,Lpin2,1700017B05Rik,Cd38,Fosb,Bmp2k,Capza2,Lacc1,Wwp1,Slc40a1,H2Q4,Zfp622,Slc11a1,Apbb1ip,Tgfbi,Psmb8,Gm2a,Lgals9,Cd180,Serp1,Smap2,Tpd52,Atp13a2,Plekho1,Hfe,Arrb2,Itgam,Irf2bp2,Egr2,Maff,Klf10,Retreg1,Irf5,Il10ra,Rbpj,Skap2,Nckap1l,Ralgds,Ptprj,Osgin1,Slc16a6,Arhgap30,Tfe3,Vps18,Slc7a8,Bcl2l11,Lipa,Rhog,Ostf1,Rgl1,Gnl3,Adam17,Apobec1,Arpc5,Plau,Tiparp,Mafg,Gimap6,Bri3,Tmem37,Arhgap25,Gas6,Eif5,Nfam1,Hcar2,Limd2,Ednrb,Iqgap1,Kansl1l,Syngr2,Ppp1r10,Rgs2,Cmtm7,Actr3,Ncf1,Vmp1,Tmbim6,Hk2,Epn1,Aim2,Zcchc6,Fam49b,Prdx5,Arhgap45,Atp6v1h,Khk,Il17ra,Tnfaip8,Sash1,Shisa5,Ptpn6,Snx8,Sh3bp1,Tmem251,Osm,Mertk,Atp6v0a1,Washc2,Klhl6,Hacd4,Stk17b,Tcirg1,Clic1,Rab8b,Tmem176a,Il10rb,Bach1,Daglb,Lamp2,Filip1l,Rbm47,Sla,H2Q7,Slco2b1,Prkcd,Pxdc1,Apoc1,Slc35f6,BC005537,Wnk1,Fkbp5,Arpc3,Tmem104,Gla,Tnfrsf11a,Samsn1,Tpm3,Gm26532,Arl8a,Soat1,Dock2,Gpr65,Eea1,Ddit3,Wdr26,Hlx,Gabarap,Sde2,Map2k3,Stard8,Baz1a,Tmem176b,Pacsin2,Dgkz,Actb,Frmd4b,Rcbtb2,Zeb2os,Ap2a2,Tln1,Grb2,Tmem189,Mat2a,Lgals3,Mki67,Cdkn1a,Csrnp1,Nhlrc3,Rsrp1,Zfp703,Nab2,Mob3c,Bcl2a1d,Birc3,Cdc42se2,Rin2,Fgd2,M6pr,Ggta1,Il21r,Mgat4a,Gdi2,Cd36,Hivep2,Marcks,Vps26a,Snx30,Colgalt1,Tnfrsf1b,Rab5c,Cmklr1,Baiap2,Btg2,Fam46a,Gcnt1,Tfec,Map3k8,Epsti1,Tmem55b,Vamp8,Peli1,Snx24,Syk,Ddhd1,Rnasel,Eif4a1,H2Q6,Acp2,Hck,Mylip,Bin1,Malt1,Arpc4,B4galnt1,Arl8b,H2afy,Npc1,Flcn,Tuba1c,Mfsd12,Abcc3,Smagp,Stx7,B4galt1,Synj1,Lair1,Aup1,Ophn1,Vav1,Fnbp1,Tmem86a,Clic4,Tcn2,Ppp1r15a,Kras,Nfkbib,Tapbp,Itm2b,Ptbp3,Irf7,Ddx5,Lfng,Clec4a1,Txnip,Hivep3,Rab1a,Tgfbrap1,Myo5a,Tifa,Cd37,Was,Erp29,Plk3,Tbk1,Hhex,Dazap2,Skil,Ntpcr,Neu1,Rab7,Pde4b,Camk2d,Pla2g15,Atp6ap1,Arid3a,Spred1,Scpep1,Anxa3,Pnpla7,Ing2,Tor1aip1,Capza1,Ikzf1,Metrnl,Lamtor3,Larp1,Nrip1,Lilrb4a,Pld3,Capzb,Tgoln1,Galc,Tagap,Pak2,Mpp1,Cflar,Icosl,Glul,Elmo1,K

pna4,Cd47,Camk1,Sh3glb1,Pde3b,Tank,Bcl10,Ap2s1,Gmip,Mef2a,Rrbp1,Mtdh,Wdr91,Emb,Casp8,Ptpn1,Gltp,Nceh1,Pmp22,Csf2ra,Ifngr2,Eif1a,Ncf4,Dhrs3,Rbms1,Rab32,mtCo1,Igf2bp3,Slc23a2,Cass4,Zyx,Plagl2,Trim8,Wsb1,Mapk6,Ahr,Sft2d1,Sun2,Serpinb6a,Fmnl1,Atp6v0e,Trim47,Fkbp2,F630028O10Rik,Brd2,Rasa4,Naa50,Rassf4,Gpr34,Basp1,Arhgdia,Ms4a14,Glmp,Pmaip1,Tifab,Celf2,Tnfaip8l2,Dse,Atp6v0d1,Rela,Cnppd1,Atf4,Slc38a1,Gas2l3,Wasf2,Ebi3,Slc9a3r1,Scamp5,Relb,Tgfbr2,Mfsd11,Ubn1,Scn1b,Csf2rb2,Actr2,H2M3,Grk2,Nolc1,Lpar6,Wipf1,Tanc2,Tagap1,Etf1,Fuca2,Chd7,Parp14,Edem1,Safb2,Gm14221,St3gal5,Cttnbp2nl,Gpr160,Rtf2,Wwp2,Cd28,Arl6ip1,Pdcd6ip,Irak2,Gem,Noct,Rhoh,Snx10,Fcho2,Syngr1,Ang,Ubash3b,Card19,Dhx40,Ifnar2,Psme1,Slc37a2,Pcf11,Cdc42se1,H2T23,Ddx21,Hsd17b12,Ndel1,Pnrc1,Srsf2,Itgb2,Arhgap19,Mdfic,Ifi202b,Ncoa3,Pycard,Gpx1,Ubap2l,Mapre2,Cbl,Atp6v1d,Dusp5,Cap1,Npl,Dok2,Mt2,Itga6,Gnai2,Ctns,Mvp,Grina,Fam105a,Fchsd2,Slc29a3,Zbtb7a,Dok3,Hs6st1,Tnfrsf1a,Hnrnpab,Eif3a,Coro1b,Cryl1,Ccdc115,Stk4,Trafd1,Slc31a1,Klf13,Twf1,Dtnbp1,Rab11fip5,Itga5,Zfp36l1,Blvra,Gls,Tor3a,Sh2b3,Bcl3,Necap2,Cln3,Tap1,Sh3bp2,Etv5,Plekhm2,Slc12a9,Rufy3,Myo7a,Srsf7,Fes,Hcst,Slc38a6,Fcgr4,Fam111a,Itsn2,Lyl1,Nek6,Sp140,Renbp,Trim30a,Zfp710,Pip4k2a,Dusp6,Zfp800,Mob1a,Tmed5,Abca9,Scarb2,Raph1,Tmem243,Abi1,Srgap2,Elf4,Pkn1,Use1,Rap1a,Sema4b,Hpgd,Dip2b,Dbnl,Hmga1,Rabgef1,Stard3nl,Pdgfb,Sipa1,Tmem106a,Hspa5,Wdr1,Tmem206,Os9,Tpbgl,Zfp263,Man1c1,Kcnk6,Rab14,Adipor1,Tm6sf1,N4bp1,Pfkfb3,AC127341.3,Larp4b,Cfh,Ubl3,Eepd1,N4bp2l1,Susd6,Arih2,Hnrnpf,Tmem141,Osbpl8,Frrs1,D1Ertd622e,Pabpc1,Tsc22d2,C2,Tmem9b,Sf1,Snx20,Ywhag,Ccdc86,Orai1,Ppp1r21,Clcn5,Arid5a,Zfhx3,Tcof1,G3bp1,Plpbp,Sh3bp5,P2ry13,Dot1l,Dusp7,Sft2d2,Alox5,Atp1b3,Mapkapk3,Gpsm3,Coro1c,Atp6v1c1,Pcyt1a,Herpud1,Kcnk13,Acly,Rac1,Lrmp,Socs3,Pepd,Qk,Osbpl11,Cd63,Dnajc13,Itgal,Pdlim4,Sppl2a,Slc30a1,Incenp,Tpst2,Adgre5,Tspan33,Ldlr,Snx18,Elovl1,5430427O19Rik,Themis2,Cdt1,Arhgap18,Tmcc3,Myd88,Ikbke,Atp6v1g1,Tmbim1,Abl2,Relt,0610012G03Rik,Ell2,Adcy7,Cdc42,Timp2,Cmtm6,Abracl,Jak1,Kdm7a,Zdhhc14,Akirin1,Specc1,Psmb10,Paox,Rffl,Fgd4,Rhbdf2,H2DMa,Il16,Dock8,Casp1,Cp,Itgb5,Taok3,Vav3,5031439G07Rik,Chd4,Elf1,Kpnb1,Ube2f,Dnm2,B4galt6,Nfxl1,Per1,Arf3,Gba,Selplg,Kansl1,Nagpa,Reep5,Naga,Kdm2b,Adam9,Fkbp15,Aftph,Clint1,AB124611,Adam33,Bcor,Nhp2,Papd4,Itpkb,Slc15a4,Slc11a2,Fem1c,Vps35,2810474O19Rik,Mknk2,Gch1,Ggnbp2,Zswim8,Slc38a7,Pik3cg,Mcm3,Cnpy3,Sec24a,Sdf2l1,Msmo1,Wdr81,Lrp12,Srd5a3,Med21,Camk1d,Lpxn,Cd79b,Ap2m1,Sp110,Reep3,Stat3,Hnrnpu,Man2a1,Cks2,Arhgef2,Ssfa2,Plgrkt,Lncpint,Rnpep,Wbp2,Stxbp2,Csk,Klf7,Cdk6,Psmb9,Spag9,Dtx4,Milr1,Prex1,Adap2,Smcr8,Hps3,Msrb1,2510039O18Rik,Dclre1c,Dmxl1,Prpf40a,Akr1b3,Rnf213,Gnaq,Tbc1d8,Lrp6,Cab39,Arl4d,Rrad,Fnip2,Brpf1,Tmem50a,Nfatc1,Ppt1,Derl1,Ten1,Tmem256,Rassf2,Clcn7,Tab2,Heatr1,Got1,Prdm1,Atp13a3,Tgs1,Vapa,Lsp1,Ranbp2,Rnf166,Atp6v1e1,

Cluster18

Cxcl2,Ccl4,Ccl3,Tnf,Ccl2,Ccl7,Cd83,Pf4,Dusp2,Slfn2,Bcl2a1b,Ifi207,Pim1,Nfkbia,C1qa,Nlrp3,C1qb,C1qc,Apoe,Ifrd1,Fcer1g,Klf6,Ctss,Neat1,Atf3,Nfkbiz,Lyz2,Tyrobp,Spi1,Mrc1,Mt1,Srgn,Sdc4,Nfkbid,H2K1,Ier3,Laptm5,Lgmn,Hmox1,Cebpb,Csf1r,Ctsb,Ccl12,Zfp36,Plek,Ehd1,Dab2,Irf1,Ltc4s,Grn,Marcksl1,Lyve1,Fcrls,Ucp2,Fcgr3,Ctsd,Trf,Ctsc,Ms4a7,Plin2,Unc93b1,Mcl1,Cyba,Gadd45b,Ier5,H2D1,Ftl1,B2m,Jun,Ctsz,Arpc1b,Rgs10,Junb,Ier2,Jund,Tmsb4x,Ptpn18,Lamp1,Fth1,Ubc,Actb,Cst3,Eif4a1,Clta,Ddx5,Cltc,Vmp1,Gm26917,Selenop,Mafg,Nr3c1,Cited2,Zcchc6,Ap2s1,Serpinb6a,Ifnar2,Jmjd1c,Mat2a,Ddx3x,Sys1,Rps29,Kras,Tmem256,Fcho2,Ifngr2,Use1,Ptbp3,Cdk9,Etf1,Higd2a,Nhp2,Spg21,Cd63,Kansl1,Gnl3,Brk1,Tiparp,Asah1,Gm47283,Cdk12,Vamp8,Nol7,Gm26870,Capzb,Psme2,Sbno1,Tpp1,Wnk1,Ndel1,Bach1,Erbin,Ctsh,Dnajb1,Jpt1,Ptp4a1,

Cluster30:

Cryab,Cryaa,Crybb3,Dapl1,Col9a1,Crhbp,Mgarp,Gja8,Crybb2,Crygs,Cryba2,Cryba1,Folr1,Apoe,Crybb1,Tkt,Optc,Gstm1,Pax6,Mab21l2,R3hdml,Aldh1a1,Crim1,Pcp4,Tyrobp,Cp,Rgs10,Lyz2,Clu,Hpgd,C1qb,Grifin,Slc7a2,Blvrb,Gja1,Nt5dc2,Fcer1g,Sapcd1,Phgdh,Trem2,Hmx1,Fcgr3,NEdotin3,Col9a3,AY036118,Lgmn,Sfrp1,Aif1,Clta,Ifi27l2a,Dkk3,Hspb2,Ptpn18,Rcbtb2,C1qa,Eno1,Hpgds,Tmsb4x,Gmfg,Gatm,Ccl24,Ms4a7,Alad,Nudt4,Snx2,Ass1,C1qc,Maf,Cadm1,Lgr4,Mrc1,Olfml3,Pf4,Sirpa,Atp1b3,Ctsl,Ccl12,Fcgr2b,Ezr,Fth1,Bst2,Ehd1,Idh2,Atp1a1,Gm42418,Dab2,Nupr1,Ltc4s,Ms4a6c,Prdx5,Selenop,Ctss,Laptm5,Ctsd,Ly6e,Cotl1,Unc93b1,Htra1,Erp29,Ctsb,Rnf7,Ftl1,Txnip,Stab1,Ctsc,Atp6v0e,Chchd10,Cyba,Cst3,Mif,Gng5,Ptov1,Tsc22d1,Cdkn1c,Spi1,Bsg,Ctsz,Ckb,Ccl2,Pfdn5,Arpc3,Ccl7,Gapdh,Gabarap,mt-Co3,Tpt1,mt-Co2,Vim,Rpl14,Prdx1,Actg1,Ndufa4,Hnrnpk,Cd63,mt-Nd2,

Erythroid-like and erythroid precursor cells

Cluster16
Hbaa1,Hbaa2,Hbbbs,Hbbbt,Alas2,Snca,Mkrn1,Bpgm,Gm26870,Gabarapl2,Cenpb,Gpx1,

Cluster19
Hbbbt,Hbaa2,Hbbbs,Hbaa1,Alas2,Snca,Mkrn1,Bpgm,Ube2l6,Fech,Fam220a,Fam46c,Slc25a37,Isg20,Gabarapl2,Epb41,Slc25a39,Gpx1,Rsad2,Cd24a,Ube2o,Bnip3l,Ncoa4,Ccndbp1,Pnpo,Bola3,Ube2c,Isca1,Ppp1cb,Fam213a,Hagh,Sec61g,Cdr2,Ghitm,Rnf10,Slc48a1,Riok3,Cmas,

Cluster23
Hbbbs,Hbaa1,Hbaa2,Hbbbt,Alas2,Cryab,Cryaa,Crybb3,Bpgm,Snca,Mkrn1,Ube2l6,Crybb2,Fam220a,Fam46c,Dapl1,Cryba2,Mgarp,Col9a1,Gja8,Crygs,Crhbp,Fech,Slc25a37,Gstm1,Folr1,R3hdml,Isg20,Tkt,Sapcd1,Sfrp1,Aldh1a1,Cryba1,Clu,Slc25a39,Optc,Pcp4,Cd24a,Car2,Crim1,Ncoa4,Pax6,Phgdh,Cadm1,Gabarapl2,Epb41,Nectin3,Mab21l2,Ccndbp1,Cp,Dkk3,Eno1,Gja1,Nudt4,AY036118

Cluster25
Hbbbt,Hbbbs,Hbaa1,Hbaa2,Snca,Alas2,Mkrn1,Gpx1,Bpgm,Car2,Ube2l6,Isg20,Gypa,Pnpo,Cd24a,Slc4a1,Fam46c,Fech,Fam220a,Slc25a37,Fam213a,Blvrb,Rsad2,Isg15,Prdx2,Tspo2,Ube2o,Gabarapl2,Cmas,Rad23a,Mpp1,Hagh,Ccndbp1,Ube2c,Epb41,Slc25a39,1810058I24Rik,Rnf10,Cdr2,Ube2b,Ncoa4,E2f2,Ghitm,Bnip3l,Gclm,Ucp2,Trim10,Tfdp2,Slc48a1,Fam117a,Hscb,Glrx5,Sec61g,Ctse,Cdc25b,Tspan33,Ppp1cb,Dhrs11,Fam210b,Hbq1b,Arf5,Riok3,Pla2g16,Grina,Creg1,Hist1h1c,Cat,Isca1,Urod,Yipf4,Adipor1,Ptp4a3,Psme3,Nt5c3,Gadd45a,Taldo1,Bola3,Dcaf12,Rec114,Bcl2l1,Ubb,Smox,Rbm38,Ube2h,Fam126a,Txnrd2,Epb42,Slc25a51,Ybx3,Oaz1,Iscu,St3gal5,Eif5,Map1lc3b,Fbxo9,

Neutrophils:
Cluster22
S100a9,S100a8,Stfa1,BC100530,Gm5483,Stfa2l1,Plac8,Retnlg,Il1b,Stfa2,Lcn2,Cd52,Lyz2,Stfa3,

Coro1a,Lgals3,Srgn,Cebpb,Alox5ap,Hp,Cybb,Gsr,Msrb1,Cytip,Ptprc,2010005H15Rik,Itga4,Napsa,S100a6,Tyrobp,Cd44,Pou2f2,Lsp1,Thbs1,Cdk2ap2,Ifitm6,Fcer1g,H2D1,Ccl6,Fyb,Neat1,Itgal,Lcp1,Spi1,S100a4,Iqgap1,Pglyrp1,Treml4,Adgre5,Pim1,Cxcl2,Laptm5,Rnf149,Igsf6,Myo1g,Cd14,Mcemp1,Itgb2,Slc7a11,Tgm2,Samhd1,F10,Fxyd5,Plaur,Cyba,Rac2,Cd300c2,Gngt2,Tnfaip2,Apbb1ip,Samsn1,Stk17b,Nfam1,Ccrl2,Ltb4r1,Arhgap30,Stk10,Ptpn6,Fgr,Gm2a,Ace,Cd53,Lyn,Ly6e,Ncf4,Slfn2,S100a11,Snx20,Gpr141,Cd300a,Rap1b,Dusp5,Arhgdib,Trem1,Prr13,Plin2,Ptpn1,Ptpre,Bcl2a1b,Lst1,Anxa1,Fam49b,Mcl1,Nfkbia,Il17ra,Msn,Myo1f,Metrnl,Ifngr1,Dusp16,Grk3,Nadk,Cd47,Csf2ra,Prkcd,Pla2g7,Fmnl1,Ctss,Sirpa,Tpd52,Cyp4f18,2810474O19Rik,Mpeg1,H2K1,Fcgr4,Ncf2,Flna,Rgs2,Ikzf1,Tnfrsf1b,Clec4e,Pilra,Dusp2,Tspo,Spn,Nfe2l2,Hck,Zyx,Il10ra,Hcls1,Ear2,Ptafr,Emb,Cd83,Klf2,Rassf5,Pld4,Ankrd44,Itgam,Smpdl3b,Ets2,B2m,Btg1,Slc16a3,Adgre4,Bcl2a1a,Taldo1,Psap,Clec4a3,Fpr2,Malt1,Efhd2,Kdm6b,Emilin2,Csf1r,Pde4b,Cxcr4,Cyfip2,Csf2rb,Limd2,Arpc1b,Dok3,Vsir,Sh3bgrl3,Cks2,Sema4d,Trem3,Lrrfip1,6430548M08Rik,Gpr132,Unc119,Nr4a1,Pmaip1,Cd9,Prdx5,Plek,Tlr2,Bcl2a1d,Irf5,Crip1,Cd36,Rel,Gpcpd1,Pag1,Cd300ld,Smpdl3a,Grk2,Cdc42ep2,Pygl,Eno3,Gpx1,AB124611,Rnase6,Rap1a,Plbd1,Gpsm3,Cd300lf,Klf13,Il6ra,Tmed5,Lcp2,Cst3,N4bp1,Ehbp1l1,Sorl1,Gmfg,Slc12a2,Cd48,Nuak2,Man2b1,Lilr4b,Ikbkb,Actr3,Ssh2,Taok3,Slc11a1,C5ar1,Arhgap45,Lyst,Nabp1,Ostf1,Arid3a,Rhog,Grb2,Clec4a1,Dennd4a,Slc15a3,Celf2,Sirpb1c,Syk,Arhgap15,Ptprj,Grina,B4galnt1,Myl12b,Shisa5,Zfp36l2,Herc4,Cd82,Arpc2,Emp3,Diaph1,Pip4k2a,Zfp36,Fth1,Klf3,Rasa3,Fam49a,Nrros,Sod2,Zeb2,N4bp2l1,Rilpl2,Naga,Agpat4,Ahnak,Nupr1,Pfn1,Cyth4,Ywhaz,Dock2,Raf1,Arl4c,Tln1,Stk4,Fgd4,Dusp1,Ndel1,Adcy7,Notch2,Ptbp3,Camk2d,Ppp2r5a,Sppl2a,Klf6,Ms4a6c,Ccnd3,Syngr2,Riok3,Fam107b,Mapkapk2,Fermt3,Irf1,Lnpep,Eif4ebp1,Crlf2,Psmb8,Svil,Tnfaip8,Nab1,Baz1a,Trib1,Cnn2,Stk38,Nckap1l,Pgd,Glud1,Lrrc25,Zc3hav1,Kdm7a,Stk24,Arhgef1,Actb,Cd68,Mrpl33,Lpcat2,Hectd1,Plekho2,Cbfa2t3,Tmsb4x,Atp6v0b,Ptp4a1,Anp32a,Actg1,Dazap2,Ier5,Kmt5a,Ezr,Ccnl1,Cotl1,Klf4,Prpf38b,Akap13,Picalm,Mob1a,Ifitm3,Ifitm2,Fes,Gfpt1,Vasp,Fam96a,Atp2b1,Mef2a,Tgif1,Dbnl,Skap2,Slk,Tpr,Add3,Pkn1,Trps1,Atp1a1,Ucp2,Prex1,Fosl2,Clic1,Mgst1,Cbl,Tkt,Capza1,Tm6sf1,Aprt,Capzb,Crlf3,Kpna4,Clip1,Actr2,Bach1,Arhgap17,Rbpms,St3gal5,Adipor1,Bhlhe40,Mbnl1,Sem1,Bri3bp,Cap1,Fam32a,Capza2,Atp6v0e,Atf4,Cmtm7,Myh9,Nin,Pan3,Smc6,Tgfb1,Ctsb,Ppp2r5c,Plec,Ptpn18,Hmgb2,Tbpl1,Vamp8,Ak2,Ppp1r12a,Atp1b3,Rara,Wsb1,Zcchc6,Rsrp1,Cdkn1b,Atp6v1e1,Tet2,Mia2,Nsd3,Tgfbi,Nfkb1,Larp4b,Sgk1,Cdc42ep3,Birc6,Pak2,Csk,Fau,H3f3a,Litaf,Twf2,Arpc3,Vps37b,Arpc4,Ddx5,Gusb,Btg2,Orai1,Creg1,Tgfbr1,Tpm3,Serp1,Sf3b1,Nufip2,Junb,Scand1,Slc38a1,Lars2,Cx3cr1,Arpc5,Emd,Rhoa,Rab8a,Cd302,Evl,Tapbp,Mark2,Cmip,Pabpc1,Lmo4,Zbtb7a,Eif5a,Rnh1,Rasgrp2,Ifrd1,Calm1,D8Ertd738e,Pkm,Gm26532,Sec11c,Atp6v1b2,Usp25,Tor1aip1,Gnb2,9-Sep,Anp32b,

Astrocytes

Cluster27

Gfap,Dbi,Cst3,Gpm6b,Ckb,Slc1a3,H19,Igfbp2,Nnat,Aldoc,C1ql1,Crip1,Syt11,Cp,Sct,Kcnmb4,Sox9,Fbxo2,Optc,Sorbs2,Ddah1,Col2a1,Hes5,Basp1,C1ql3,Mlc1,Pax2,Cd9,Ass1,Rlbp1,Adamts16,Ldha,Metrn,Thrsp,Sox2,Ntrk2,Pdlim3,Pdgfra,Acot1,Vegfa,Pak3,Ppa1,Lhx2,Atp1b1,Cntn1,Slc6a11,Bag1,Fos,Tspan3,Mgarp,Fn1,Cd24a,Espn,Mxra7,Col9a1,Cyr61,Spry1,Shisa8,Tpi1,Vim,Igfbp5,Socs3,Mmd2,Hspa4l,Kcne1l,Plpp3,S100a10,Rtn1,Bcan,Eno1,Tubb2b,Six3,Slc27a1,S100b,Mfap2,Mrpl13,Bsg,Ndrg2,Prdx6,Nav1,Slc22a17,Gadd45g,Wfdc2,Ddr1,Tes,Hopx,Tuba1a,Ywhah,Dclk1,Fxyd6,Tgfb2,Dap,Prpsap1,Id2,Dkk3,Hist3h2ba,Spon1,Tsc22d4,Nav2,Vit,Scrn1,Nr2e1,P

pp1r1a,Agrn,Gstm5,Fez1,Col9a3,Ndn,Slc30a10,Dhx32,Nfix,Pbx1,Retreg1,Fabp7,Rax,Jam3,Phactr1,AC154683.1,Sphk1,Fam19a5,Vstm2b,Ppp1r14b,Btbd17,Ndufc2,Ttyh1,Fosb,Fbln2,Scg5,Mpped2,Id4,Selenow,Creb5,Jun,Nfib,Crim1,Angpt2,Prelp,Me1,Ccser2,Pebp1,Myh14,Pcsk1n,Cnmd,Cspg5,Fst,Rcn1,Lcat,Slc38a5,Six6,Clec18a,Atp1a2,Gfra1,Tceal8,Dab1,Cd81,Aif1l,Tagln,Pdha1,Etv1,Ccnd2,Fut9,Zic2,A2m,Kcnmb4os2,Wwc1,Cmtm5,Abhd4,Opcml,Pfkl,Vax2os,Ezr,Pmp22,Id1,Tns3,Ddah2,Lmo4,Npnt,Lmo3,Plpp1,Pygb,Zic5,Epas1,Slc6a6,Tesc,Qk,Btg2,Npas3,Cntnap2,Lman1,Etv5,Mpst,Igf2,Cdh13,Trpm3,Mageh1,Tmem121,Ywhae,Ttyh3,Taf13,Ngfr,Slc16a2,Bhlhe41,Zic1,Dhrs7,Ssbp3,Fbxo44,Pdpn,Trim2,Ctgf,Cxcl12,Scd2,Ninj1,Actg1,Aldoa,Appl2,Sorcs1,Ppfibp2,Pcp4l1,Atp9a,0610040J01Rik,Ptov1,Fzd8,Chadl,Bex2,Tst,Drd4,Lrrtm3,Ildr2,Cdh6,Fgfrl1,Pax8,Ncam1,Odc1,Zcchc18,Celsr2,Pdlim5,Ctnnbip1,Trp53i13,Maged1,2900026A02Rik,Tceal3,H2afy2,Tmem63b,Pkm,Kif5c,Diras2,Fabp5,Brinp1,Ap1s2,Nt5dc2,Aamdc,Cbr3,Samd14,1810010H24Rik,Lrrc8b,Sdk2,Celf2,Hmgn3,Mid1ip1,Lsamp,Pdzrn3,Tmod2,Chrna3,Calr,Fzd9,2610524H06Rik,Luzp2,Ltbp3,Map4k4,Nedd9,Ifitm10,mt-
Co1,Prr7,Morf4l2,Mgst1,1810037I17Rik,Sox5,F3,Slc4a4,Sntb1,Gad2,Apba2,Trim24,Mapk3,Sox11,Ctsd,Kctd14,Abat,Mphosph6,Serf1,Cxxc5,Lrrtm1,Gde1,Wasf3,Hsp90ab1,Arl3,Daam1,Prnp,Ccdc47,Pgk1,Atp6v0e2,Mlec,Pam,Eci2,Arhgef40,Rtn3,Slc15a2,Atp1a1,Ahi1,Dock7,Fam168a,Gapdh,Cldn19,Pmm1,Pcdh9,Spcs2,Mt3,Syndig1,Npdc1,Haghl,Atxn10,Stat3,Clrn1,Map2,Kit,Prmt1,Ldhb,mt-
Nd1,Rmst,Kcnma1,Traf4,Zfp593,Adora1,Greb1,Eid1,Tbx3,Efna5,Pgls,Bex3,Arl4c,Gpx4,Csrp1,Taldo1,Ggh,Tulp3,Crlf3,Agt,Trib2,Ptprs,Egr1,Uchl1,Stox2,Prdx2,Intu,Selenof,Bhlhe22,Pafah1b3,Tmem37,Creg2,Timm13,Fzd3,Hsp90aa1,Olfm2,Srp14,Pfn2,Hsbp1,Lmcd1,Sys1,Kitl,Ncbp2,Crip2,Hspa5,Lrrc4b,Plppr1,Naaa,Prdx4,mt-
Co2,Sfrp2,Dbp,Fxyd1,Gsta4,Hddc2,Ift57,Stub1,Vwc2,Cnn3,Tmtc2,Spire1,Wnk2,Manf,Ckap4,Sox3,Igsf3,Spry2,Phgdh,Snap47,Rdh10,Epha4,Sema5a,Cotl1,Naxd,Serpinf1,mt-
Co3,Kif21a,Ndufa11,Sall1,Dad1,Atraid,Pcnp,Nlgn3,Galk1,Tspan7,Hspa13,Slc25a4,Gm30191,Kcnd3,Maff,Smim7,Mettl9,Kcnq1ot1,Larp1b,Tpd52l1,Plod2,Dlc1,Astn1,Lrpap1,Pdia6,Rhoq,Hkdc1,Mgst2,Vcl,Vdac1,4930402H24Rik,Hpcal1,Ndufs4,Crot,Epha5,Mpc2,Slc6a1,8-
Sep,Arl2,Ascl1,Timm8b,Asap2,Ergic3,Oxa1l,Sall2,Pacsin2,Ube2m,Comt,Higd1a,Tceal9,Ndufc1,Mpc1,Ssbp2,Pigyl,Pgam1,Arl8a,Dbn1,0610009B22Rik,E130114P18Rik,Fmn2,Smarcd3,Ufl1,Sdf2,Mrps6,Slc35b2,Dpysl3,Sema6d,Hcn2,Gnb2,Svbp,Ncs1,Hes1,Cnpy2,Adgrg6,Cfap20,Ywhaz,1110065P20Rik,Mab21l2,Ptprz1,Slc14a1,Apex1,Chpt1,Pbxip1,Hacd1,Pkdcc,Sox8,Cox20,Kcnk1,Ift27,Eif1ax,Trak1,Cd276,Kif3a,Cdk2ap1,Emc10,Ddost,Slc12a4,Pepd,Nfia,Tia1,Suco,Vax2,Tubb4b,Abca2,Dlgap1,Znrf3,Calm3,Nxf1,Pdlim4,Camk2d,mt-
Atp6,Pnmal2,Smarcb1,Ppp1r9a,Evi5,Carnmt1,Magt1,Prkca,Dnaja1,Fut8,Kbtbd11,Zfyve21,Carhsp1,Dnajb11,Ttc3,Park7,Atxn7l3b,Bcat1,Ywhaq,Grina,Glt8d1,Mcc,Fam234a,Tbcb,Lpar6,Mocs2,Limd1,Bccip,Cdon,Antxr1,Mad2l2,Tnik,Mmp14,Tmem208,Ptprj,Atp5g2,Dnajc1,Shisa2,Cfl1,Ctdspl,Cadm1,Slc26a7,Plekhf2,Nos1ap,Plec,Mrpl28,Bbip1,Usp24,Nudt19,Ufc1,Fkbp2,Rheb,Shisa4,Tob1,Edf1,Pitpnc1,A730017C20Rik,Tspan15,Glo1,Lamtor4,Cux1,Mapre1,Anapc13,Pcgf5,Cyp7b1,Slc25a23,Tox3,Etv4,Hook1,Gpr153,St3gal6,Ndufab1,Aurkaip1,Vldlr,Ift22,Sbno2,Ndufa13,mt-
Nd4,Pigp,Gxylt2,Dnajc15,Uqcc2,Tspan12,Fam181b,Negr1,Puf60,Kif1b,Calm2,Nrcam,Smim11,Mrpl17,BC031181,Nkain4,Txndc15,Shtn1,Cisd1,Galnt7,Reep2,Lama5,Pmpcb,Tiam1,Fam173a,Cdc37l1,H1fx,Wipi1,Suclg1,Commd1,Psph,Bex4,Mrpl12,Abhd16a,Rasal2,1700025G04Rik,Emc6,Maml2,Sec11c,Tspan31,Pea15a,Cers6,Trappc4,mt-

Cytb,Zfp219,Agpat5,Slc35f1,Tecr,Ccdc136,Cfap36,Adh5,Ikzf2,Ndufa12,Mettl26,Gamt,Fbxl15,Bcap31,Ccdc90b,Ier2,Snrnp27,Eef1g,Polr3h,2410015M20Rik,Jkamp,Kcna6,Tmem147,Gtf2i,Tmem14a,Flot1,Mfsd6,Alg5,Fam110a,Tmx4,Sdhaf4,Map3k1,Smpdl3b,Fnta,Exoc7,Smc6,Mdga1,Pcdh7,6330403K07Rik,Trib1,Srsf9,Elovl6,Copb2,Hes6,Casc4,Gpi1,Ace,Tmed1,Dgcr6,Ppp1ca,Hmgn1,Mboat2,Dynll1,Hey2,Nr2f1,Zbtb18,Gldc,Ankrd40,Rcn2,Selenbp1,Gabbr1,Stx3,Spred3,Pabpn1,Sumf2,Dlg4,Scrib,Neo1,Slc39a7,Rab28,Cstf3,Fermt2,Ranbp1,Eef1akmt1,Ak2,Ndufb10,Ndst3,Ctnnd2,Iah1,Tnfrsf12a,Irgm1,Nacc2,Lrrc49,Kremen1,Ndufv2,Erp29,Fam120a,Rnf145,Epb41l5,Mdh2,Yif1a,Hs2st1,Sumo2,Snrpd2,Mrps21,Apopt1,4933431E20Rik,Cbs,Hadh,Notch1,Slc5a3,Srgap3,Zfp704,Rsf1,Ube2n,Psmd7,Copg1,Atp5o.1,Ctsl,Sfxn5,Dag1,Hspa8,Atp5g1,Clcn5,Cuedc1,Arntl,Megf6,Tlcd1,Arhgap21,Egr2,Srebf1,Cd151,Panx1,Rnaset2b,

Cluster29

Dbi,Ckb,Gfap,H19,Cst3,Nnat,Aldoc,Metrn,Crip1,C1ql1,Basp1,Rlbp1,Ldha,Tuba1a,Tubb2b,Gpm6b,Igfbp2,Eno1,Ass1,Vim,Prdx6,Ddah1,Mgarp,S100a10,Pkm,Prdx2,Gapdh,Selenow,Gnb2,Gm42418,Tubb5,Actg1,Slc25a4,Lamtor4,Hsp90ab1,Pdap1,Pdcd6,Ctbp1,Ctnnbip1,Ube2m,Rbbp7,Tcf3,Rbm3,Ube2l3,Malsu1,Phf5a,Ssbp4,Dnajc1,Nfic,Pbx1,Hspa8,Tmsb10,Cul1,Lamtor2,Srsf7,Ndufb8,Metap2,Eif4a3,Mpc2,Dld,Smim10l1,H3f3a,Wbp4,Mcrs1,Rpsa,Cuedc2,Yif1b,Snrpc,Cdc37,Vamp3,Suclg1,Fkbp4,Srp72,Reep5,Ndufb3,Smarca5,Anapc11,Rpl7,Mbd3,Psmd13,Eif2a,Qk,Rnf187,Yeats4,Wipi1,Lsm8,Ogfr,Dnajc5,Mettl9,Polr2e,Zfhx3,Snrpd3,Actl6a,Trp53,Rad21,Med10,Lman1,Safb,Saraf,H2afj,Thoc7,Srp19,Rplp0,2410015M20Rik,Ube2q1,Tmem208,Mrpl54,Rpl41,Ppia,Nop10,Ccdc34,Psmb7,Pfdn2,Sgta,Scaf11,Cops9,Larp4b,Timm17a,Ift20,Trim28,Ranbp1,Tpp1,Strn3,Dag1,Srpk2,Acadl,Plgrkt,Ppm1a,Pcbp1,Eif4e,Hspa9,Txndc9,Rala,Rtn4,Psmd6,Cox14,Coq7,Scand1,Serinc1,Chmp1a,Nudt21,Ap3s1,Tmem160,Gatad2b,Sdf4,Gng12,R3hdm2,Bub3,Tmem258,Mkln1,Edf1,Rab1b,Tsen34,Arhgap5,Ncor2,Psmc5,Sap30l,Trappc6b,Lmo4,N4bp2l2,Ost4,Cnih4,Anp32b,Sec31a,Necap2,Cdc5l,Ssu72,1810058I24Rik,Sde2,Chchd1,Papola,Atxn10,Tomm7,Fkbp3,Naxe,Eif3d,Banf1,Rps9,

Novel: undfined Immuno-related cells

Cluster26

Hist1h2ap (T cells)/Neutrophils),Top2a (Erythroid precursors/B cell),Hmgb2 (Erythroid precusor/B cell),Hist1h1b (neutrophil),Smc2 (Erythroid),Cenpa(Erythroid precursor),Stmn1 (Bcells) ,Mki67 (Erythroid precursor/Tcells),Birc5 ((Erythroid precursor),Tuba1b,H2afz,Cdk1,Tubb5,Cenpf,Pclaf,Prc1,Col1a2,Cdca3,Cenpe,Ube2c,Spc24,Hmgn2,Cks2,Peg3,Nusap1,Cdca8,H2afx,Hist1h1e,Hist1h2ae,Col3a1,Hjurp,Tmpo,Hmmr,Kif23,Smc4,Tubb4b,Col1a1,Spc25,Ccnb1,Racgap1,H2afv,Fn1,Tpx2,Nucks1,Eln,Incenp,Crym,Dcn,Atad2,Cdc20,Aurkb,Kif11,Hmgb1,Ckap2,Neil3,Ccnb2,Ccdc34,Cks1b,Arl6ip1,Esco2,Fbxo5,Fxyd6,Mdk,Rad21,Lockd,Gas1,Knl1,Lgals1,Dek,Gdf10,Clspn,Prdx4,Igfbp5,Nfix,Dtl,Usp1,Mest,Fst,H1fx,Pbk,Selenoh,Plagl1,Dpysl3,Tacc3,H19,Mis18bp1,Hist1h4d,Lox,Pitx2,Xist,Lum,Lmnb1,Tk1,Ube2s,Psip1,Cdh11,Col5a2,Kif20b,Nfib,Ncapd2,Cit,Kif15,Ckap4,Fbln1,G2e3,Ckap2l,Bgn,Foxd1,Hist1h2ab,Dbf4,Cenpm,Dnajc9,Pimreg,Postn,Rangap1,Anp32e,Tfap2b,Nasp,Depdc1a,Sgo2a,Kif22,Col6a3,Cped1,Cenph,Ccna2,Knstrn,Fbn2,Pdgfra,Gpc3,Nsd2,Rbp1,Aspm,Gas2l3,Nr2f2,Tenm3,Cenpw,Nkd2,Snrpd1,Col6a1,Rad51ap1,Jpt1,Tyms,Tpm1,Col6a2,Sulf2,Bub3,Dut,Rrm1,Slbp,Mgp,Tgfbi,Ncapg2,Plk4,Topbp1,Twist1,Dlgap5,Kazald1,Mfap4,Rnf26,Ndc80,H1f0,Cyp26a1,Li

g1,Mxd3,Cenpn,Ncapd3,Ptn,Col8a1,Tcf4,Kif4,Mad2l1,Spats2l,Col5a1,Ncapg,Tipin,Siva1,Pcdh17,Trim59,Hnrnpd,Bub1b,Brd8,Igsf3,Smc6,Dtymk,Foxc1,Emilin1,Spag5,Rbm3,Dnmt1,Cenpk,Zfhx4,Lsm3,Hist1h4i,Ran,H2afy2,Mmp14,Cdkn2c,Mfap2,Shcbp1,Anp32b,Sgo1,Flrt3,Rrm2,Ckap5,Ska1,Ptma,Nuf2,Phip,Col16a1,2810474O19Rik,Hmcn1,Dhfr,Cenpp,Sox11,Ank,Slc43a3,Svep1,Serpinf1,Diaph3,Cbx5,Aurka,Cdca2,Anln,Nfic,Nde1,Cdk5rap2,Ssrp1,Hnrnpr,Col26a1,Rpa2,Myh10,Foxp2,Hmgn1,Fam171b,Foxm1,Hnrnpdl,Ldlr,Baz1b,Rbms3,Smc1a,Nfia,Fdps,Cbx1,Ing1,Nrk,Pmf1,Col12a1,Cdo1,AC160336.1,Dnm3os,Etv4,Uhrf2,Fgfr1,Hdgf,Rpl41,Kifc1,Wee1,Chaf1b,Pcna,Bub1,Clmp,Rnaseh2c,Tpm2,Melk,Loxl1,Tubb6,Srsf7,Hp1bp3,Usp2,Fbln7,Gmds,Smc3,Cntln,Bcl7c,P4hb,Nr2f1,Fkbp3,Igfbp2,Pdgfrb,Banf1,Hnrnpab,Cenpq,P3h3,Suz12,Epyc,Ncaph,Smarcc1,Hmgcs1,Anapc5,Csrp2,Ctcf,Bptf,Lmo7,Zbtb20,Nsmce4a,Arhgap11a,Hsp90b1,Mllt3,Cnih2,Rcn2,Eef1b2,Rad18,Sae1,Srsf10,Uhrf1,Idi1,Mcm4,Fzr1,Terf1,Rplp1,Lmna,Pkmyt1,Gm10076,Olfml3,Sumo2,Haus4,Hnrnpa3,Rcn3,Srebf2,Ranbp1,Insig1,Mpp6,Fbln2,Nop58,Bicc1,Snrpe,Abi3bp,Pdia6,Snrpf,Emp1,Cpxm1,Maz,Hnrnpa0,Plk1,Gmnn,Sncaip,Alyref,Il11ra1,Ppwd1,Txndc5,Tceal9,Pcolce,Hells,Tcf12,Psrc1,Lpar1,Rps5,Hist2h2ac,Hint1,Syce2,Ddx39b,Lsm2,Itga8,Lrig3,Gpsm2,Dach1,Ccdc88a,Boc,Skp2,Rpl36a,Mis18a,Sfrp1,Nek2,Kmt5a,Itih5,Mex3b,Ilf2,Aldh1a2,Ccdc80,Aebp1,Tmem132c,Hist1h1d,Rpl22l1,Calu,Troap,Lsm6,Mcm7,Cdc25c,Hmgb3,Lbr,Ccne1,Rpl23a,Brca1,Rps20,Npm1,Sapcd2,Gxylt2,Rpsa,Rnf182,Polr2a,Nedd4,Smo,Ctdspl2,Sclt1,Kif2c,Smc5,Ssr2,Lhfpl2,Cdca5,Ect2,Srsf2,Cdca4,Rcc1,Txn1,Fubp1,Pnn,Spdl1,Iqgap3,Cenpl,E2f8,Snrpg,Cdk4,Vkorc1,Calcrl,Pbx1,Blm,Socs2,Ptms,Odf2,Matr3,Adamts2,C330027C09Rik,Hdgfl3,Cyp51,Kif20a,Phgdh,Cdc25b,Tra2b,Gpc6,Tcerg1,S100a10,Papss1,Hnrnpa1,Bora,Il33,Itgbl1,Wdr76,Foxp4,Nsg1,Nap1l1,Zfp367,Flrt2,Asxl3,Rfc4,Auts2,Rps12,Zfp101,Emcn,Ccar1,Lsm4,Prim2,Rplp0,Calm2,Hmgn5,Rps15,Thbs2,Hirip3,Brip1,Srsf3,Fstl1,Tnc,Supt16,Rfc3,Mettl9,Irs2,Larp7,Mcm6,Ltbp3,Scube1,Six1,Prrx2,Mtch1,Ppp1cc,Rpl39,Epb41l3,Rpl31,Ptges3,Mmp2,Bzw2,Sdk1,Grb10,Oat,Hmgxb4,Ccnf,Snrpd2,Morf4l2,Dctpp1,Hist1h4h,Stag1,Lin9,Orc6,Mcm3,Mrpl18,Ppih,Sertad4,Lrp1,Rfc1,Hes1,Nav2,Cbx3,Kcnq1ot1,Pafah1b3,Myef2,Fzd1,Tmem167,Palld,Rdx,Rnf168,Pla2g4a,Scarf2,Art4,Naa50,Igf1r,Slc38a2,Ugdh,Hnrnpul1,Pole3,Nktr,Sdc1,Dact3,Fam83d,Ddost,Rps19,Copz2,Hist1h3c,Ankrd11,Nudt21,Ogfrl1,Kdelr2,Tab2,Ltbp1,Sf3b3,Mybl2,Cfdp1,Nup62,Rbms1,Col8a2,Trim28,Lin54,E2f7,Lsm5,Zfp36l2,Fmr1,Hmgcr,Klf5,Timeless,Tcp1,Asf1b,Kpna2,Arid2,Hnrnpa2b1,Crispld1,B230219D22Rik,Mastl,Rps17,H2afy,Exo1,Mcm5,Msmo1,Cygb,Irx1,Cxcl12,Stub1,Brca2,Cdkn1c,Foxn3,Zfp91,Brip1os,Gm47283,Rpl10a,D10Wsu102e,Ctsk,Nudc,Ccng2,Hat1,Ostc,Vars,Tsn,Rbfox2,Colec12,Tuba1c,Hist1h3e,Etaa1,3632451O06Rik,Rbl1,Cep57,Ints6,Snrpb,Rps28,Srsf1,Cep192,Bclaf1,Barx1,Cenpc1,Rps4x,Rhno1,Cdc5l,Tfdp1,Slfn9,Pbx3,Cask,Fuca1,Rpl12,Zfp395,Sass6,Hist1h1a,Med13l,Pmepa1,Dcakd,Fndc1,Prrx1,Cenpu,Cnn3,Trip13,Stil,Rpl18,Maged1,Tcf19,Ube2t,Prpf4,Agtr2,
