# Supplementary material for: Single-Cell Characterization of the Frizzled 5 (Fz5) Mutant Mouse and Human Persistent Fetal Vasculature (PFV)
Source: Invest Ophthalmol Vis Sci. 2023 Mar 3;64(3):8. doi: 10.1167/iovs.64.3.8 (PMC9988703; doi:10.1167/iovs.64.3.8)
Supplement: Supplement 2 [file iovs-64-3-8_s002.pdf]

C5 GO functional gene set

Hprt/Sdhd/Uqcrfs1/Cox6a1/Khk/Aprt/Aldoa/Gpi1/Ndufv3/Ndufv2/Atp5g3/Park7/Ndufa12/Ndufs6/Ndufb9/Bad/Atp5a1/Mif/Ndufs8/Uqcr10/Atp5f1/De/Cdk1/Ndufa7/Cox7a2/Atp5d/Atp5k/Ndufb8/Atp5j/Atp5g1/Cox4i1/Uqcrq/Atp5c1/Atp5j2/Cox5b/Cox5a/Rhoa/Uqcrh/Cox7c/Atp5e/Atp5l/Cox7a2l/Atp5h/Atp5g2/Atpif1/Cmpk1/Ran/Scp2/ Nme1/Mocs2/Nme4/Pgls/Guk1/ Rnaseh2b/Taldo1/ Hint1/Gapdh/ Dctpp1

Glycolysis/Gluconeogenesis/Pentose pathway:

Pgls: 6-phospho-D-glucono-1,5-lactone lactonohydrolase, Pentose pathway
Taldo1: Transaldolase 1, pentose pathway
Aldoa: glycolysis and gluconeogenesis.
Gpi1: glycolysis and gluconeogenesis, as well as the pentose phosphate pathway.
Khk: ketohexokinase, increase conversion of glucose to glucose-6-phosphate
Gapdh: Glyceraldehyde 3-phosphate dehydrogenase, glycolysis

Nucleotide synthesis:

Cmpk1: Uridine monophosphate (UMP)/cytidine monophosphate (CMP) kinase
Nme1/4: The nucleoside diphosphate kinase (NDPK) activity of NME1 is well recognized in balancing the intracellular pools of nucleotide diphosphates and triphosphates to regulate cytoskeletal rearrangement and cell motility, endocytosis, intracellular trafficking, and metastasis. **RhoA/Ran：** Glycolysis, cell movement, DNA replication and cell cycle regression.
HINT1 histidine triad nucleotide binding protein 1.
Hprt: hypoxanthine phosphoribosyltransferase 1, Purine salvage pathway
APRT: Adenine phosphoribosyltransferase, Purine salvage pathway
GUK1: Guanylate kinase 1

Electron transfer chain complex-mitochondria proteins

The rest genes that are not colored.

C30 GO functional gene set

Atp1a2/Chchd10/Nudt4/Uqcrh/Aldoc/Gapdh/Mif/Pkm/Uqcc2/Tpi1/Atp5d/Bpgm/Ndufv2/Atp5b/Atp5c1/Pfkl/Hif1a/Atp5g3/Uqcrb/Ndufc2/Ldha/Stoml2/Atp5j/Mtch2/Galk1/Adss/Ndufs6/Ndufa8/Paics/Impdh2/Surf1/Pmpcb/UqcrfsItpa/Atp5d/Bpgm

Glycosis/Gluconeogenesis

Aldoc: glycolysis and gluconeogenesis.
Gapdh: Glyceraldehyde 3-phosphate dehydrogenase
Pkm Pyruvate kinase catalyzes the last step within glycolysis,
Bpgm Bisphosphoglycerate mutase

Tpi1: Triosephosphate isomerase, glycolysis and gluconeogenesis

Pfkl: 6-phosphofructokinase, function in glycolysis
Galk1: Galactokinase 1, a crucial enzyme to covert galactose to glucose
Ldha, Lactate dehydrogenase A, glycolysis

Nucleotide synthesis
ADSS: adenylosuccinate synthase, de novo purine biogenesis
Paics: Phosphoribosylaminoimidazole carboxylase, de novo purine genesis
Impdh2: Inosine-5'-monophosphate dehydrogenase 2, de novo purine genesis
Itpa: Inosine triphosphate pyrophosphatase, hydrolyzes inosine triphosphate and deoxyinosine triphosphate to the monophosphate nucleotide and diphosphate

Electron transfer chain complex-mitochondria proteins
The rest genes that are not colored.
