# Supplementary material for: Single-Cell Characterization of the Frizzled 5 (Fz5) Mutant Mouse and Human Persistent Fetal Vasculature (PFV)
Source: Invest Ophthalmol Vis Sci. 2023 Mar 3;64(3):8. doi: 10.1167/iovs.64.3.8 (PMC9988703; doi:10.1167/iovs.64.3.8)
Supplement: Supplement 3 [file iovs-64-3-8_s003.pdf]

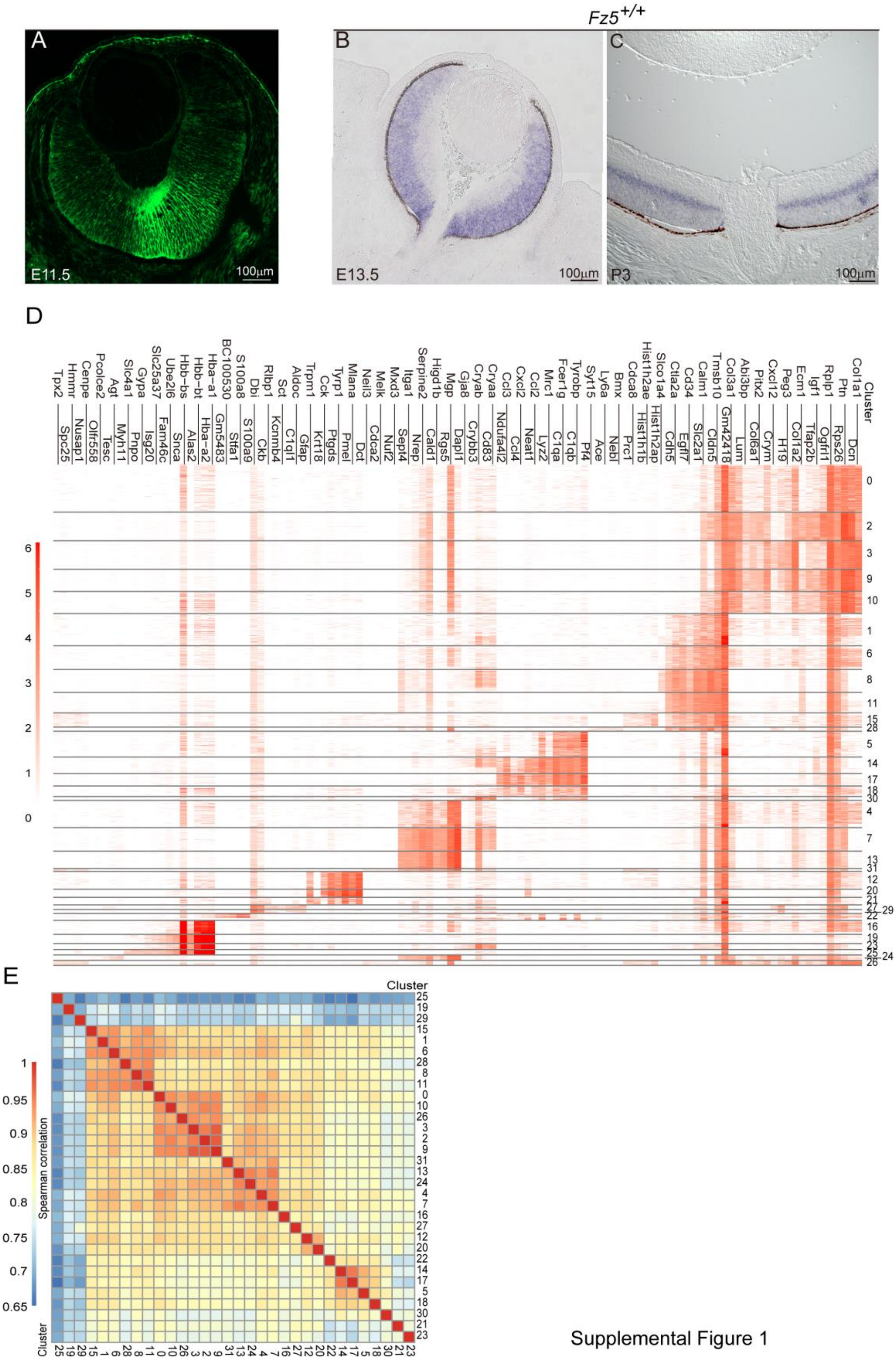

Supplemental Figure 1

Supplemental Figure 1. *Fz5* is not expressed in the vitreous hyaloid vessel cells.

(**A**), *Fz5* expression revealed by a knock-in alkaline phosphatase (AP) reporter stained

with an anti-AP antibody. (**B**, **C**), *In situ* hybridization of *Fz5* mRNA using an anti-sense probe. (**D**), A total of 32 clusters identified by sc-RNAseq expressing the top 20 genes. (**E**), Spearman correlation analysis of the 32 clusters

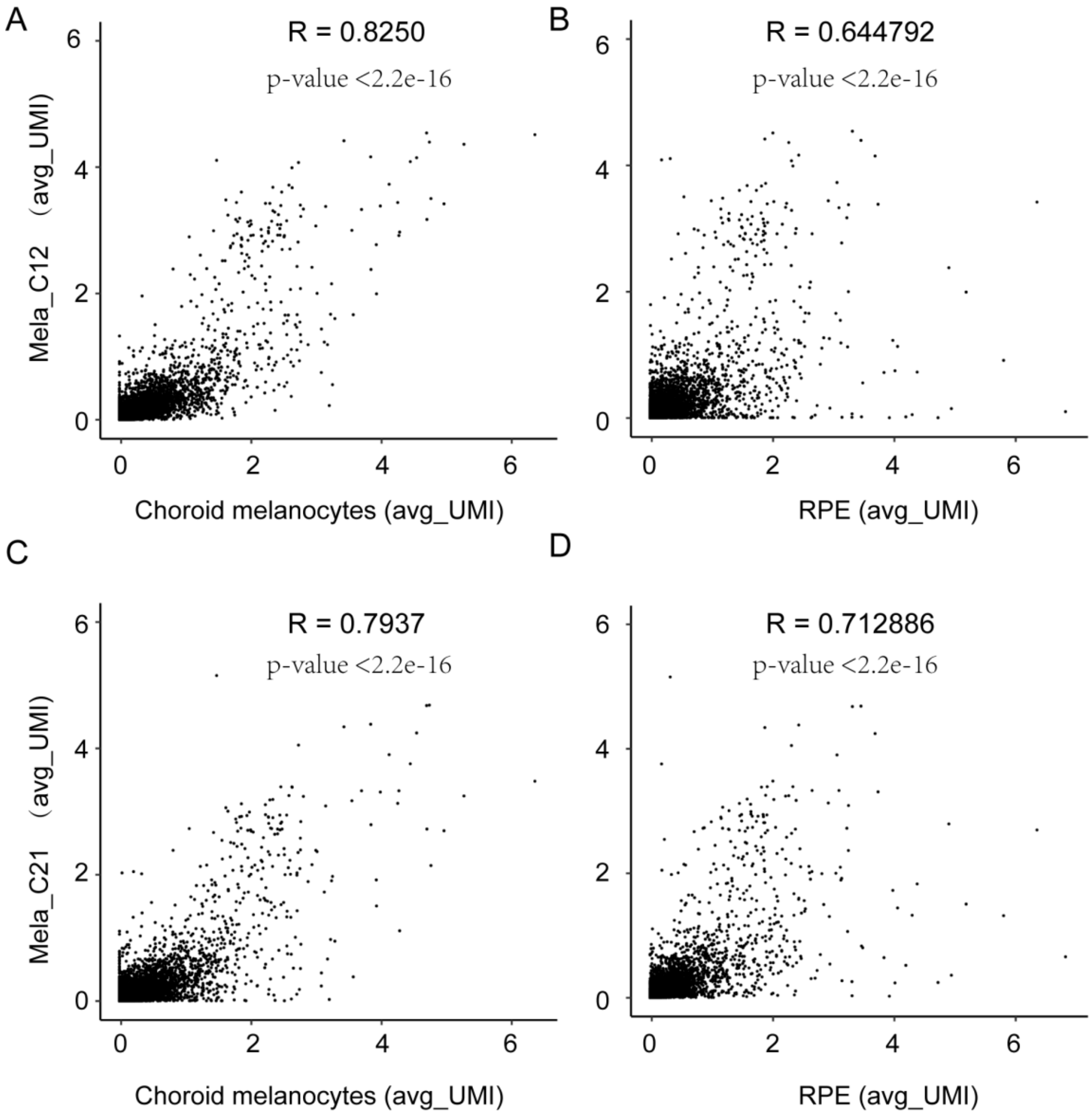

Supplemental Figure 2

Supplemental Figure 2. Pearson's correlation of melanocyte C12 and C21 with the choroid melanocytes and RPE pigment cells. (**A**), C12 with choroid melanocytes. (**B**), C12 with RPE. (**C**), C21 with choroid melanocytes. (**D**), C12 with choroid RPE.

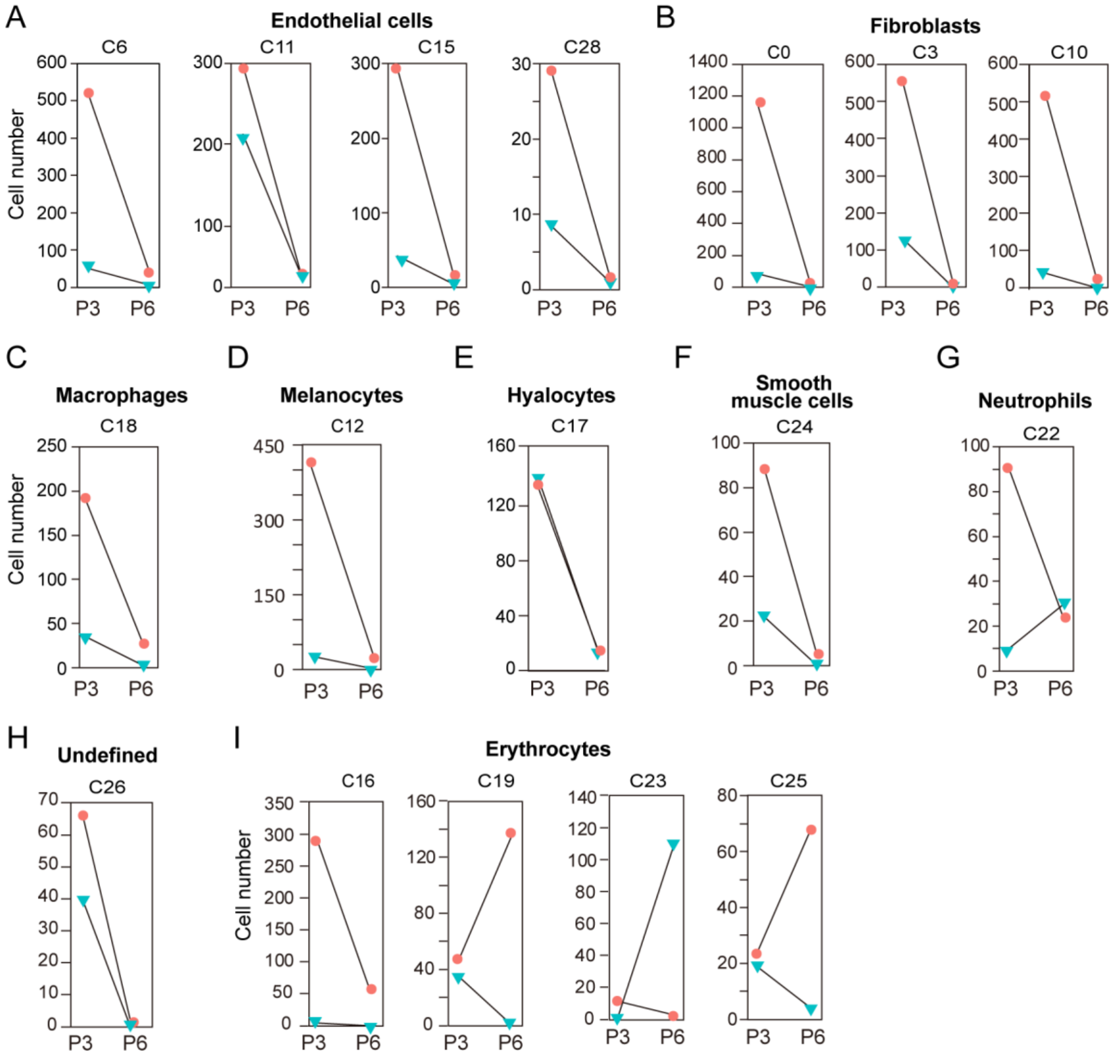

Supplemental Figure 3

Supplemental Figure 3. Clusters showing similar number sizes at P6 between the wild type and mutant vitreous. (**A**), Endothelial clusters. (**B**) Fibroblasts. (**C**), Macrophages. (**D**), Melanocytes. (**E**), Hyalocytes. (**F**), Smooth muscle cells. (**G**), Neutrophils. (**H**), Undefined. (**I**), Erythrocytes. Note that although enterocytes showed differences between the wild type and mutant vitreous, they are subjectively excluded from analysis because they are contained in the vessels and unlikely to affect vitreous cell regression.

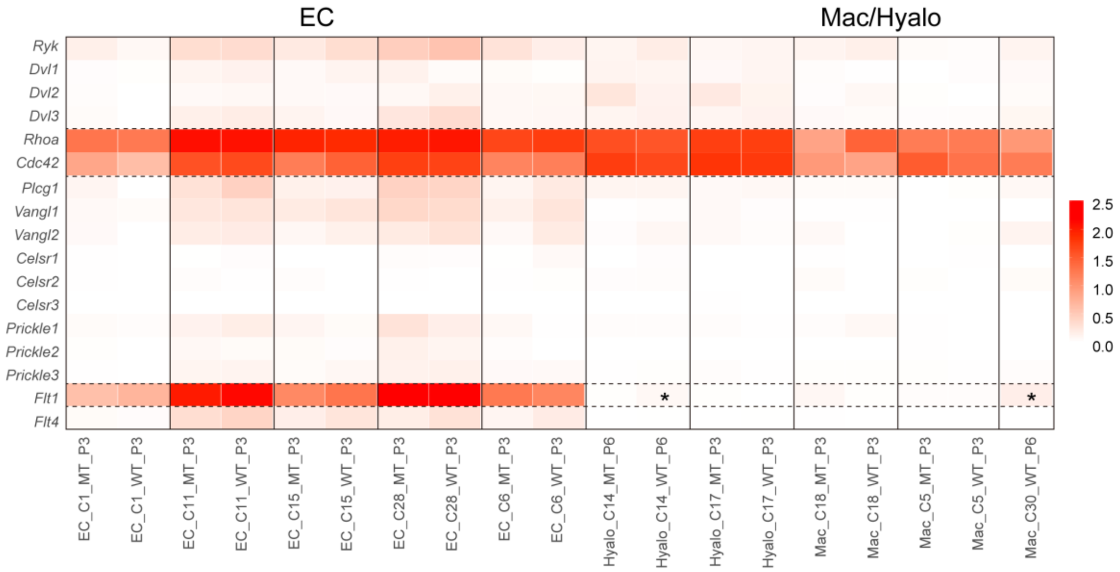

Supplemental Figure 4

Supplemental Figure 4. Expression of non-canonical Wnt components in EC and Mac/Hyalo clusters. Dashed boxes indicate Rhoa and Cdc42 and Flt expression. Asterisks indicate wild type Hyalo/Mac clusters expressing stronger Flt1.
